# Discovery of Novel Phenolic Compounds from *Eutypa lata* Through OSMAC Approach: Structural Elucidation and Antibiotic Potential

**DOI:** 10.3390/ijms26125774

**Published:** 2025-06-16

**Authors:** Ana Cotán, Inmaculada Izquierdo-Bueno, Abdellah Ezzanad, Laura Martín, Manuel Delgado, Isidro G. Collado, Cristina Pinedo-Rivilla

**Affiliations:** 1 Departamento de Química Orgánica, Facultad de Ciencias, Universidad de Cádiz, 11510 Puerto Real, Spain; ana.cotan17@gmail.com (A.C.); abdellah.ezzanad@gm.uca.es (A.E.); isidro.gonzalez@uca.es (I.G.C.); 2 Departamento de Biomedicina, Biotecnología y Salud Pública, Facultad de Ciencias del Mar y Ambientales, Universidad de Cádiz, 11510 Puerto Real, Spain; inmaculada.izquierdo@uca.es; 3 Instituto de Investigación en Biomoléculas (INBIO), Universidad de Cádiz, 11510 Puerto Real, Spain; 4 Área de Protección Vegetal, Centro de Investigaciones Científicas y Tecnológicas de Extremadura (CICYTEX), 06187 Guadajira, Spain; laura.martin@juntaex.es; 5 González Byass, S.A., 11402 Jerez, Spain; mdelgado@gonzalezbyass.es

**Keywords:** phytopathogen, GTDs, *Eutypa*, toxins, secondary metabolites

## Abstract

Among grapevine trunk diseases, Eutypa dieback, caused by the fungus *Eutypa lata*, is one of the most critical ones, due to its widespread infection in vineyards and the lack of effective treatments. This fungus is a vascular pathogen that enters grapevines through pruning wounds. The infection process is associated with phytotoxic metabolites produced by the fungus, and as such, the identification of new metabolites from different culture conditions and broths could provide valuable insights into the fungus’s enzymatic system and help its control. For the purposes of this study, the OSMAC (one strain, many compounds) approach was applied to investigate the secondary metabolism of *E. lata* strain 311 isolated from *Vitis vinifera* plants in Spain. A total of twenty metabolites were isolated, including five reported for the first time from *E. lata* and four that are newly identified compounds in the literature: eulatagalactoside A, (*R*)-2-(4′-hydroxy-3′-methylbut-1′-yn-1′-yl)-4-(hydroxymethyl)phenol, (*S*)-7-hydroxymethyl-3-methyl-2,3-dihydro-1-benzoxepin-3-ol, and (3a*R*,4*S*,5*R*,7a*S*)-4,5-dihydroxy-6-((*R*)-3′-methylbuta-1′,3′-dien-1′-ylidene)hexahydrobenzo[*d*][1,3]dioxol-2-one. These compounds were extracted from fermentation broths using silica gel column chromatography and high-performance liquid chromatography (HPLC). Their structures were elucidated through extensive 1D and 2D NMR spectroscopy, along with high-resolution electrospray ionization mass spectrometry (HRESIMS). Compounds were evaluated for phytotoxicity against *Phaseolus vulgaris*, with only eulatagalactoside A producing white spots after 48 h. Additionally, the antibacterial activity against *Escherichia coli*, *Staphylococcus aureus*, and *Klebsiella pneumoniae* of selected compounds was tested. The compounds (*R*)-2-(4′-hydroxy-3′-methylbut-1′-yn-1′-yl)-4-(hydroxymethyl)phenol and (*S*)-7-(hydroxymethyl)-3-methyl-2,3-dihydrobenzo[*b*]oxepin-3-ol showed the most significant antimicrobial activity against Gram-positive bacteria, inhibiting *S. aureus* by over 75%, with IC_50_ values of 511.4 µg/mL and 617.9 µg/mL, respectively.

## 1. Introduction

In recent years, grapevine trunk diseases (GTDs) have increasingly impacted modern viticulture, becoming a major destructive threat worldwide due to their complexity [1,2,3]. These diseases affect vineyards regardless of plant age [4], deteriorate the organoleptic properties of wines [5], and lead to significant economic losses by reducing the number of productive vines and increasing preventative management costs [4,6,7]. Toxic chemicals like arsenite have been traditionally used for plant protection, but this practice was banned in Europe due to its harmful effects on human health [8]. Since chemical fungicides have proven ineffective in the control of GTDs, recent research has been focusing on developing new strategies to manage the pathogens associated with these diseases [8,9,10].

The fungi responsible for grapevine trunk diseases (GTDs) have been extensively studied due to the increasing incidence of these diseases worldwide. The ability to control them is particularly challenging because of the internal development of the pathogen. Therefore, in order to effectively detect and combat these diseases, understanding the secondary metabolism of these fungi is crucial, as some metabolites may serve as diagnostic markers to predict and control the diseases in vineyards [11]. The causal agents of GTDs during their latent phase may behave as endophytic fungi [12], although the term “endophyte” has traditionally been associated with microorganisms that live inside plants without causing symptoms of disease and producers of substances that promote the growth of host plants. Recently, some studies have shown that endophytic fungi, depending on the environmental conditions and their interactions with the host, can be either pathogenic or nonpathogenic at certain stages of their life cycle [12,13]. These fungi strains are associated with the production of a significant number of natural products that exhibit a wide range of biological activities, such as anticancer, cytotoxic, antimicrobial, and anti-inflammatory properties. This suggests their potential as a clinical source of drugs [13,14,15]. Furthermore, the most extensively studied activity is their phytotoxicity [11,16,17], which is linked to the symptoms they produce in host plants.

**Figure 1:**
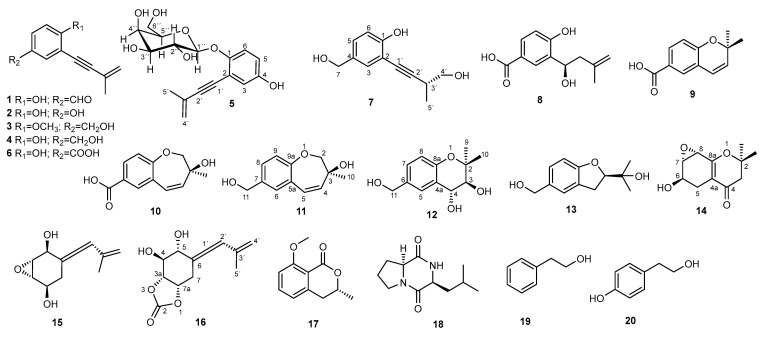
Isolated compounds from *E. lata* 311.

Eutypa dieback has been identified in many perennial woody plants [3], and it is one of the GTDs that has been raising increased interest in the last decade [18,19,20]. The economic impact of Botryosphaeria dieback and Eutypa dieback in California was estimated to be USD 260 million annually, while losses due to Eutypa dieback in southern Australia were reported to be AUD 2800 per ha [21]. This disease is caused by the fungus *Eutypa lata*, which infects and colonizes the xylem tissue from fresh pruning wounds and then spreads to the cambium and phloem of grapevine trunks [21,22,23]. Eutypa dieback can develop in the wood over years with no symptoms in the canopy [3]. External symptoms of Eutypa dieback usually appear on infested grape plants only a few years after the infection takes place, and they may present an “erratic” behavior in the subsequent years [8]. They are manifested by trunk necrosis in the wood and in the external elements by reduced growth of shoots, often with small, chlorotic leaves [3,8,22]. Eventually, the entire plant dies. The foliar symptomatology of Eutypa dieback is caused by the toxic metabolites produced by *E. lata* [11,24]. However, neither the annual canes nor the leaves of infected plants contain any mycelia. Thus, the fungus in the infected trunk seems to emit phytotoxic compounds to the distal parts of the plant. These compounds are secondary metabolites [25] or cell wall-degrading enzymes [23]. The known metabolites from *E. lata* are mainly acetylenic phenols [26] and their cyclic analogues [27] and some chromone derivatives [11,28]. Eutypine (**1**) (Figure 1) is considered the most toxic of them, but a study from Smith et al. revealed that the activity was related to the cyclization of **1** to 2-(prop-1-en-2-yl)benzofuran-5-carboxylic acid in the presence of traces of acid, this compound being more phytotoxic than **1** [24]. In addition to this, eulatachromene has been reported as phytotoxic [24,27]. Eutypinol (**4**) was the metabolite produced by different isolated of *E. lata* under most culture conditions. Other common derivatives isolated from cultures from several strains of *E. lata* were *O*-methyleutypinol (**3**) and eutypin carboxylic acid analogue (**6**) [29].

The disease caused by *E. lata* is far from being under control, since the traditional fungicides have been forbidden [8,9] and there are no alternative substances that are sufficiently effective [30]. As such, the study of the secondary metabolism of the fungus through an OSMAC approach could reveal the expression of silent biosynthetic genes and the production of cryptic metabolites, providing new information about their biosynthetic pathways to find new targets for developing new fungicides [31,32]. Moreover, secondary metabolites from fungi related to *E. lata* have shown interesting biological activities [33], renewing interest in metabolites from phytopathogenic fungi and not only the search for phytotoxins.

Strains of *E. lata* isolated from different locations can produce different profiles of metabolites, as reported for strains isolated from Australia or California [34,35,36], and it is possible to identify the infection by analyzing the presence of these metabolites *in planta* [26]. For the purposes of this research, the OSMAC approach was used for the first time to study the metabolite profile of a strain of *E. lata* isolated from vineyards in Spain, *E. lata* 311. This methodology helps us to compare the metabolite profile under different culture media and conditions. Two new polyphenols, a 2,3-dihydro-1-benzoxepine and allenic cyclohexane analogues, are described, and the absolute configuration studied using electronic circular dichroism (EDC): eulatagalactoside A (**5**), (*R*)-2-(4′-hydroxy-3′-methylbut-1′-yn-1′-yl)-4-(hydroxymethyl)phenol (**7**), (*S*)-7-hydroxymethyl-3-methyl-2,3-dihydro-1-benzoxepin-3-ol (**11**), and (3a*R*,4*S*,5*R*,7a*S*)-4,5-dihydroxy-6-((*R*)-3′-methylbuta-1′,3′-dien-1′-ylidene)hexahydrobenzo[*d*][1,3]dioxol-2-one (**16**). Compounds **2**, **4**, **5**, **7**, **10**, and **11** were tested for phytotoxicity against *Phaseolus vulgaris* and the dilution method was performed based on the reported activity of siccayne (**2**) as antibiotic [37] with compounds **2**, **4**, **5**, **7**, **8**, **11**, **12**, **13**, and **16** against model bacteria *Escherichia coli*, *Staphilococcus aureus*, and *Klebsiella pneumoniae*.

## 2. Results

The *Eutypa lata* fungus strain 311 was isolated from the inner wood core of *Vitis vinifera* L. cv. Tempranillo, planted in 2001. Using the OSMAC approach, the fungus was cultivated in various media and fermentation conditions to induce the expression of genes involved in new biosynthetic pathways or detoxification processes for its own toxins. Strain 311 was grown on various solid media [malt agar (MA), potato dextrose agar (PDA), and rice medium (RM)] and liquid media [Czapeck-Dok (CD) and potato dextrose broth (PDB)], with varying incubation times.

The mycelia were separated from the broths, which were subsequently extracted with ethyl acetate (EtOAc) and evaporated. The extracts were chromatographed, resulting in the isolation of 20 compounds (Figure 1). Metabolites **8**, **9**, **10**, **13** and **12** were isolated from this species for the first time, and four compounds, i.e., **5**, **7**, **11**, and **16**, are reported as new metabolites in this study. The known compounds were identified by means of an analysis of their spectra and the comparison of the spectroscopic data to the data available in the literature (Supporting Information, Figures S1–S7, S15, S16, S24–S29, S37–S45). Additionally, absolute configurations and phytotoxic and antimicrobial activities of the selected metabolites were determined.

The PDB medium was selected following references in the literature for the isolation of eutypine (**1**) [27,34], i.e., the most important toxin from *E. lata*. However, from this strain, only fatty acids and common compounds such as cyclo-(L-Pro-L-Leu) (**18**) [38], 2-phenylethan-1-ol (**19**) [39], tyrosol (**20**) [39], and (*R*)-8-*O*-methylmellein (**17**) (${\left[\alpha \right]}_{D}^{24}$-258.5 (c 0.13, CHCl_3_) (lit. [40] ${\left[\alpha \right]}_{D}^{15}$-250 (c 0.50, CHCl_3_)) were identified.

Purification of the Czapek–Dox (CD) medium extracts yielded the known compounds **2** [41], 14 [28], **15** [28], and 17 [40], as well as compound **12** [42], which is reported for the first time from *E. lata*, and the new galactopyranose derivative eulatagalactoside A (5), which has a similar structure to that of siccayne (2), in which one of the hydroxyl phenolic groups was glycosylated by a galactopyranose unit.

Compound **5** was also identified from a PDA medium, along with eutypine (**1**) [29] and eutypinol (**4**) [29]. This fermented outcome was kept both in light and in darkness for 20 days, in order to determine if light conditions would affect its metabolism, considering that the fungus grows inside the plant [26]. The difference noticed was that in darkness, instead of compound **14** [28], it was compound **15** [28] that was isolated.

**Figure 2:**
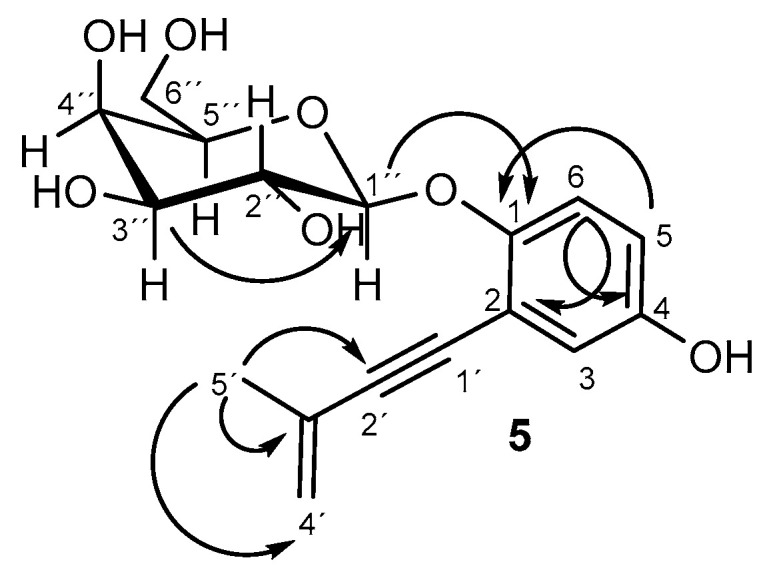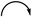 HMBC spectra correlations for **5**.

**Figure 3:**
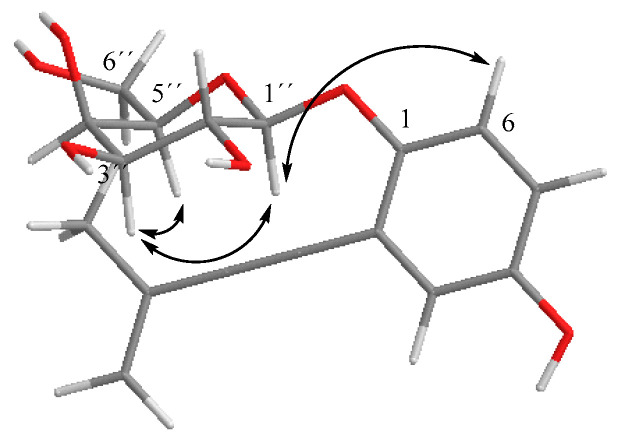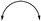 NOESY spectra correlations for **5**.

*β*-galactopyranose derivative **5** is described here for the first time. It was obtained as a yellow oil with the molecular formula C_17_H_20_O_7_ on the basis of the observed ion in its HRESIMS (*m*/*z* 337.1264 [M+H]^+^, calculated for C_17_H_21_O_7_, 337.1287). The ^1^H and ^13^C spectra (Table 1, Supporting information, Figures S8 and S9) were similar to those of the known siccayne metabolite (**2**) [41]. The difference was in the signals of the galactose unit, including anomeric proton at 4.81 ppm and three oxymethines at 3.81, 3.56, and 3.87 ppm. The final structure was assigned through gCOSY and gHMBC correlations (Figure 2, Supporting information, Figures S11 and S12). The gCOSY correlations between the anomeric proton (1″) and 2″ (3.81 ppm), H-2″ and H-3″ (3.56 ppm), H-3″ and H-4″ (3.87 ppm), and H-4″and H-5″ (3.60 ppm), HMBC correlation from H-1″ with the aromatic carbon at 152.2 ppm (C-4), and the NOESY spectrum cross-peak correlation (Supporting information, Figure S10) from H-1″ to H-6 suggest that the position of the sugar unit is at the phenolic hydroxyl group in C-1. Moreover, NOESY spectrum cross-peak correlations (Figure 3, Supporting information, Figure S10) from H-1″ to H-3″ and H-5″ confirmed the axial position of these protons, whereas *J*_3″4″_ of 3.4 Hz confirmed the equatorial position of H-4″, so the sugar unit was described as D-galactopyranose. The *β* configuration in C-1″ was established on the basis of the NOESY spectrum cross-peak correlations and value of *J*_1″2″_ of 7.7 Hz [43]. These data helped us conclude that compound **5** corresponds with 1-*O*-*β*-D-galactopyranosyl siccayne, which was named eulatagalactoside A (**5**).

**Figure 4:**
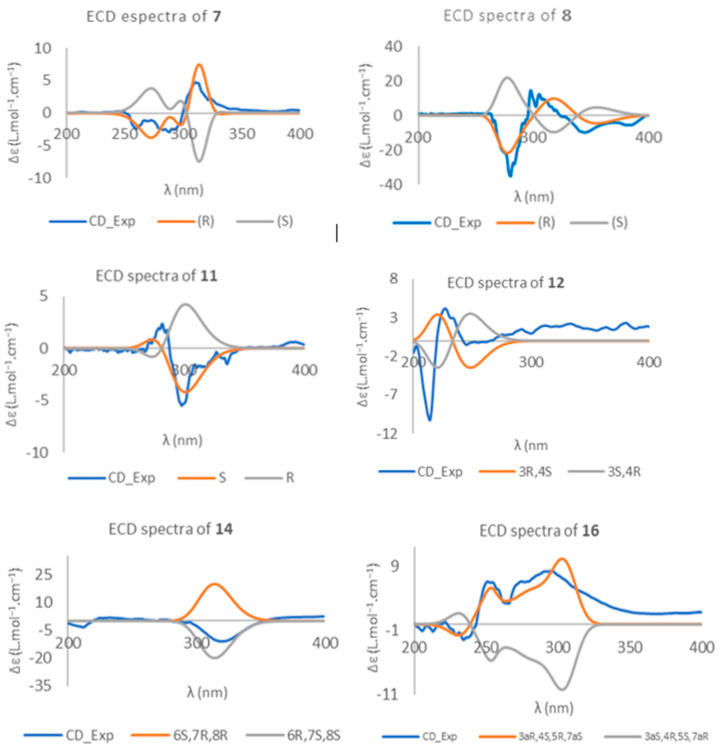
Experimental and calculated ECD spectra for compounds **7**, **8**, **11**, **12**, **14**, and **16**.

6-(Hydroxymethyl)-2,2-dimethyl-3,4-dihydro-2*H*-chromene-3,4-diol (**12**) [42] was previously reported from the marine sediment-derived fungus *Eutypella scoparia* FS26. In fact, it could be considered a derivative of eulatachromene [34]. The absolute configuration of **12** was also established using electronic circular dichroism (ECD) by comparing the theoretical spectrum with the experimental one (Figure 4) [44]. Thus, we described the structure for **12** as (3*S*,4*R*)-6-(hydroxymethyl)-2,2-dimethyl-3,4-dihydro-2*H*-chromene-3,4-diol [42].

6-Hydroxy-2,2-dimethyl-5,6,7,8-tetrahydro-7,8-epoxychroman (**14**) was isolated from *E. lata* by Renaud et al. [28]. Its relative configuration was deduced from ^1^H NMR coupling constants [28], but there was no information about its absolute configuration. As such, the absolute configuration of **14** was established by comparing the experimental electronic circular dichroism (ECD) spectrum of compound **14** with the ECD spectrum predicted from quantum mechanical time-dependent density functional theory (TD-DFT) calculations [44] and the calculated curve of **14** matched with the experimental one in the 300–350 nm region (Figure 4). In this study, we performed a conformational search to identify the most stable conformers prior to ECD spectral calculations. Dihedral angle scanning was carried out using the B3LYP functional and 6-311+G(2d,p) basis set for geometry optimization (see Section 4), allowing for an exploration of the potential energy surface and the identification of low-energy conformations relevant for ECD prediction.

The resulting energy profile showed a clear minimum around 180–200° of the enone dihedral angle, indicating a preferred and energetically stable conformation in that range. Consequently, the structure for compound **14** was designated (6*R*,7*S*,8*S*)-6-hydroxy-2,2-dimethyl-5,6,7,8-tetrahydro-7,8-epoxychroman (**14**).

Compounds **4**, **14** and **15** were the only metabolites identified from the solid culture in malt agar (MA) medium.

On the other hand, rice medium (RM) has proved to be the best medium for testing purposes due to the variability of the isolated secondary metabolites. New compounds **7**, **11**, and **16** and known compounds **3** [34], **4** [29], **6** [29], **8** [45], **9** [46], **10** [47], **13** [48], and **15** [28] were identified from this medium. Compounds **8**, **9**, **10**, and **13** were identified from *E. lata* for the first time in this study.

**Figure 5:**
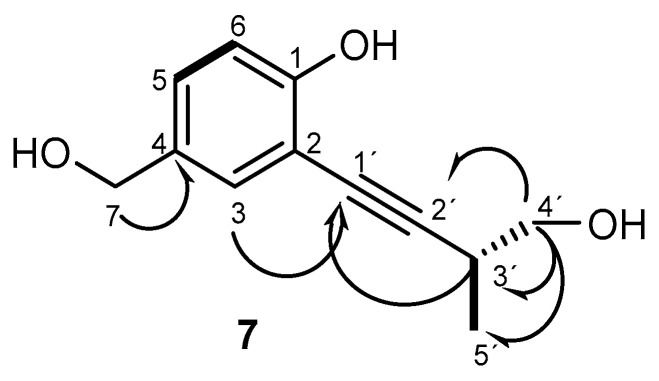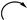 HMBC and 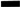 COSY spectra correlations for **7**.

Compound **7** is a phenol analogous to metabolite **4** [29], also being isolated only from RM. The double bond in the side chain of eutypine (**1**) was hydroxylated in compound **7** and was assigned the molecular formula C_12_H_14_O_3_ on the basis of the ions observed in its HRESIMS m/z 207.1027 [M+H]^+^ (calculated for C_12_H_15_O_3_, 207.1021). An ion at 189.0923 [M- H_2_O+H]^+^ (calculated for C_12_H_13_O_2_, 189.0916) refers to the loss of water, which was consistent with the proposed structure (Supporting Information, Figure S22a,b). The new phenol derivative 2-(4′-hydroxy-3′-methylbut-1′-yn-1′-yl)-4-(hydroxymethyl)phenol (**7**) presented one difference from **4**, i.e., the replacement of the exomethylene in C-4′ with a hydroxymethyl group. The ^13^C NMR and DEPT spectra (Table 2, Supporting Information, Figures S18 and S20) displayed three aromatic quaternary carbons (δ_C_ 156.5, 109.6, 132.6 ppm) and the characteristic signals from these groups: triple bond at δ_C_ 76.5 and 99.1, two oxymethylene sp^3^ (δ_C_ 64.7 and 66.7 ppm) and one methyl (δ_C_ 16.9 ppm). The ^1^H NMR spectrum (Table 2, Supporting Information, Figure S17) revealed, besides the presence of three aromatic protons of the 1,2,4 trisubstituted benzene ring at δ_H_ 7.31, (d(br), *J* = 2.1 Hz), 7.21 (dd, *J* = 8.3, 2.1 Hz), 6.91 (d, *J* = 8.3 Hz), a methyl doblet at δ_H_ 1.29 (d, *J* = 7.0 Hz), a singlet at δ_H_ 4.57 corresponding to the oxymethylene linked to the aromatic ring, and two protons of oxymethylene in position 3′ at 3.73 (dd, *J* = 10.3, 5.5 Hz) and 3.64 (dd, *J* = 10.3, 7.0 Hz) ppm. The gCOSY spectrum (Figure 4, Supporting Information, Figure S19) exhibited cross-peaks of H-3′ (2.96, m) to H-4′ (3.73, dd (*J* = 10.3, 5.5 Hz); 3.64, dd, *J* = 10.3, 7.0 Hz), of H-3′ to H-5′ (1.29, d, *J* = 7.0 Hz), and H-5 (7.21, dd, *J* = 8.3, 2.1 Hz) to H-6 (6.91, d, *J* = 8.3 Hz), confirming the presence of the 1,2,4-trisubstituted benzene ring and hydroxylated methylene in C-4′. This structure was corroborated by the gHMBC correlations (Figure 5, Supporting Information, Figure S21) of H-5 with C-1, C-3, and C-7; H-3′ with C-1′, C-2′, C-4′, and C-5′; H-4′ with C-2′, C-3′ and C-4′, and H-7 with C-4.

Absolute configuration was established by comparing the experimental electronic circular dichroism (ECD) spectrum of compound **7** with the predicted ECD spectrum (Figure 4) [44]. Thus, the proposed structure for **7** is (*R*)-2-(4′-hydroxy-3′-methylbut-1′-yn-1′-yl)-4-(hydroxymethyl)phenol.

Compound **8** was previously reported for the dark septate fungal endophyte *Drechslera* sp. [45], and anofinic acid (**9**) from fungi *Lactarius deliciosus* and *Curvularia fallax* [49,50]. An ECD study was performed to assign the absolute configuration of **8**, resulting in (*R*)-4-hydroxy-3-(1-hydroxy-3-methylbut-3-en-1-yl)benzoic acid (**8**) (Figure 4). Compound **8** is a flexible molecule with several conformers, so in order to determine the more stable conformation, we carried out a relaxed scan of the key dihedral angle using the B3LYP functional with the 6-311+G(2d,p) basis set (see Section 4). Several low-energy minima were observed, especially around 170–180° and 300–310°, indicating the presence of preferred and energetically favorable conformations in these ranges.

3-Hydroxy-3-methyl-2,3-dihydro-1-benzoxepine-7-carboxylic acid (**10**) was identified from marine-derived fungus *Neosartorya quadricincta* KUFA 0081 [47]. Interestingly, the physical and spectroscopic properties of **10** were coincidental with the compound reported as quadricinctoxepine. The optical rotation value for compound **10** in the literature was ${\left[\alpha \right]}_{D}^{20}$ + 21 (c 0.07, MeOH), and the optical rotation for the isolated compound in this research was ${\left[\alpha \right]}_{D}^{21}$ + 38.5 (c 0.05, CHCl_3_), suggesting an *R* configuration at C-3 based on the reported data. In terms of to the absolute configuration of its derivative, **11** (Figure 4) (discussed later), its ECD spectrum was compared with the ECD spectrum obtained for derivative **11** (Supporting Information, Figure S36), allowing assignment of the *S* configuration at C-3.

**Figure 6:**
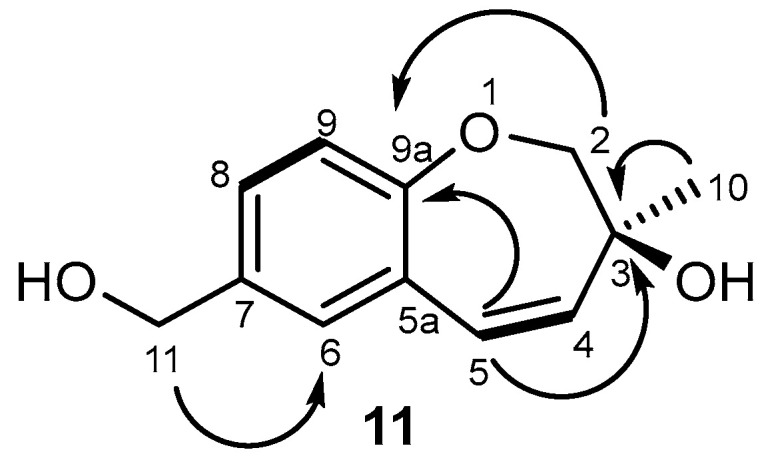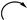 HMBC and 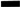 COSY spectra correlations for **11**.

Compound **11** has never been reported in the past, so it is described here for the first time. Its chemical structure was similar to that of compound **10**. Compound **11** was isolated as a yellow oil, and its molecular formula, i.e., C_12_H_14_O_3_, was determined based on the (+)-HRESIMS m/z 207.1026 [M+H]^+^ (calculated 207.1021). The IR spectrum showed absorption bands for hydroxyl (3367 cm^−1^), a double bond C=C (1795 cm^−1^), aromatic (1499 cm^−1^), and olefin (1225 and 1122 cm^−1^) groups. The ^13^C and gHSQC spectra (Table 3, Supporting Information, Figures S31 and S32) exhibited the signals of two oxymethylene sp^3^ (δ_C_ 78.5 and 64.8 ppm), two olefinic sp^2^ methines (δ_C_ 136.5, 126.5 ppm), three quaternary sp^2^ (δ_C_ 158.4, 126.3, 135.6 ppm), three aromatic sp^2^ methines (δ_C_ 131.5, 127.8, 120.2 ppm), one O-substituted tertiary sp^3^ (δ_C_ 72.6 ppm), and one methyl (δ_C_ 24.1 ppm) groups. The ^1^H NMR spectrum (Table 3, Supporting Information, Figure S30) revealed the presence of three aromatic protons of the 1,2,4 trisubstituted benzene ring at δ_H_ 7.02, d (*J* = 8.0 Hz); 7.20, dd (*J* = 8.0, 2.3); and 7.19, d (br) (*J* = 2.3 Hz) ppm; two doublets of the protons of a *cis*-double bond at δ_H_ 6.25, dd (*J* = 11.9, 0.6 Hz) and 5.93, dd (*J* = 11.9, 1.7 Hz) ppm; a methyl singlet at δ_H_ 1.33 ppm; a singlet of two protons at δ_H_ 4.63 ppm; and two signals of methylene protons bound to oxygen at δ_H_ 4.12, dd (*J* = 11.9, 1.8 Hz) and 3.87, d (*J* = 11.9 Hz) ppm. The presence of a 1,2,4-trisubsituted benzene ring and the *cis*-double bond was confirmed by the gHMBC correlations (Figure 6, Supporting Information, Figure S33) of H-9 with C-7 and C-5a; H-6 with C-8 and C-9a; H-4 with C-10 and C-2; H-2 with C-9a and C-4; and of H-5 with C-3 and C-6. The oxepin ring structure and the position of the methyl group and the hydroxyl group on C-3 were also confirmed by the HMBC correlations of the methyl singlet at δ_H_ 1.33 ppm (H-10) with C-3 (δ_C_ 72.6), H-2 (δ_H_ 4.12 and 3.87 ppm), and C-9a (δ_C_ 158.4 ppm); and H-10 with C-2 (δ_C_ 78.5 ppm) (Figure 6).

Compound **11** displayed a positive optical rotation value (${\left[\alpha \right]}_{D}^{21}$+ 89.5 (c 0.5, CHCl_3_)), which was the same sign as that of compound **10** (${\left[\alpha \right]}_{D}^{21}$ + 38.5 (c 0.05, CHCl_3_)), suggesting initially the same configuration in C-3 ((*R*)-stereoisomer). However, the configuration at C-3 in compound **10** was determined by empirical calculations by Prompanya et al. [47]. In order to determine and confirm the absolute configuration of **11**, prediction of the ECD spectrum was undertaken and the experimental and simulated spectra showed the *S* configuration at C-3 (Figure 4). These results lead us to propose that the configuration of compound **10** should be revised, as it is (*S*)-stereoisomers for both compounds **10** and **11** (Supporting Information, Figure S36).

In addition, **13** was identified as (2*R*)-2-(5-hydroxymethyl-2,3-dihydrobenzofuran-2-yl)propane-2-ol, previously isolated for fungus *Xylaria acuminatilongissima* YMJ623 [48]. *R* configuration was assigned by comparison of the data to those obtained by Cho et al. [48] (${\left[\alpha \right]}_{D}^{24}$-10.5 (c 0.17, CHCl_3_) (lit. [47] ${\left[\alpha \right]}_{D}^{26}$ -19.5 (c 0.60, MeOH)).

**Figure 7:**
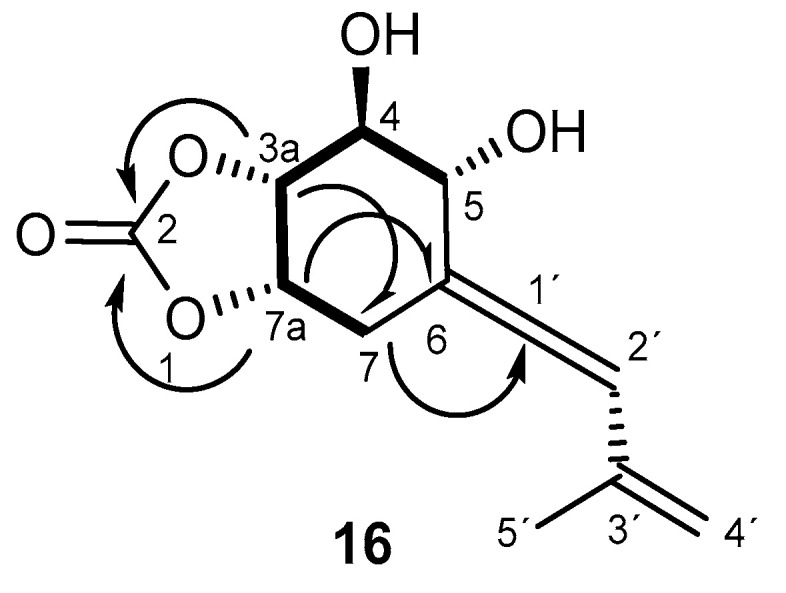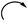 HMBC and 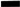 COSY spectra correlations for **16**.

**Figure 8:**
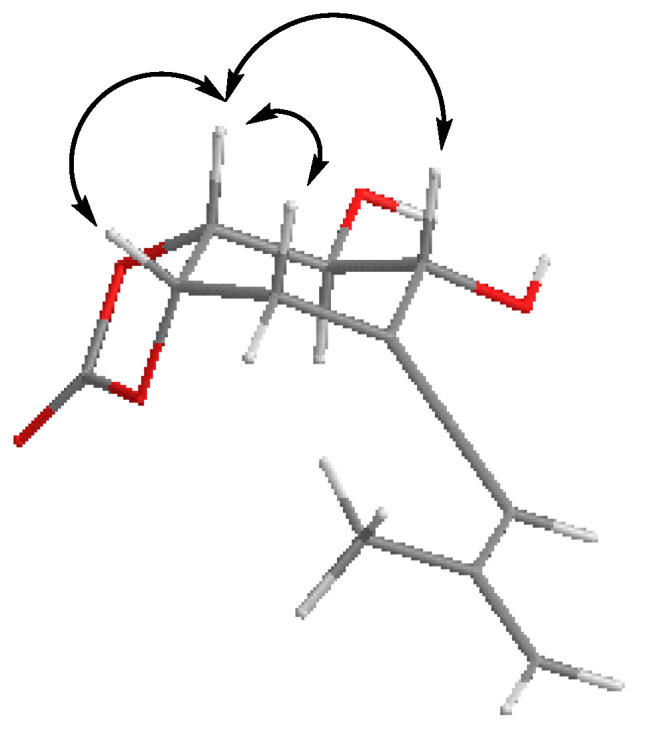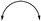 Selected NOE interactions and correlations exhibited by **16**.

Compound **16** was obtained as a colorless oil, the ^13^C NMR spectroscopic data of which were consistent with a cyclic carbonate derivative with formula C_12_H_14_O_5_ from its molecular ion at HRESIMS, m/z 237.0763 [M-H]^+^ (calculated for C_12_H_13_O_5_ 237.0760) (Supporting Information, Figure S51). There are a few examples of this type of structure in nature. The first report of natural cyclic carbonate in terpenoids was in 2006 [51], when it was isolated from the plant *Centaurea hololeuca* and cyclohexanoids similar to **16** were isolated from the fungus *Parastagonospora nodorum* SN15 [52]. The ^I3^C NMR spectrum of **16** (Table 4, Supporting Information, Figure S47) was very similar to that of compound **15** [28], except for the presence of a signal at δ_c_ 154.0 ppm corresponding to the carbonyl group of carbonate. Moreover, the ^13^C NMR and DEPT spectra (Table 4, Supporting Information, Figures S47 and S49) exhibited the presence of five methines, four of them bound to oxygen, one methylene and one methyl groups, and four quaternary carbons, one of which corresponded to a cyclic carbonate group (δ_C_ 154.0 ppm). The ^1^H NMR spectrum (Table 4, Supporting Information, Figure S46) showed, in addition to the signals for four methine groups bound to oxygen at δ_H_ 3.82 (1H, dd, *J* = 9.0, 7.0 Hz), 4.04 (1H, m), 4.62 (1H, td, *J* = 7.0, 0.6 Hz), and 4.90 (1H, ddd, *J* = 7.0, 4.7, 3.2 Hz) ppm, a signal characteristic of one proton on a trisubstituted double bond at δ_H_ 6.30 (1H, t, *J* = 3.5) ppm. The IR spectrum (Supporting Information, Figure S52) showed an absorption band at 1798 cm^−1^ and the quaternary carbon in the ^I3^C NMR spectrum at 200.7 ppm supported the hypothesis of the allenic group. The gCOSY spectrum (Figure 7, Supporting Information, Figure S48) showed correlations between H-7a (δ_H_ 4.90 ppm) and H-7α, H-7β (δ_H_ 2.94 and 2.66 ppm) and H-3a (δ_H_ 4.62 ppm); H-3a and H-4 (δ_H_ 3.82 ppm); and H-4 and H-5 (δ_H_ 4.04 ppm), revealing the vicinal relationship of the hydroxylated positions. gHMBC (Figure 6, Supporting Information, Figure S50) revealed correlations, among others, between H-3a and C-2, C-7, and C-4; H-7a and C-6, C-3a; and between H-7α, H-7β and C-5, C-6, C-7a, and C-3a, C-1′ locating the cyclic carbonate between C-3a and C-7a. The relative configuration was established by NOE and NOESY experiments (Figure 8) (Supporting Information, Figures S53 and S54). The spatial disposition of the vinyl(methyl)allenic group for **16** was inferred from NOE interactions and comparison of spectroscopic data with those of compound **15**, where the geometry of the vinyl-allenic group was confirmed by X-ray and stereospecific total synthesis [28,53].

The NOESY correlations (Supporting Information, Figure S54) between H-3a and H-7a and H-5 and the correlations observed between H-7a and H-3a, H-7α and H-7β suggested that H-3a/H-5 were on the same plane of the six-membered ring, as well as the protons geminal to the cyclic carbonate. Values of *J*_3a,4_ of 7.0 Hz and *J*_4,5_ of 9.0 Hz confirmed the axial position of H-4, so the (3a*R*,4*S*,5*R*,7a*S*) relative configuration was inferred for the stereogenic carbons of compound **16**. In order to determine the absolute configuration of this compound, prediction of the ECD spectrum was undertaken, and the experimental and simulated spectra confirmed the configuration as (3a*R*,4*S*,5*R*,7a*S*)-4,5-dihydroxy-6-((*R*)-3′-methylbuta-1′,3′-dien-1′-ylidene)hexahydrobenzo[*d*][1,3]dioxol-2-one (**16**), (Figure 4 and Figure 8).

### In Vitro Phytotoxic and Antimicrobial Assays

**Figure 9:**
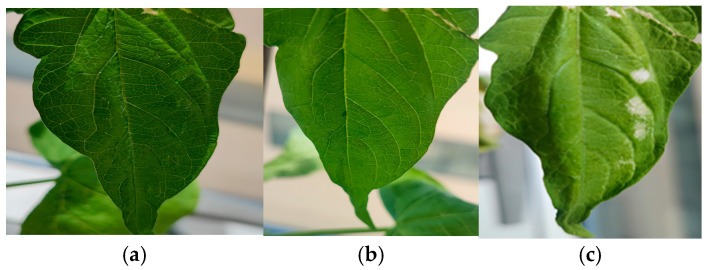
Phytotoxicity assay of **5** after (**a**) 0 h; (**b**) 24 h and (**c**) 72 h at 1000 ppm.

Since compounds **7** and **16** were obtained in low amounts, only compounds **5**, **7** and **11** of the new derivatives isolated were tested pure for phytotoxicity. For **8**, **13** and **16**, the fractions Fr7 peaks 13 and 15 and Fr 8 from rice medium were tested as fractions where the metabolites were identified. The results showed that none of them was phytotoxic, except **5**, which showed white spots after 72 h at a concentration of 1000 ppm (Figure 9).

**Figure 10:**
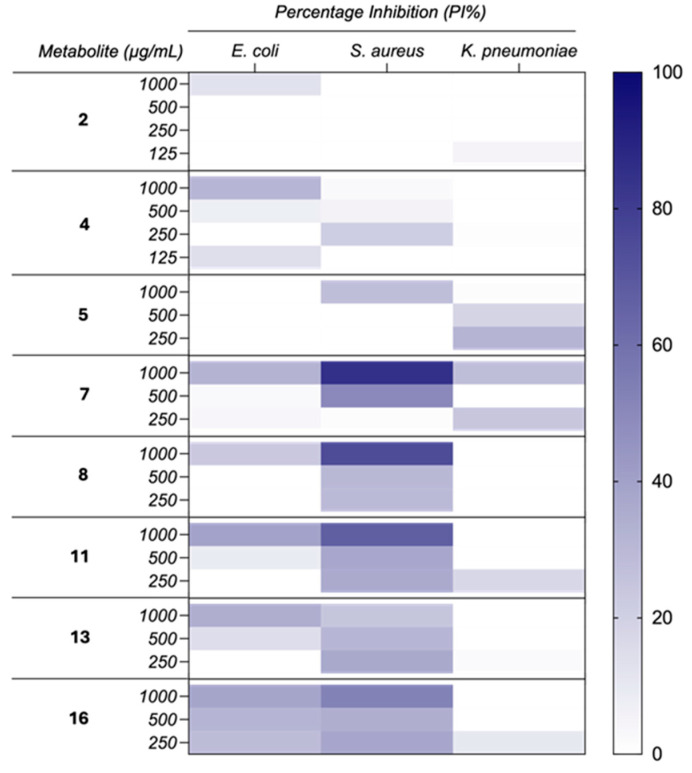
Heatmap plot of the percentage inhibition (PI) of the isolated compounds against different microbial strains. All determinations were performed in triplicate and values are expressed as the means of replicates.

Additionally, compounds **2**, **4**, **5**, **7**, **8**, **11**, **13**, and **16** were subjected to antimicrobial assays against microbial human pathogens, including Gram-negative bacteria (*Escherichia coli* CECT434 and *Klebsiella pneumoniae* CECT7787) and Gram-positive bacteria (*Staphilococcus aureus* CECT794). The tested compounds demonstrated no notable activity against Gram-negative strains (IC_50_ > 1000 µg/mL). However, moderate activity was identified against Gram-positive bacteria (Figure 10). It is worth noting that at no point did the percentage of inhibition exceed 90%. Accordingly, IC_50_ values were calculated for the most significant percentages to obtain a more accurate measurement of the compound’s efficacy.

Compounds **7** and **8** demonstrated the most significant antimicrobial activity for Gram-positive bacteria, showing inhibition values above 75% against *S. aureus* (85.27% and 74.3% at 1000 ppm), with measured IC_50_ of 511.4 µg/mL and 617.9 µg/mL. This result is consistent with previous findings for analogous compounds derived from *E. lata*. For instance, a study reported that eutypinol (**4**), a compound analogous to **7**, exhibited robust antibacterial activity against *S. aureus* with an MIC of 31.25 µg/mL, yet demonstrated no activity against *E. coli* (MIC > 1000 µg/mL) [54]. The discrepancy in inhibitory capacity may be related to the incorporation of an additional hydroxyl group in C-1′ of compound **8**.

Similarly, compounds **11** and **16** exhibited moderate activity against *S. aureus* (67.34% and 52.37% at 1000 ppm), with a measured IC_50_ of 583.1 µg/mL and 1091 µg/mL, respectively. However, they demonstrated weak inhibitory capacity against *E. coli* and were ineffective against *K. pneumoniae*.

The results showed that none of the compounds exhibited significant inhibition against Gram-negative *K. pneumoniae*. It is worth noting that compound **5** demonstrated slight inhibition only at low concentrations during the assays. The antimicrobial activity data for this metabolite against *K. pneumoniae* at varying concentrations showed a non-standard response pattern consistent with the “Eagle effect,” where higher antibiotic concentrations result in reduced efficacy [55,56]. At 1000 ppm, inhibition was minimal (1.23%), whereas it increased significantly at 500 ppm (18.35%) and reached a maximum at 250 ppm (31.81%). This unexpected reduction in efficacy at higher concentrations can be explained by mechanisms such as bacterial tolerance, saturation of target sites, and negative interactions at higher concentration levels. These findings underscore the importance of optimizing antimicrobial concentrations, since increasing the dosage may not always enhance efficacy and can sometimes be counterproductive [55,57].

## 3. Discussion

The current research provides the first report of the study of the secondary metabolites from a strain of *E. lata* isolated from *Vitis vinifera* in Spain. Since the differences in the production of secondary metabolites from strains of *E. lata* isolated from different locations was reported by Molyneux et al. [34], the study of several strains of *E. lata* could provide new information about the biosynthetic pathways and new toxins involved in the infection process.

OSMAC approach has emerged as a useful technic to isolate new cryptic metabolites based on changing the culture media and fermentation conditions [58]. This approach has been used to study the secondary metabolism of the strain *E. lata* 311 allowing the identification twenty metabolites. Compounds **8**, **9**, **10**, **12** and **13** were isolated from this species for the first time, and four compounds, i.e., **5**, **7**, **11**, and **16**, were reported as new metabolites. The known metabolites were identified by comparison of their spectroscopic (essentially ^1^H and ^13^C NMR) data with the data previously reported.

From PDB crude extracts, only common metabolites in fungi metabolism such as (*R*)-8-*O*-methylmellein (**17**), cyclo-(L-Pro-L-Leu) (**18**), 2-phenylethan-1-ol (**19**), and tyrosol (**20**) [45,59,60] were isolated. The phytotoxicity of **17** and **19** on tomato cuttings and grapevine leaves is well known [61,62]. So, the identification of the metabolites could mean that they are involved in the infection process, together with the specific toxin eutypine (**1**). Compound **1** was only identified in minimal amounts from a PDA medium, with eutypinol (**4**) being the major metabolite isolated from this culture. This is consistent with results in the literature, where **4** resulted from the reduction of the aldehyde group in structure **1** to an alcohol [29].

On the other hand, siccayne (**2**) was the major metabolite being isolated for both static and shaken conditions from the Czapek–Dox (CD) broth. Compound **2** presented moderate antibiotic activity against Gram-positive bacteria and *Saccharomyces cerevisiae* [25] and showed cytotoxic activity against human cancer cell lines HeLa and HT29 [37,63]. Siccayne (**2**) appears to interfere with the uptake of nucleoside precursors into eukaryotic cells, as well as with the in vitro incorporation of nucleotides into DNA. This is an interesting fact, because this strain could be used as an enzymatic model to obtain biological active derivatives of siccayne (**2**) through biotechnological uses.

Eulatagalactoside A (**5**) is a new galactopyranose derivative, in which one of the hydroxyl phenolic groups from the siccayne (**2**) has been etherified by a galactopyranose unit. It was isolated from Czapek–Dox and PDA media. This compound is described here for the first time, and some examples of these types of compounds isolated from fungi are the conoideoglucoside A and a phenolic glucoside isolated from insect fungus *Conoideocrella krungchingensi* [64]. Another example of these types of compounds is cordyceglycoside A, identified from *Cordyceps militaris* [65]. Interestingly, these compounds showed a broad range of biological activity, such as antimalarial, antiviral, antibacterial, and cytotoxic, and isolation of compound **5** from *E. lata* 311 could indicate the presence of enzymes such as glycoside hydrolases [66]. This compound showed phytotoxicity in *Phaseolus vulgaris* leaves after 72 h, when chlorotic spots appeared on the leaves (Figure 9). The effects must be visible after 2–6 h, but although the longer bioassay period and the sunlight are likely to be related to chlorophyll loss, the other leaves that were treated with other compounds and controls did not show symptoms, so we could confidently say that they presented phytotoxicity.

PDA medium fermentation was kept for 20 days, both in light and dark conditions. The difference noticed was that in dark conditions, instead of compound **14**, it was compound **15** that was isolated. Compound **14** is considered to be the cyclization product of **15**, which suggests that the inactivation of the enzyme responsible for cyclization occurred while the compound was in dark conditions [28]. Compound **14** showed moderate antimicrobial activity against *Salmonella Setubal* [54].

The metabolites identified for the first time for this strain, i.e., **8**, **9**, **10**, **12** and **13**, offer new information about the biosynthetic pathways of this strain. Compound **8** was identified for the first time from the fungus *Drechslera* sp. and displayed good antifungal activity against *Fusarium tucumaniae* [45]. It is worth noting that anofinic acid (**9**) was isolated for the first time from *Anodendron affine* and *Piper aduncum* [46,67], which is consistent with the evidence shown in the literature that confirms that some endophytic fungi produce metabolites that are analogous to those produced by host plants [12].

In addition, **12** was isolated from the marine sediment-derived fungus *Eutypella scoparia* FS26, but Sun et al. [42] only gave its relative configuration. We determined its absolute configuration by ECD, designating the structure (3*S*,4*R*)-6-(hydroxymethyl)-2,2-dimethyl-3,4-dihydro-2*H*-chromene-3,4-diol (**12**). Compound **13** is also described here for the first time for *E. lata* and presented a structure related to 5-formyl-2-(methylvinyl)[1]llbenzofuran described by Renaud et al. [29] as a product of the cyclization of **1**.

Rice medium (RM) has proven to be the best medium for testing purposes, due to the variability of secondary metabolites being isolated, which aligns with several examples in the literature [68,69]. New compounds **7**, **11**, and **16** were identified from this medium. Compound **7** is a phenol, analogous to metabolite **4**. Compound **6** was also isolated only from RM. Multiple bonds were hydroxylated in compound **7**, the double bond in the side chain of eutypine (**1**). Additionally, compound **11** is analogous with compound **10**, which was not isolated from this strain. These compounds revealed the range of oxygenases present in the fungus.

Compound **16** could be biosynthetically related to 8-hydroxy-2,2-dimethyl-5,6,7,8-tetrahydro-6,7-epoxychroman-4-one reported by Renaud et al. [28], an isomer of **14**. It contained a cyclic carbonate, a moiety rarely reported in natural products. The first report of this group was a terpenoid from the plant *Centaurea hololeuca* [51], more closely related to compound **16**, while cyclohexanoids were isolated from RM of phytopathogenic fungus *Parastagonospora nodorum* SN15 [52]. This compound was particularly interesting, as it suggested that this strain possesses the enzyme responsible for the cycloaddition of CO_2_ with epoxides, producing five-membered cyclic carbonates [70,71]. These compounds have various applications, such as serving as aprotic polar solvents and precursors for producing polycarbonates [71].

The new compounds **7** and **11** could not be tested as phytotoxins because they were isolated in low amounts, so the fractions in which they were identified, instead of the pure compounds, were tested. However, according to the structure–activity relationship studies carried out by Smith et al. [24] and Renaud et al. [28], these compounds are not expected to have phytotoxic properties. In addition to this, this study showed that **1** and **4** are the most active metabolites with an acetylenic side chain. The fractions tested were Fr7 peaks 13 and 15 from RM. The results showed that the compounds and the fractions are not phytotoxic.

Considering that **2** showed antibiotic activity against Gram-positive bacteria and some fungi and a cytotoxic effect on both normal and carcinoma cells [37], the new compounds were also tested against three bacteria, namely *Escherichia coli*, *Staphilococcus aureus*, and *Klebsiella pneumoniae*. The results demonstrated that the compounds isolated from *E. lata* exhibited greater efficacy against the Gram-positive *S. aureus* strain compared to the Gram-negative *E. coli* and *K. pneumoniae* strains (Figure 9). This finding is in line with the existing literature, which suggests that Gram-positive bacteria are typically more susceptible to antimicrobial agents due to their comparatively simple cell wall structure [72,73].

In summary, the results of this study are in line with the existing knowledge about the production of secondary metabolites by *E. lata*. Indeed, eulatinol (**4**) was produced almost exclusively on artificial media, whereas eutypine (**1**) and eulatachromene (not identified for this strain) were more abundant on grapevine extract medium [26]. Although phytotoxicity is absent in specific metabolites, this strain is infective due to the production of **17** and **19** and phenolic metabolite **1** [61,74]. Additionally, the identification of new metabolites never isolated for *E. lata* makes new possibilities such as chemical markers very useful to determine the presence of this phytopathogen *in planta* before foliar symptoms become visible.

## 4. Materials and Methods

### 4.1. General Experimental Procedures

ECD spectra were recorded on a JASCO J-1500 CD spectrometer (Tokyo, Japan). Optical rotations were determined with a JASCO P-2000 polarimeter. IR spectra were recorded on a PerkinElmer Spectrum BX FT-IR spectrophotometer (Waltham, MA, USA) and reported as wavenumbers (cm^−1^). TLC was performed on a Merck Kiesegel 60 F_254_(Rahway, NJ, USA), 0.25 mm thick. Silica gel 60 PF_254_ (60–100 mesh, VWR) was used for column chromatography. HPLC was performed with a Hitachi/Merck L-6270 apparatus equipped with a UV-vis detector (L 4250) and a differential refractometer detector (RI-7490) (Chiyoda, Japan). LiChroCART LiChrospher Si 60 (5 µm, 250 mm × 4 mm) and LiChroCART LiChrospher Si 60 (10 µm, 250 mm × 10 mm) columns were used for isolation experiments. ^1^H and ^13^C NMR measurements were recorded on Bruker 400, 500, and 700 MHz spectrometers with SiMe_4_ as the internal reference. Chemical shifts are expressed in ppm (*δ*), referenced to CDCl_3_ (Eurisotop, Saint-Aubiu, France, *δ*_H_ 7.25, *δ*_C_ 77.0) and CD_3_OD (Eurisotop, Saint-Aubiu, France, *δ*_H_ 3.30, *δ*_C_ 49.0). COSY, HSQC, HMBC, and NOESY experiments were performed using standard Bruker pulse sequence NMR assignments using a combination of 1D and 2D techniques (Billerica, MA, USA). High-resolution mass spectroscopy (HRMS) was performed with a double-focusing magnetic sector mass spectrometer in a QTOF mass spectrometer in either the positive-ion or negative-ion ESI mode.

### 4.2. Fungal Material

The phytopathogenic *Eutypa lata* strain 311, which was provided by the collection of the Centro de Investigaciones Científicas y Tecnológicas de Extremadura (CICYTEX), was isolated from an internal wood core of *Vitis vinifera* L. cv. Tempranillo planted in 2001 and grafted onto 110 Richter. The vineyard was in Finca La Orden, Badajoz, southwestern Spain (lat. 38°51′38″ N; long. 6°40′0″ W; altitude 198 m). The wood sample was collected using a Pressler’s borer and then analyzed following the culture-dependent method. Morphological characterization and subsequent molecular identification were performed as previously described [75]. Total genomic DNA was isolated and amplified from fresh mycelium of a pure culture using the REDExtract-N-Amp Kit (XNAP) (Sigma, St. Louis, MO, USA) and PCR amplifications were performed using a T100™ thermal cycler (BioRad Laboratories, Inc. Hercules, CA, USA) with the primer sets ITS4 (5′-TCCTCCGCTTATTGATATGC-3′) and ITS5 (5′-GGAAGTAAAAGTCGTAACAAGG-3′) [76]. The sequences were subsequently read, edited and compared with those in the NCBI GenBank database (https://blast.ncbi.nlm.nih.gov/) (accessed on 23 September 2024). The results confirmed the identity of the phytopathogenic species *E. lata* (99.8% similarity to KU320617 and 99.6% to MG745836).

This culture was also deposited at the University of Cádiz’s Mycological Herbarium Collection (UCA). Conidial and mycelium stock suspensions were maintained viable in 80% glycerol at −40 °C and −4 °C.

### 4.3. General Culture Conditions

An *E. lata* 311 strain was grown in petri dishes with PDA medium for one week at 25 °C in white light (daylight lamp) conditions. After extraction and solvent evaporation, extracts were analyzed by thin layer chromatography (TLC) in order to determine the polarities and fractions to collect by column chromatography (CC).

Subsequently, for liquid media, 5 pieces of agar (~1 cm) containing mycelium were added to Roux bottles for surface cultures or Erlenmeyer flasks for shaken cultures with PDB (Condalab, Madrid, Spain) or Czapek–Dox medium (50 g glucose, 1 g yeast extract, 5 g K_2_HPO_4_, 2 g NaNO_3_, 0.5 g MgSO_4_·7H_2_O, 0.01 g FeSO_4_·7H_2_O, 1 L of water and pH adjusted to 6.5–7.0). The shaken cultures were incubated in an orbital shaker at 120 rpm at 25 °C under a white light.

Fermentation continued for 17 days in some cases and for 30 days in others, after which the mycelium was filtered. The broth was extracted three times with ethyl acetate (EtOAc) and the extract dried over anhydrous Na_2_SO_4_. The solvent was subsequently evaporated and the residue chromatographed, first on a silica gel column and then by HPLC with an increasing gradient of *n*-hexane to ethyl acetate.

In the case of solid culture media, 1 L of sterile PDA (Condalab, Madrid, Spain), MA (20 g glucose, 20 g malt extract, 20 g agar, and 1 g peptone, per liter of water, pH 6.5−7) were placed in petri dishes 150 mm in diameter. Then, 1 piece of agar containing mycelium was added to the center of the plate and the fermentation ran for 21 days (until the fungus was fully grown on the plate) under a white light. The same process was followed for fermentation in darkness conditions.

A rice medium (80 g white rice per 100 mL of water) was placed in twenty Erlenmeyer flasks (500 mL), soaked overnight before sterilizing, and incubated for 40 days at 25 °C under a white light. The agar was cut into small pieces and rice removed and extracted three times with ethyl acetate in an ultrasound bath for 15 min. The extracts were filtered and dried over dry Na_2_SO_4_. The solvent was later evaporated and the residue chromatographed, first on a silica gel column and then by HPLC with an increasing gradient of ethyl acetate to *n*-hexane.

### 4.4. Liquid Culture Fermentation

#### 4.4.1. PDB Medium Fermentation

An *E. lata* 311 strain was incubated in 6 Roux bottles (150 mL per bottle) for 17 and 30 days. Evaporation of the solvent at reduced pressure produced extracts in the form of yellow oils, 29.57 mg (17 days fermentation) and 32.32 mg (30 days fermentation).

Crude extracts were applied on a column of silica and eluted with mixtures of *n*-hexane-ethyl acetate and EtOAc:MeOH; 100 mL fractions were collected as follows: Fr1 (*n*-hexane-ethyl acetate, 5:5) (6.79 mg, 17 d and 6.51 mg, 30 d), Fr2 (*n*-hexane-ethyl acetate, 8:2) (1.74 mg, 17 d and 0.99 mg, 30 d), and Fr3 (EtOAc-MeOH, 9:1) (9.54 mg, 17 d and 3.79 mg, 30 d). After 17 d, Fr1 gave the known compound diketopiperazine cyclo (L-Pro-L-Leu) (**18**) (1.99 mg) [77], and purification of the extract fermented for 30 d produced the known compound (*R*)-*O*-methylmellein (**17**) [40] (2.49 mg).

In the case of the shaken cultures, the fungus was inoculated in 5 Erlenmeyer flasks (200 mL per flask) for 17 (14.41 mg) and 30 days (26.14 mg) at 25 °C. The extracts were obtained in the form of yellow oils and three fractions were collected from column chromatography of surface cultures Fr1 (3.96 mg), Fr2 (0.3 mg) and Fr3 (3.45 mg) for 17 days and Fr1 (10.9 mg), Fr2 (1.81 mg) and Fr3 (6.43 mg) for 30 days. 2-phenylethan-1-ol (**19**) (1.5 mg) [39] and tyrosol (**20**) (2.43 mg) [39] were obtained from Fr1 after 17 d, and **17** (6.51 mg) [58] from Fr1 for 30 d. Fr2 and Fr3 from both extracts were composed of fatty acids.

#### 4.4.2. Czapek–Dox Medium Fermentation

*E. lata* 311 was incubated in 6 Roux bottles (150 mL per bottle) or in 5 Erlenmeyer flasks (200 mL per flask) for 17 days. Evaporation of the solvent at reduced pressure yielded extracts in the form of yellow oils, i.e., 171.65 mg (from surface culture) and 280.82 mg (from shaken cultures).

The chromatography of the surface fermentation extract yielded seven fractions: Fr1 (*n*-hexane) (1.92 mg), Fr2 (*n*-hexane-ethyl acetate, 8:2) (10.47 mg), Fr3 (*n*-hexane-ethyl acetate, 6:4) (28.38 mg), Fr4 (*n*-hexane-ethyl acetate, 4:6) (16.97 mg), Fr5 (*n*-hexane-ethyl acetate, 2:8) (6.45 mg), Fr6 (ethyl acetate) (8.32 mg) and Fr7 (EtOAc-MeOH, 9:1) (37.02 mg). Fr3 was identified as siccayne (**2**) [41], Fr4 was a mixture of 5-(3-methylbuta-l.3-dienylidene)-2,3-epoxycyclohexane-l,4-diol (**15**) [28] and **17** [40], Fr5 yielded compound **14** [28], Fr6 was purified by analytical HPLC (*n*-hexane:EtOAc:acetone 6.5:5:0.5, flow 0.8 mL/min) attaining compound **12** (0.6 mg) [42], and Fr7 resulted in the new compound **5**.

In the case of the shaken cultures, the same seven fractions were collected by column chromatography, i.e., Fr1 (*n*-hexane) (1.74 mg), Fr2 (*n*-hexane-ethyl acetate, 8:2) (6.99 mg), Fr3 (*n*-hexane-ethyl acetate, 6:4) (11.94 mg), Fr4 (*n*-hexane-ethyl acetate, 4:6) (9.29 mg), Fr5 (*n*-hexane-ethyl acetate, 2:8) (28 mg), Fr6 (ethyl acetate) (5.48 mg), and Fr7 (EtOAc-MeOH, 9:1) (76.62 mg). Fr3 yielded a compound thst was identified as **2** [41]. Fr4 was purified by HPLC, *n*-hexane:ethyl acetate:acetone (5:4:1) 0.8 mL/min flow, obtaining the known compound **17** (1.56 mg) [40]. Fr7 was submitted to column chromatography using an increasing gradient of chloroform to methanol as solvent and yielding twelve fractions. The combination of five of these fractions from Fr7 yielded compound **5** (32 mg).

Eulatagalactoside (**5**): purified through CC (MeOH:EtOAc 10:90) as a yellow oil; ${\left[\alpha \right]}_{D}^{21}$-27.7 (c 1.9, MeOH); IR ν_max_ (cm^−1^) 3405, 2962, 2928, 1660, 1429, 755 (Supporting Information, Figure S13); HRMS(ESI^+^) calculated for C_17_H_21_O_7_ [M+H]^+^ 337.1287, found 337.1264; calculated (Supporting Information, Figure S14); ^1^H NMR and ^13^C NMR, see Table 1.

(3*S*,4*R*)-6-(Hydroxymethyl)-2,2-dimethyl-3,4-dihydro-2*H*-chromene-3,4-diol (**12**): purified through analytical HPLC (*n*-hexane:EtOAc:acetone 6.5:5:0.5, flow 0.8 mL/min, *t*_R_ = 38.21 min. as a colourless oil. ${\left[\alpha \right]}_{D}^{21}$+10.7 (c 0.051, CHCl_3_); ECD (MeOH) *λ* (Δ*ε*) 214 (−10.23), 227 (4.19), 245 (−0.51) nm.

(6*R*,7*S*,8*S*)-6-Hydroxy-2,2-dimethyl-5,6,7,8-tetrahydro-7,8-epoxychroman (**14**) [28]: ${\left[\alpha \right]}_{D}^{21}$-17.4 (c 0.07, MeOH); ECD (MeOH) *λ* (Δ*ε*) 320 (−11.04), 212 (−3.18), 233 (2.01) nm.

### 4.5. Solid Culture Fermentation

#### 4.5.1. PDA Medium Fermentation

The *E. lata* 311 strain was fermented in 1 L of PDA medium (100 mL per petri dish) for 20 days at 25 °C under white light and darkness. Extracts of 56.23 mg and 102.31 mg, respectively, were chromatographed in six or seven fractions:

Under white light: Fr1 (*n*-hexane) (1.19 mg), Fr2 (*n*-hexane-ethyl acetate, 7:3) (4.25 mg), Fr3 (*n*-hexane-ethyl acetate, 6:4) (5.26 mg), Fr4 (*n*-hexane-ethyl acetate, 5:5) (2.04 mg), Fr5 (ethyl acetate) (3.34 mg), and Fr6 (EtOAc-MeOH, 9:1) (8.61 mg). Compounds **1** (1.02 mg) [29], **4** (5.26 mg) [28], **14** (1.2 mg) [28], and **5** (8.6 mg) were identified from Fr2, Fr3, Fr4 and Fr6.

In darkness: Fr1 (*n*-hexane) (2.71 mg), Fr2 (*n*-hexane-ethyl acetate, 8:2) (5.53 mg), Fr3 (*n*-hexane-ethyl acetate, 6:4) (9.86 mg), Fr4 (*n*-hexane-ethyl acetate, 4:6) (12.72 mg), Fr5 (*n*-hexane-ethyl acetate, 2:8) (3.48 mg), Fr6 (ethyl acetate) (22.55 mg) and Fr7 (EtOAc-MeOH, 9:1) (22.27 mg). Fr2, Fr3, Fr4, and Fr7 contained the compounds **1** (1.34 mg) [29], **4** (9.86 mg) [29], **15** (12.45 mg) (${\left[\alpha \right]}_{D}^{26}$-10.10° (c 2, MeOH)) [28] and **5** (22 mg).

#### 4.5.2. AM Medium Fermentation

The *E. lata* 311 strain was fermented in 1 L of AM medium (100 mL per petri dish) for 20 days at 25 °C under white light. The extract (178.5 mg) was chromatographed in seven fractions: Fr1 (*n*-hexane) (1.05 mg), Fr2 (*n*-hexane-ethyl acetate, 8:2) (24.38 mg), Fr3 (*n*-hexane-ethyl acetate, 6:4) (7.75 mg), Fr4 (*n*-hexane-ethyl acetate, 4:6) (8.82 mg), Fr5 (*n*-hexane-ethyl acetate, 2:8) (8.62 mg), Fr6 (ethyl acetate) (8.35 mg) and Fr7 (EtOAc-MeOH, 9:1) (10 mg). Fr3, Fr4 and Fr7 contained the compounds **4** (6.46 mg) [29], **14** (0.87 mg) [28] and **15** (8.82 mg) [28].

#### 4.5.3. Rice Medium

*E. lata* was inoculated in 20 Erlenmeyer flasks containing rice medium. The extract (3560 mg) was chromatographed on CC obtaining the following fractions: Fr1 (*n*-hexane) (12.66 mg), Fr2 (*n*-hexane-ethyl acetate, 7:3) (223.6 mg), Fr3 (*n*-hexane-ethyl acetate, 6:4) (68.96 mg), Fr4 (*n*-hexane-ethyl acetate, 5:5) (98.93 mg), Fr5 (*n*-hexane-ethyl acetate, 4:6) (148.56 mg), Fr6 (*n*-hexane-ethyl acetate, 3:7) (63.33 mg), Fr7 (*n*-hexane-ethyl acetate, 2:8) (95.39 mg), Fr8 (ethyl acetate 1:9) (65.13 mg), and Fr9 (EtOAc-MeOH, 9:1) (152.15 mg).

Fr5 and Fr6 were purified by semipreparative HPLC with eluent *n*-hexane:ethyl acetate:acetone (4.5:5:0.5), 2.5 mL/min flow, obtaining the new analogue (*S*)-7-(hydroxymethyl)-3-methyl-2,3-dihydrobenzo[*b*]oxepin-3-ol (**11**) (5.56 mg) and the known ones, **3** (1 mg) (Fr4, *t*_R_ = 52.92 min) [34], **4** (7.40 mg) (Fr4, *t*_R_ = 46.84 min) [29] and **6** (2.3 mg) (Fr5, *t*_R_ = 13.93 min) [29]. Fr7 and Fr8 were submitted to further purification by HPLC using *n*-hexane:ethyl acetate:acetone (5.5:4:0.5) 2.5 mL/min flow, obtaining the phenol **8** [45] and **10** (1 mg) [47]. Peaks 13 (*t*_R_ = 48.53 min) and 15 (*t*_R_ = 62.07 min) from Fr7 were also purified using an analytical column and the eluent previously described by Fr7, obtaining the new derivatives, i.e., (*R*)-2-(4-hydroxy-3-methylbut-1-yn-1-yl)-4-(hydroxymethyl)phenol (**7**) and (3a*R*,4*S*,5*R*,7a*S*)-4,5-dihydroxy-6-((*R*)-3′-methylbuta-1′,3′-dien-1′-ylidene)hexahydrobenzo[*d*][1,3]dioxol-2-one (**16**) and the known ones **10** (1.3 mg) [47], **13** (1 mg) [48], and **9** (0.67 mg) [50].

(*R*)-2-(1-hydroxy-3-methylbut-3-en-1-yl)benzene-1,4-diol (**7**): purified through analytical HPLC (*n*-hexane:EtOAc:acetone 5.5:4:0.5, flow 0.8 mL/min, *t*_R_ = 28.36 min (Fr6_Peak 15)) as a colourless oil. ${\left[\alpha \right]}_{D}^{21}$-1.07 (c 0.25, CHCl_3_); IR ν_max_ (cm^−1^) 3345, 2926, 1609, 1496, 1035, 771 (Supporting Information, Figure S22); ECD (MeOH) *λ* (Δ*ε*) 259 (−2.43), 288 (−2.88), 308 (4.87) nm; HRMS(ESI^+^) calculated for C_12_H_15_O_3_ [M+H]^+^ 207.1021, found 207.1027 (Supporting Information, Figure S23); ^1^H NMR and ^13^C NMR, see Table 2.

(*R*)-4-hydroxy-3-(1-hydroxy-3-methylbut-3-en-1-yl)benzoic acid (**8**) [45]. ${\left[\alpha \right]}_{D}^{21}$-6.4 (c 0.17, CHCl_3_); ECD (MeOH) *λ* (Δ*ε*) 280 (−35.30), 297 (14.72), 305 (12.41), 344 (−9.85) nm.

(*S*)-7-hydroxymethyl-3-methyl-2,3-dihydro-1-benzoxepin-3-ol (**11**): purified through analytical HPLC (*n*-hexane:EtOAc:Acetone 4.5:5:0.5, flow 0.8 mL/min, *t*_R_ = 65.52 min (Fr5)) as a colourless oil. ${\left[\alpha \right]}_{D}^{21}$+89.5 (c 0.5, CHCl_3_); IR ν_max_ (cm^−1^) 3367, 2926, 1795, 1499, 1225, 1122, 1036, 904, 829 (Supporting Information, Figure S34); ECD (MeOH) *λ* (Δ*ε*) 281 (2.16), 304 (−3.42), 327 (−1.55) nm; HRMS(ESI^+^) calculated for C_12_H_15_O_3_ [M+H]^+^ 207.1021, found 207.1026 (Supporting Information, Figure S35); ^1^H NMR and ^13^C NMR, see Table 3.

(3a*R*,4*S*,5*R*,7a*S*)-4,5-Dihydroxy-6-((*R*)-3′-methylbuta-1′,3′-dien-1′-ylidene)hexahydrobenzo[*d*][1,3]dioxol-2-one (**16**): purified through analytical HPLC (*n*-hexane:EtOAc:acetone 5.5:4:0.5, flow 0.8 mL/min, t_R_ = 20.71 min (Fr6_Peak 13)) as a colourless oil. ${\left[\alpha \right]}_{D}^{21}$+5.21 (c 0.27, CHCl_3_); IR ν_max_ (cm^−1^) 3405, 2956, 2923, 1798, 1044; ECD (MeOH) *λ* (Δ*ε*) 234 (−2.37), 252 (6.62), 266 (−3.23), 295 (8.28) nm; HRMS(ESI^+^) calculated for C_12_H_13_O_5_ [M-H]^+^ 237.0763, found 237.0760; ^1^H NMR and ^13^C NMR, see Table 4.

### 4.6. Computational Details of EDC Calculations

The molecular structure analysis of compounds **7**, **8**, **10**, **11**, **12**, **14**, and **16** employed the semiempirical PM6 method [78]. Quantum mechanical computations were carried out using the Gaussian 16 package [79]. The theoretical curves were calculated based on the more stable configuration. In this study, we performed a conformational search to identify the most stable conformers prior to ECD spectral calculations. Comprehensive geometric optimization was conducted with density functional theory (DFT) employing the B3LYP functional and the 6–311+G(2d,p) basis set, allowing for an exploration of the potential energy surface and the identification of low-energy conformations relevant for ECD prediction [80,81].

Following this, calculations were executed to determine energies, oscillator strengths, and rotational strengths for the initial 20 electronic excitations employing the TD-DFT methodology [44,82]. The solvent effect (methanol) was considered in the calculations using the polarizable continuum model (PCM) with the implicit solvation energy (IEF) approach [83,84,85,86]. To replicate the ECD spectrum of the conformer, a Gaussian function was utilized with a half-bandwidth of 0.33 eV.

### 4.7. In Vitro Phytotoxic Assays

The assays were performed in *Phaseolus vulgaris* plants following a protocol previously described [87]. Attached leaves were sterilized with an aqueous solution of 10% ethanol (*v*/*v*), washed with sterile water, and dried in filter paper. Two *Phaseolus* plants were used per experiment. Five leaves per plant were treated with a solution of the compound to be tested. Each leaf received five drops (10 µL) of the control solution and five drops (10 µL) of the test solution consistent in a solution of the compounds dissolved in a mixture of acetone–distilled water (1% tween 80) (10:90 *v*/*v*). Two experiments were carried out for each compound and concentration. The plants were incubated at 25–28 °C in natural sunlight for a minimum photoperiod of 12 h. The diameter of the drops was identical in each case, and the total surface area of the drops was considered the treated area.

### 4.8. In Vitro Antimicrobial Assays

The antimicrobial activities of compounds **2**, **4**, **5**, **7**, **8**, **11**, **13**, and **16** were evaluated against *Escherichia coli* CECT434, *Klebsiella pneumoniae* CECT7787, and *Staphylococcus aureus* CECT794. Assays were conducted through the broth microdilution method according to CLSI protocols with some modifications [88]. In brief, bacterial cultures were grown overnight and the bacterial concentration was adjusted to a 0.5 McFarland standard, corresponding to 1.5 × 10^8^ CFU/mL. Each compound was dissolved in dimethylsulfoxide (DMSO), ensuring the solvent did not exceed 4% of the total well volume. For the assay, 10 μL/well of the inoculum was mixed with 4 μL/well of compound dissolved in DMSO and 86 μL/well of Mueller–Hinton (MH) broth. Chloranphenicol (32 mg/mL) was used as the positive control for Gram-positive and Gram-negative bacteria. Additionally, untreated bacteria were included as a negative control and blanks were prepared with MH broth and DMSO. The plates were incubated in a Multiskan GO plate reader (ThermoFisher, Waltham, MA, USA) at 37 °C under aerobic conditions and shaken for 24 h. The plate reader was programmed to take absorbance measurements every hour at a wavelength of 600 nm. Measurements were conducted in triplicate, and the average values were used to determine the percentage inhibition.

The percentage inhibition (PI) of bacterial growth was determined using the method described by Zhang et al. [89]. The PI did not reach the standard MIC threshold of 90%. Therefore, IC_50_ values were calculated using a nonlinear dose–response regression to provide a more accurate representation of the compounds’ efficacy. The maximum inhibition percentages at the highest concentrations tested are also reported.

## 5. Conclusions

The OSMAC approach has proven to be a promising strategy for the study of the secondary metabolism of phytopathogenic fungus *E. lata* 311. The isolation of eutypine (**1**) and derivatives showed the phytotoxicity of this strain, and with the new metabolites identified, the potential of the enzymatic complex of this strain.

Four new metabolites were identified for the extracts of various media and culture conditions, rice medium being the best to isolate new compounds. In addition to this, five metabolites were isolated from *E. lata* for the first time.

New metabolites were tested and found to be phytotoxic and antimicrobial against three bacteria. Only one of the new metabolites, i.e., number **5**, showed phytotoxicity, while the other metabolites showed moderate antimicrobial activity against Gram-positive bacteria in comparison to siccayne (**2**), which was described to have antimicrobial activity.

Future research will involve the study of the genome of strain *E. lata 311*. Blanco-Ullate et al. [89] carried out a study with a draft genome sequence of *E. lata* UCR-EL1, and an informatic process identified that among the putative secreted proteome, homology with enzymes enhanced the breakdown of lignocellulosic material in combination with cellulolytic and hemicellulolytic enzymes and a large number of putative cytochromes P450 monooxygenases. This information is in line with the internal development of this fungus, but does not provide information about the genes involved in the synthesis of toxic metabolites. Therefore, the sequencing of the total genome of *E. lata* strain 311 could provide relevant information about the families of genes involved in the most important biosynthetic pathways and discover possible new pathways as targets for the activation of silent genes to isolate new metabolites.

## Figures and Tables

**Table 1:** ^1^H and ^13^C NMR spectroscopic data for new compound **5**.

Position	*δ*_H_, Mult (*J* in Hz) ^a^	*δ*_C_, Type ^b^
1	-	152.2, C
2	-	115.9, C
3	6.75, d (3), 1H	C 119.7, CH
4	-	153.4, C
5	6.71, dd (8.9, 3.0), 1H	117.6, CH
6	7.06, d (8.9), 1H	119.5, CH
1′	-	85.8, C
2′	-	95.2, C
3′	-	128.5, C
4′a	5.37, dq (2.1, 1.0), 1H	122.2, CH_2_
4′b	5.28, m, 1H	
5′	1.97, dd (1.6, 1.0), 3H	23.6, CH_3_
1″	4.81, d (7.7), 1H	103.7, C
2″	3.81, dd (9.7, 7.7), 1H	72.4, CH
3″	3.56, dd (9.7, 3.4), 1H	74.9, CH
4″	3.87, dd (3.4, 1.1), 1H	70.1, CH
5″	3.60, ddd (6.7, 5.5, 1.1), 1H	76.9, CH
6″	3.74, m, 2H	62.3, CH_2_

^a^ 400 MHz, CD_3_OD; ^b^ 100 MHz, CD_3_OD.

**Table 2:** ^1^H NMR and ^13^C NMR spectroscopic data for new compound **7**.

Position	*δ*_H_, Mult (*J* in Hz) ^a^	*δ*_C_, Type ^a^
1		156.5, C
2		109.6, C
3	7.31, d (br), 1H	130.1, CH
4		132.6, C
5	7.21, dd (8.3, 2.1), 1H	129.1, CH
6	6.91, d (8.3), 1H	114.6, CH
7	4.57, s, 2H	64.7, CH_2_
1′		76.5, C
2′		99.1, C
3′	2.96, m, 1H	30.1, CH
4′a 4′b	3.73, dd (10.3, 5.5), 1H 3.64, dd (10.3, 7.2), 1H	66.7, CH_2_
5′	1.29, d (7.0), 3H	16.9, CH_3_

^a^ 700 MHz, CDCl_3_.

**Table 3:** ^1^H NMR and ^13^C NMR spectroscopic data for new compound **11**.

Position	*δ*_H_, Mult (*J* in Hz) ^a^	*δ*_C_, Type ^a^
2α 2β	4.12, dd (11.9, 1.8), 1H 3.87, d (11.9), 1H	78.5, CH_2_
3		72.6, C
4	5.93, dd (11.9, 1.7), 1H	136.5, CH
5	6.25, dd (11.9, 0.6), 1H	126.5, CH
5a		135.6, C
6	7.19, d (br) (2.3), 1H	131.5, CH
7		126.3, C
8	7.20, dd (8.0, 2.3), 1H	
9	7.02, d (8.0), 1H	120.2, C
9a		158.4, C
10	1.33, s, 3H	24.1, CH_3_
11	4.63, s, 2H	64.8, CH_2_

^a^ 700 MHz, CDCl_3._.

**Table 4:** ^1^H NMR and ^13^C NMR spectroscopic data for new compound **16**.

Position	*δ*_H_, Mult (*J* in Hz) ^a^	*δ*_C_, Type ^a^
2		154.0, C
3a	4.62, td (7.0, 0.6), 1H	79.6, CH
4	3.82, dd, (9.0, 7.0), 1H	75.9, CH
5	4.04, dd (9.0, 3.4), 1H	69.7, CH
6		100.0, C
7α 7β	2.94, dd, (16, 3.3), 1H 2.66 m, 1H.	29.2, CH_2_
7a	4.90, ddd (7.0, 4.5, 3.3), 1H	75.4, CH
1′		200.8, C
2′	6.30, t (3.5), 1H	103.9, CH
3′		137.6, C
4′a 4′b	5.04, dq (1.6, 0.8), 1H 4.96, dq (1.6, 1.5), 1H	116.5, CH_2_
5′	1.75, dd (1.5, 0.8), 3H	19.6, CH_3_

^a^ 700 MHz, CDCl_3_.

## Data Availability

The original contributions presented in this study are included in the article/supplementary material. Further inquiries can be directed to the corresponding author(s).

## Supplementary Materials

The following supporting information can be downloaded at https://www.mdpi.com/article/10.3390/ijms26125774/s1.
