# Supplementary material for: Discovery of Novel Phenolic Compounds from Eutypa lata Through OSMAC Approach: Structural Elucidation and Antibiotic Potential
Source: Int J Mol Sci. 2025 Jun 16;26(12):5774. doi: 10.3390/ijms26125774 (PMC12193298; doi:10.3390/ijms26125774)
Supplement: Supplementary file 1 [file ijms-26-05774-s001.zip › ijms-3490419-supplementary.pdf]

Discovery of Novel Phenolic Compounds from *Eutypa lata* through OSMAC Aproach: Structural Elucidation and Antibiotic Potential

Ana Cotán, Inmaculada Izquierdo-Bueno, Abdellah Ezzanah, Laura Martín, Manuel Delgado, Isidro G. Collado and Cristina Pinedo-Rivilla

Supporting Information

S1.- ^{1}H of Eutypine (**1**) in $CDCl_3$ 3

S2.- ^{1}H of Siccayne (**2**)....... 3

S3.- ^{13}C of Siccayne (**2**) in $CDCl_3$....... 4

S4.- ^{1}H of Eulatinol (**3**) in $CDCl_3$**.** 4

S5.- ^{13}C of Eulatinol (**3**) in $CDCl_3$**.** 5

S6.- ^{1}H of Eutypinol (**4**) in CD_3OD. 5

S7.- ^{13}C of Eutypinol (**4**) in CD_3OD. 6

S8.- ^{1}H of **5** in CD_3OD**.** 6

S9.- ^{13}C of **5** in CD_3OD**.** 7

S10.- NOESY of **5**....... 7

S11.- gCOSY of **5**. 8

S12.- gHMBC of **5**. 8

S13.- MS of **5**. 9

S14.- IR spectrum of **5**. 9

S15.- ^{1}H of Eutypinic acid (**6**) in $CDCl_3$. 10

S16.- ^{13}C of Eutypinic acid (**6**) in $CDCl_3$. 10

S17.- ^{1}H of **7** in $CDCl_3$. 11

S18.- ^{13}C of **7** in $CDCl_3$. 11

S19.- gCOSY of **7** in $CDCl_3$. 12

S20.- DEPT of **7** in $CDCl_3$. 12

S21.- gHMBC of **7** in $CDCl_3$....... 13

S22.- MS of **7**. 14

S23.- IR spectrum of **7**. 14

S24.- ^{1}H of **8** in $CDCl_3$ 15

S25.- ^{13}C of **8** in $CDCl_3$ 18

S26.- ^{1}H of **9** in $CDCl_3$ 16

S27.- ^{13}C of **9** in $CDCl_3$ 16

S28.- ^{1}H of **10** in $CDCl_3$ 16

S29.- ^{13}C of **10** in $CDCl_3$ 16

S30.- ^{1}H of **11** in $CDCl_3$ 16

S31.- ^{13}C of **11** in $CDCl_3$. 18

S32.- gHSQC of **11** in $CDCl_3$ 16

S33.- gHMBC of **11** in $CDCl_3$. 19

S34.- MS of **11** in $CDCl_3$ 20

S35.- IR spectra of **11** 20

S36.- Comparison of ECD spectra of **10** and **11** 21

S37.- ^{1}H of **12** in $CDCl_3$ 21

S38.- ^{13}C of **12** in $CDCl_3$ 22

S39.- ^{1}H of **13** in $CDCl_3$ 22

S40.- ^{13}C of **13** in $CDCl_3$ 23

S41.- ^{1}H of **14** in $CDCl_3$ 23

S42.- ^{13}C of **14** in $CDCl_3$. 24

S43.- ^{1}H of **15** in CD_3OD. 24

S44.- ^{13}C of **15** in CD_3OD. 25

S45.- NOESY of **15** in CD_3OD. 25

S46.- ^{1}H of **16** in $CDCl_3$. 26

S47.- ^{13}C of **16** in $CDCl_3$. 26

S48.- gCOSY of **16** in $CDCl_3$. 27

S49.- DEPTof **16** in $CDCl_3$ 27

S50.- gHMBC of **16** in $CDCl_3$ 28

S51.- MS of **16** in $CDCl_3$ 28

S52.- IR spectrum of **16**. 29

S53.- Selected NOEs spectra of **16** 30

S54.- NOESY of **16**. 32

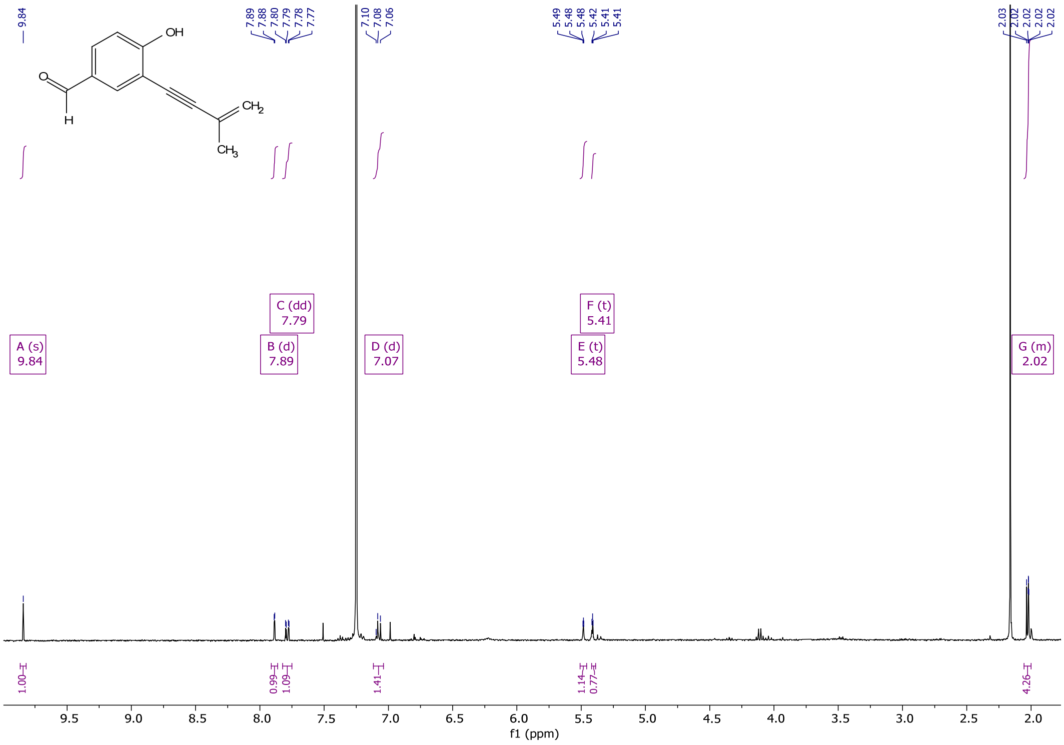

S1.- ^{1}H of Eutypine (1) in $CDCl_3$.

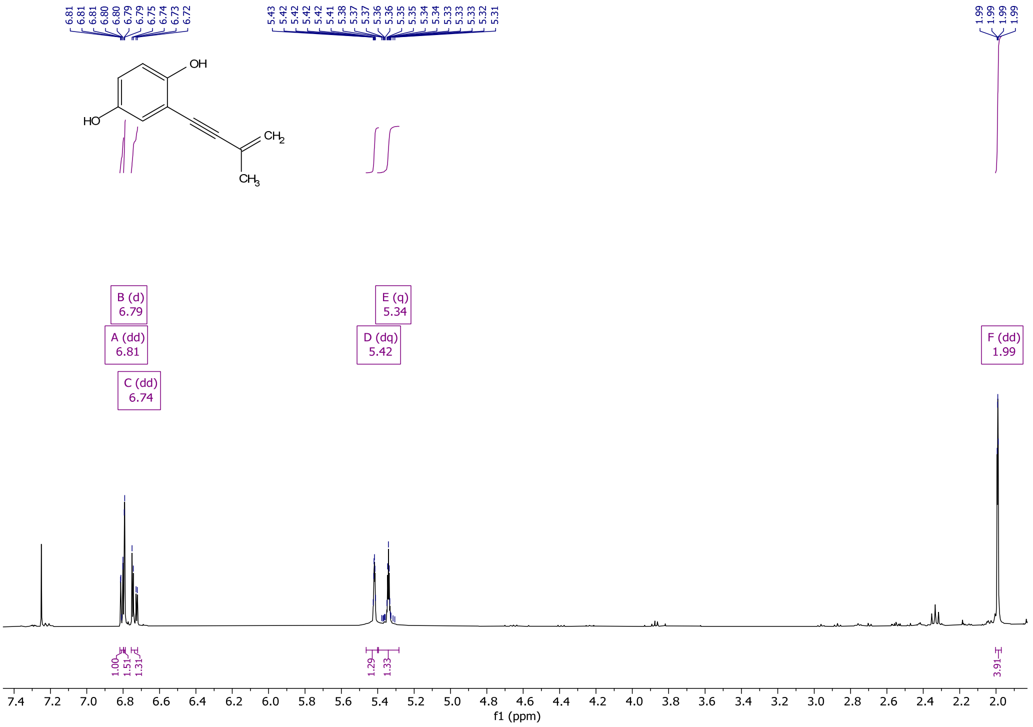

S2.- ^{1}H of Siccayne (2).

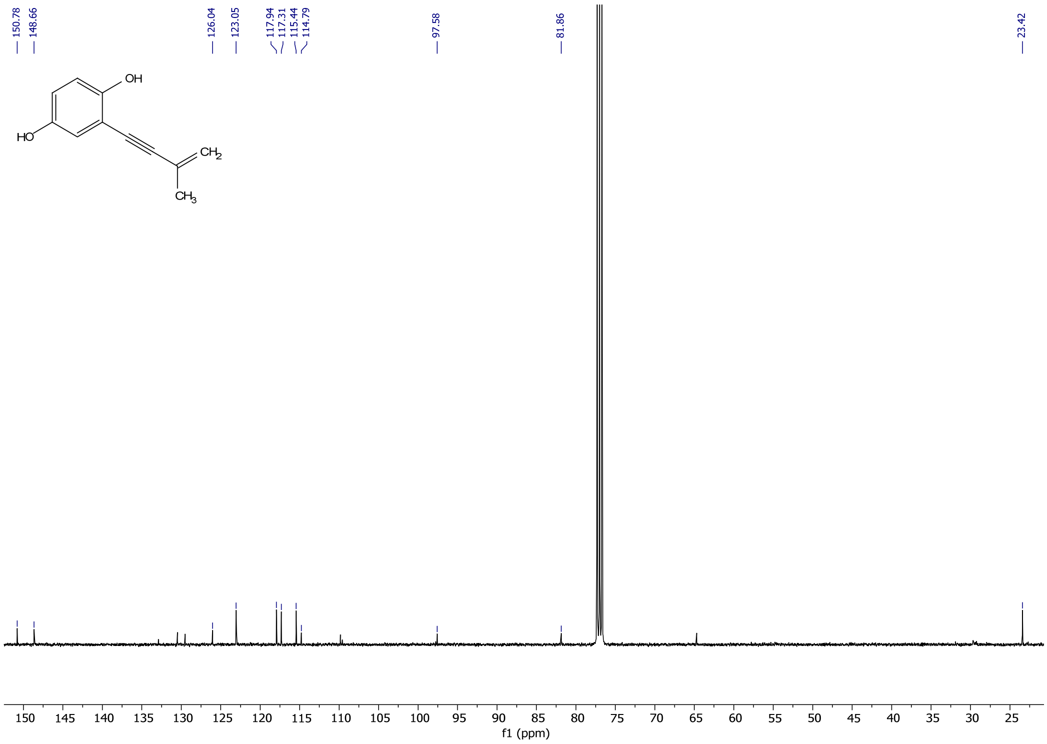

S3.- ^{13}C of Siccayne (2) in $CDCl_3$.

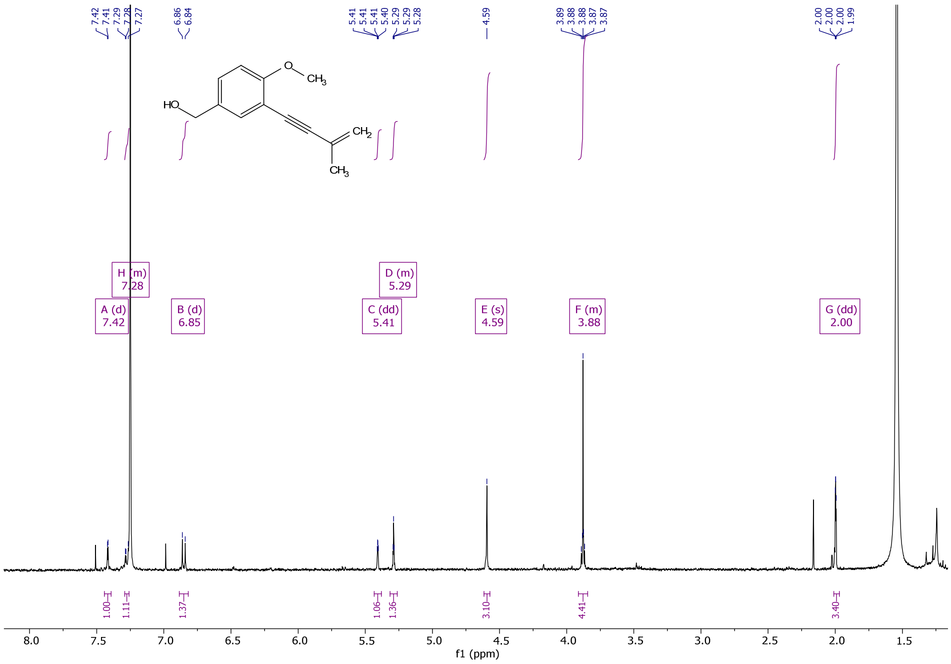

S4.- ^{1}H of Eulatinol (3) in $CDCl_3$.

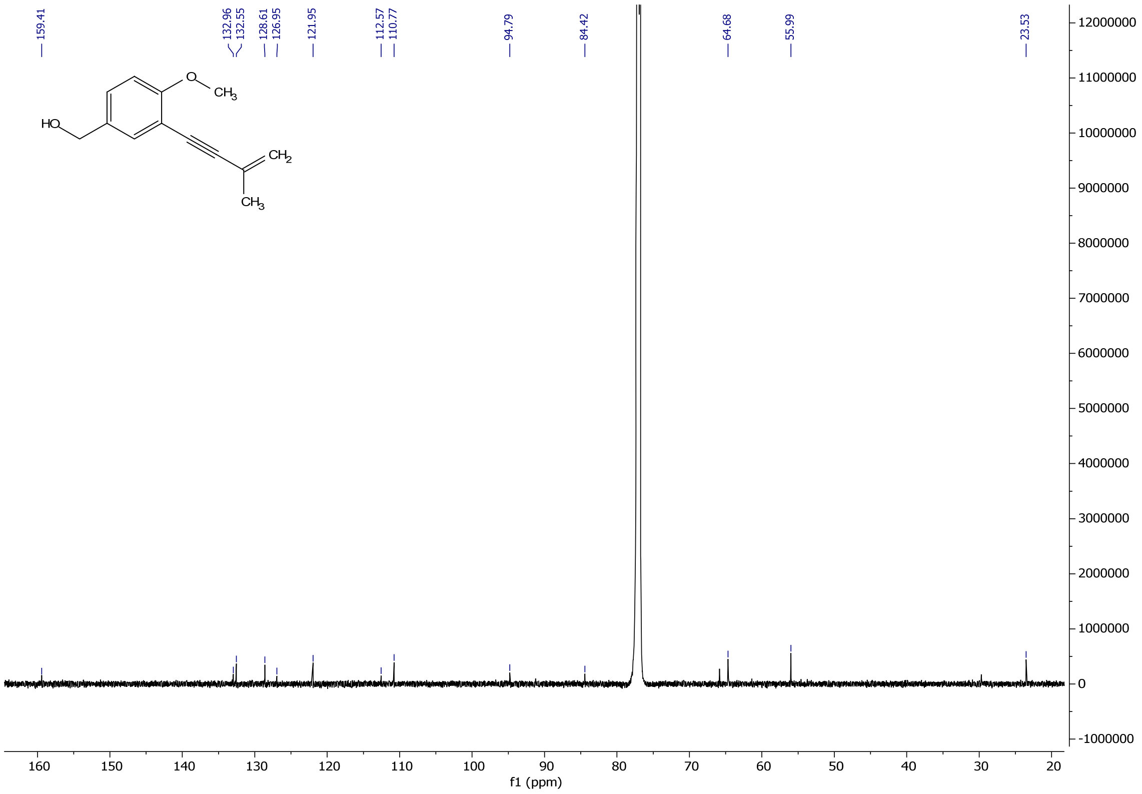

S5.- ^{13}C of Eulatinol (3) in $CDCl_3$.

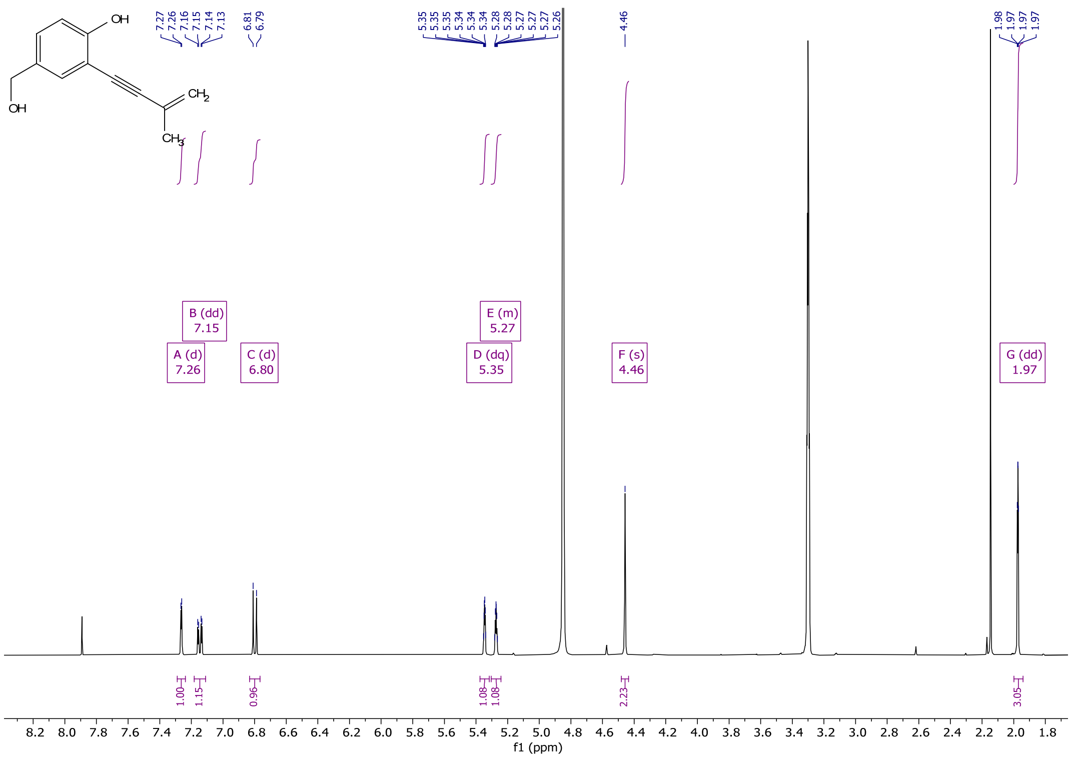

S6.- ^{1}H of Eutypinol (4) in CD_3OD.

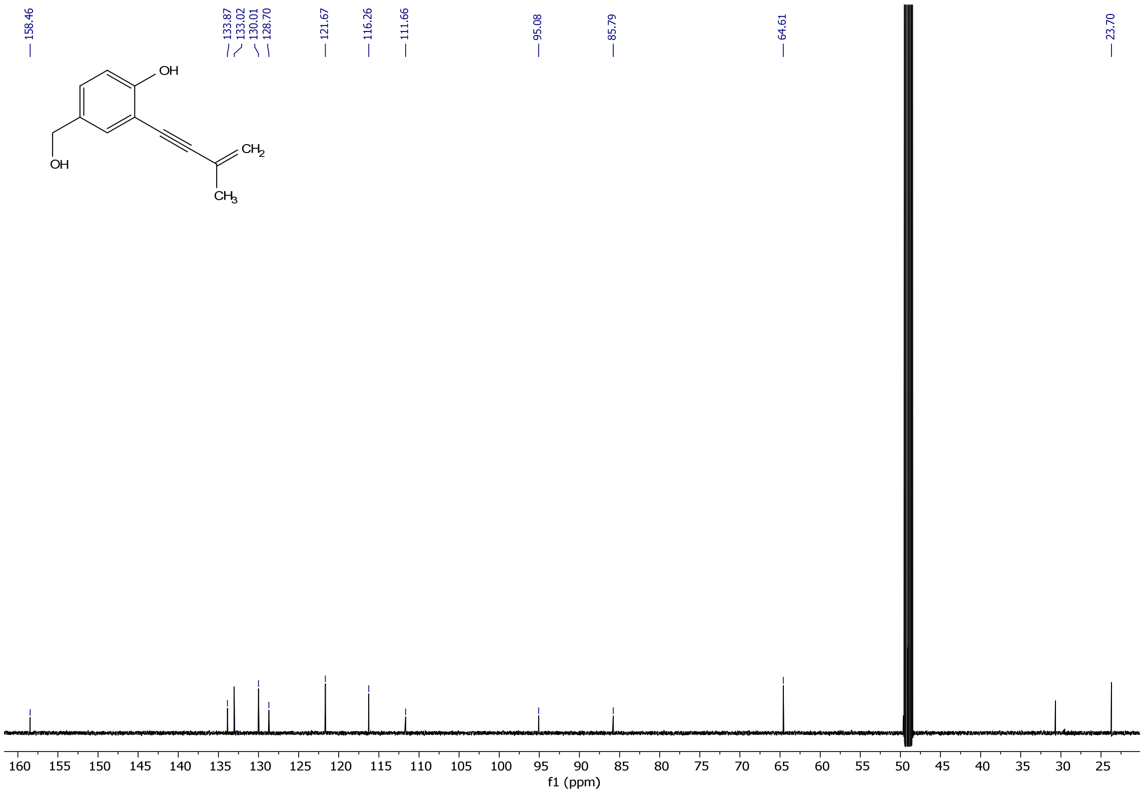

S7.- [13]C of Eutypinol (**4**) in CD_3OD.

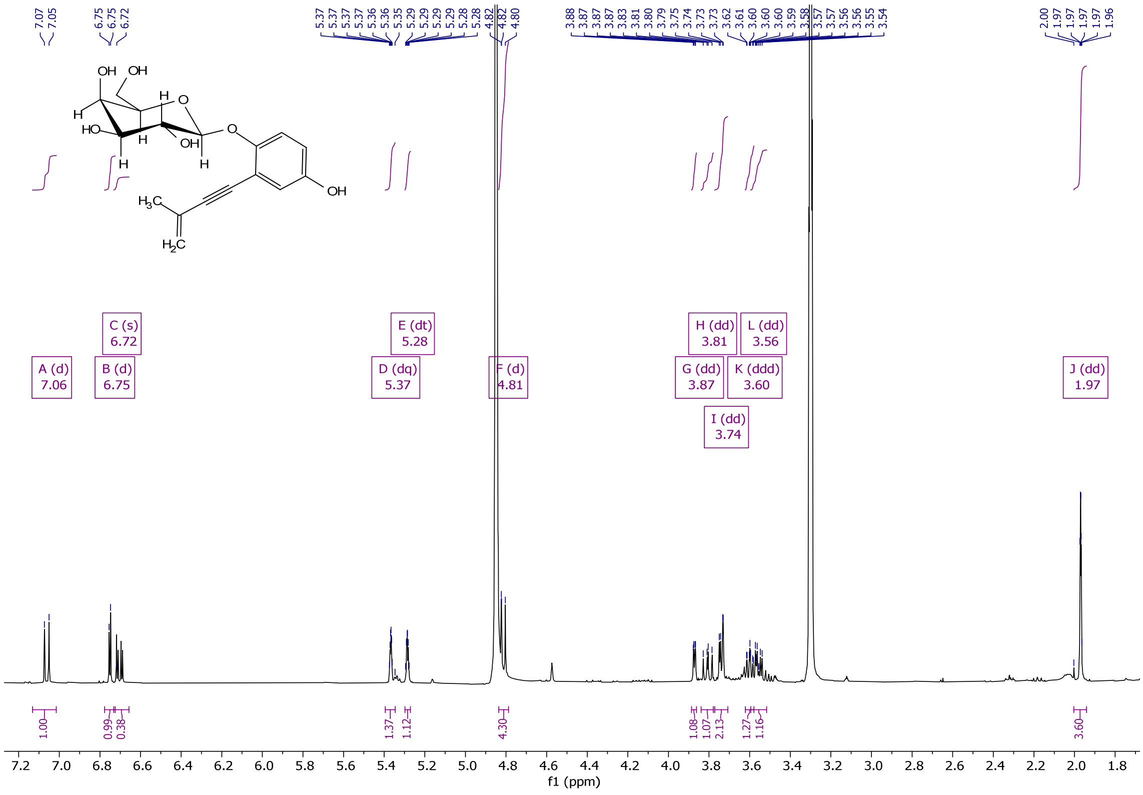

S8.- [1]H of **5** in CD_3OD.

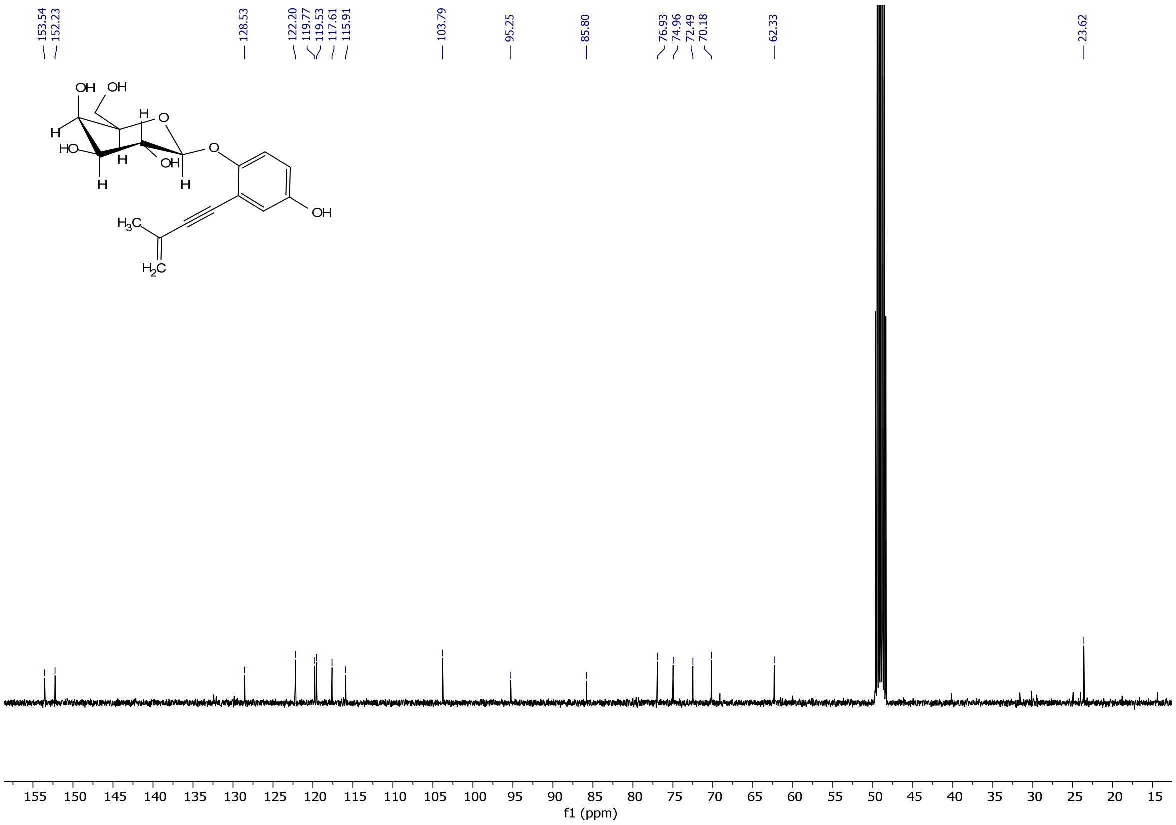

S9.- ^{13}C of **5** in CD_3OD.

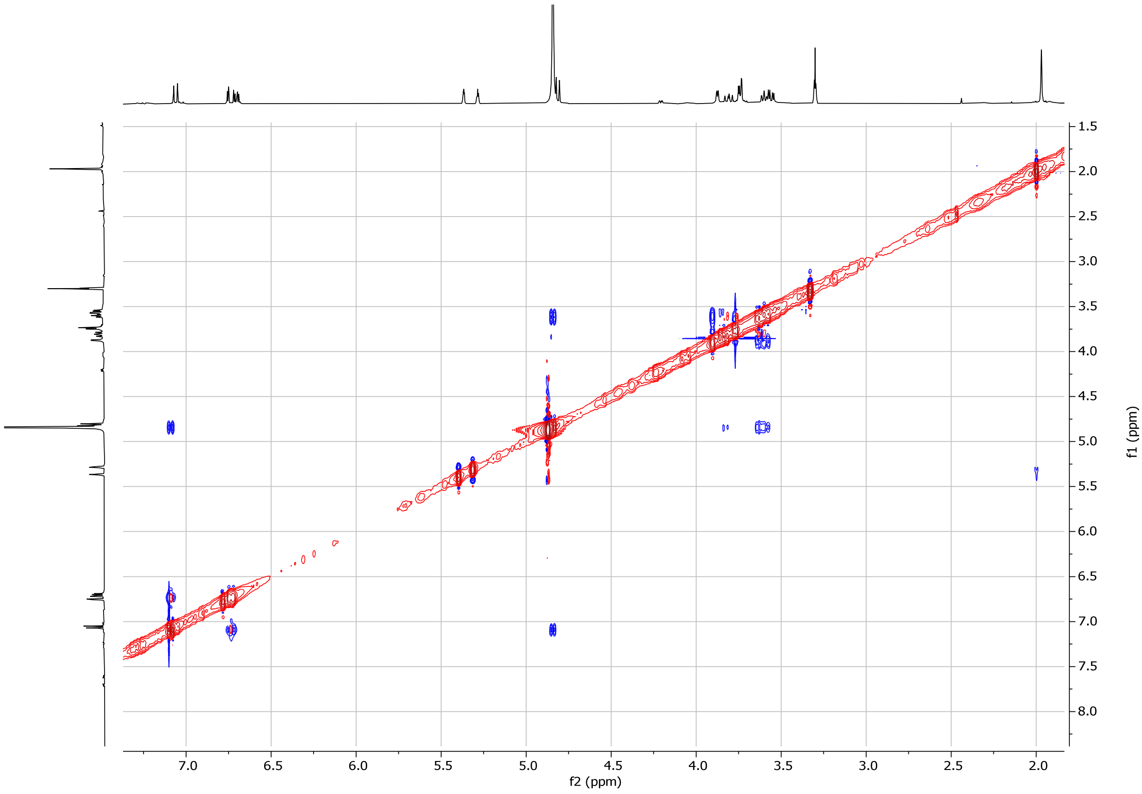

S10.- NOESY of **5**.

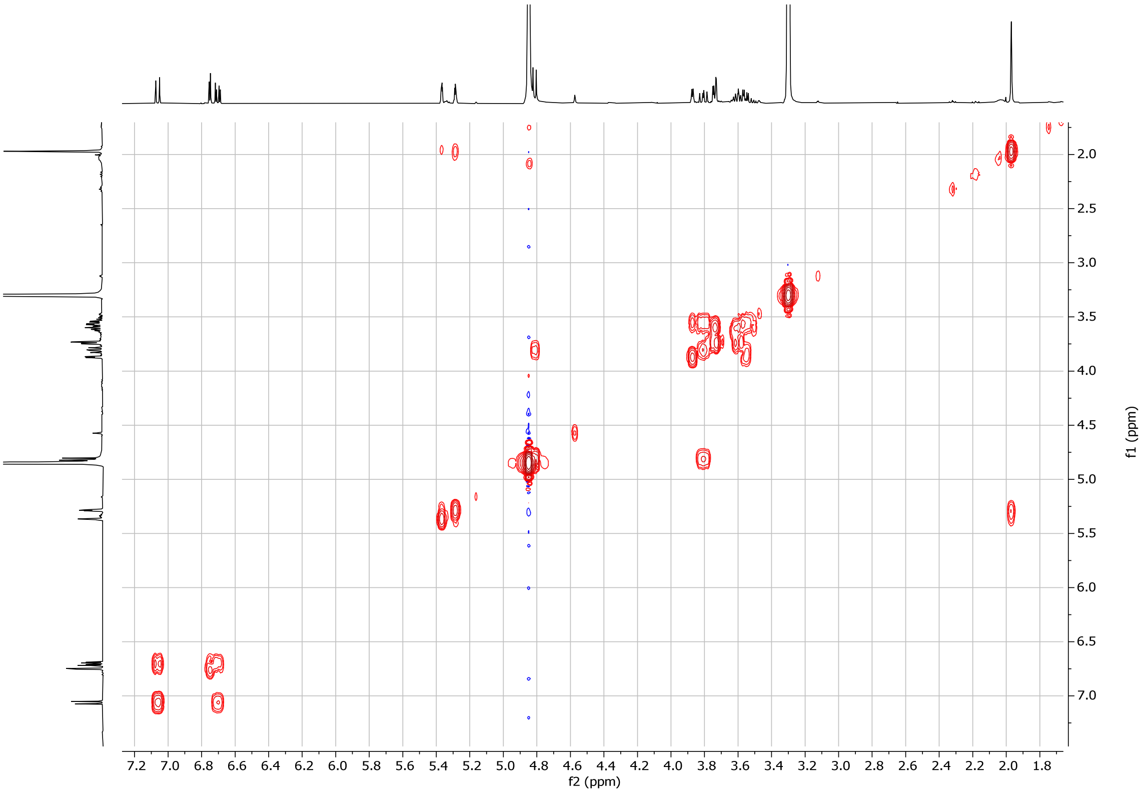

S11.- gCOSY of 5.

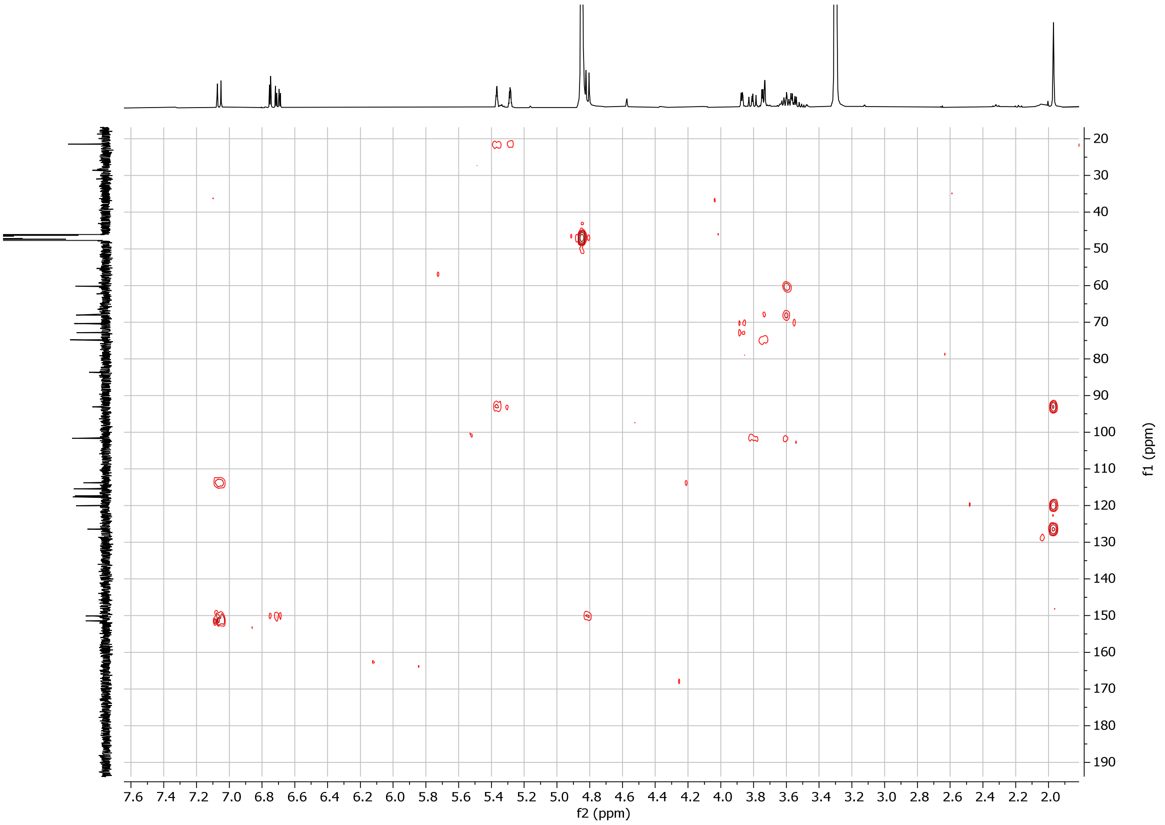

S12.- gHMBC of 5.

Elemental Composition Report **Page 1**

Single Mass Analysis
Tolerance = 3.0 mDa / DBE: min = -1.5, max = 80.0
Element prediction: Off
Number of isotope peaks used for i-FIT = 3

Monoisotopic Mass, Even Electron Ions
65 formula(e) evaluated with 1 results within limits (up to 5 best isotopic matches for each mass)
Elements Used:
C: 0-500 H: 0-1000 O: 0-50
EL_355_SOLIDO_10Me_7 34 (0.328)
1: TOF MS ES+

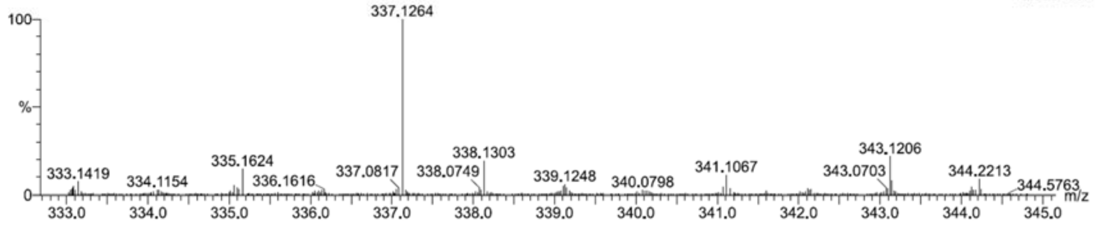

Minimum:				-1.5				
Maximum:		3.0	10.0	80.0				

Mass	Calc. Mass	mDa	PPM	DBE	i-FIT	Norm	Conf(%)	Formula
337.1264	337.1287	-2.3	-6.8	7.5	1053.5	n/a	n/a	C17 H21 O7

S13.- MS of 5.

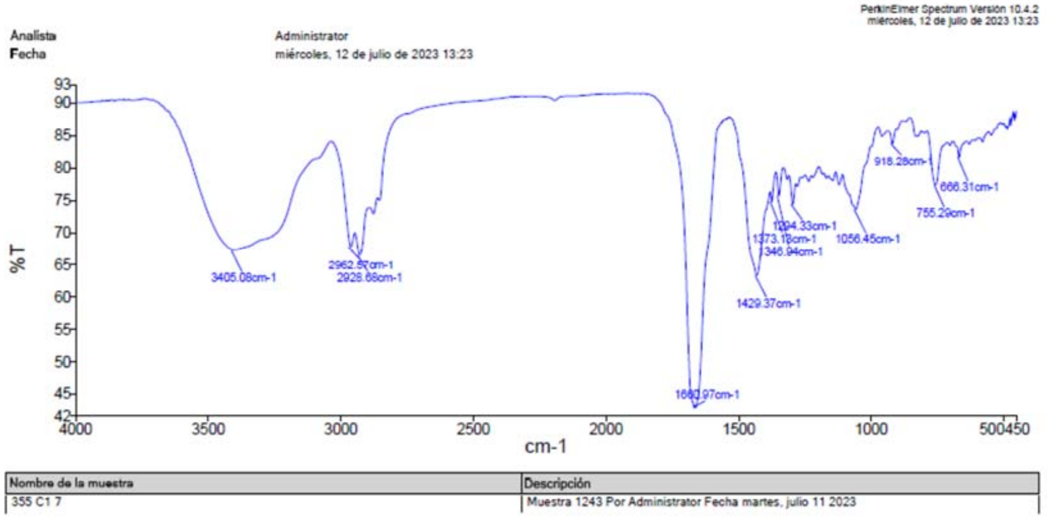

S14.- IR spectrum of 5.

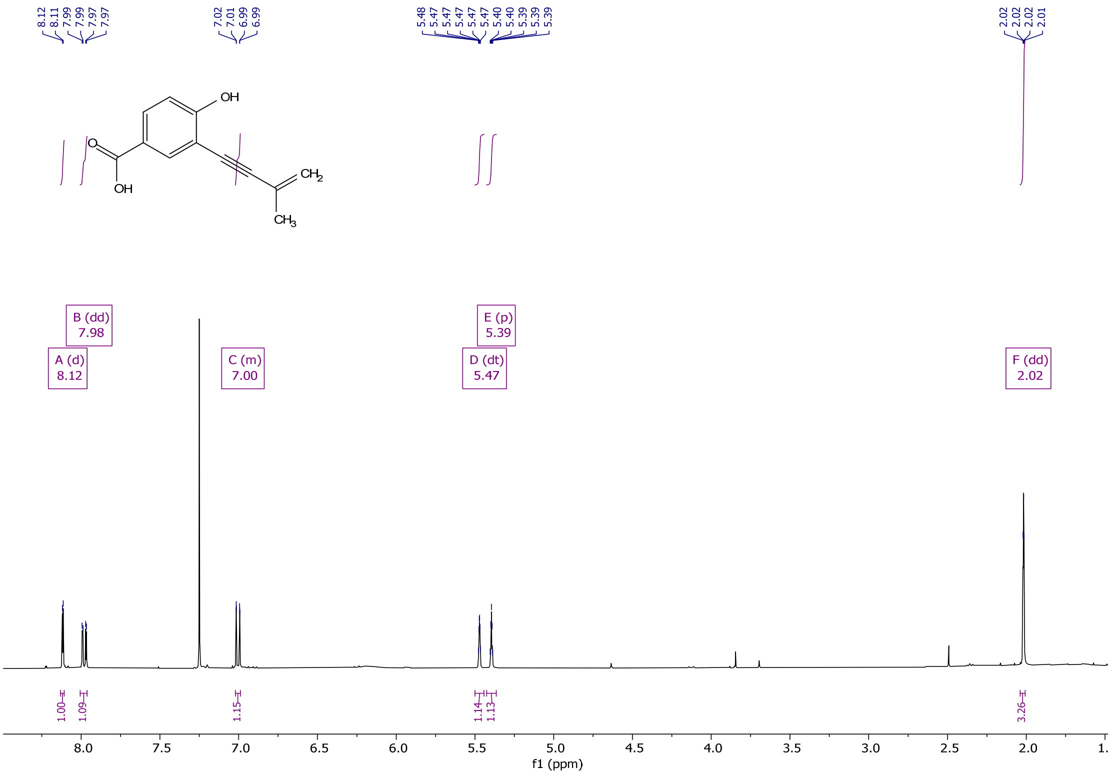

S15.- [1]H of Eutypinic acid (6) in $CDCl_3$.

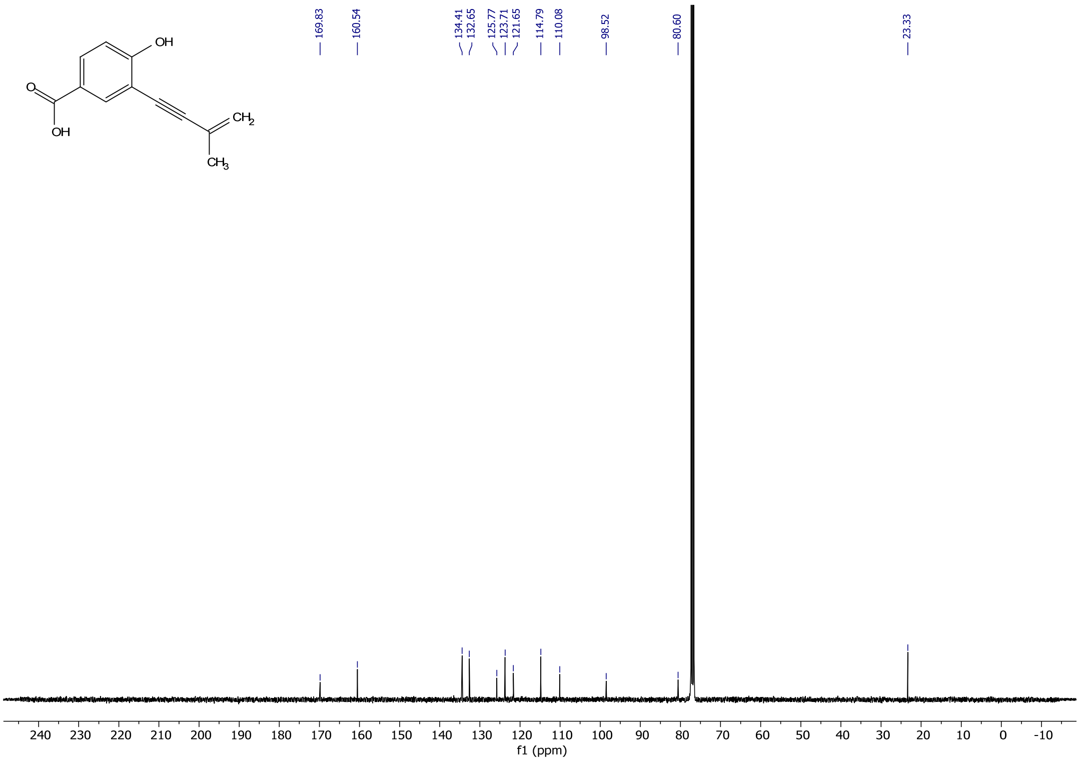

S16.- [13]C of Eutypinic acid (6) in $CDCl_3$.

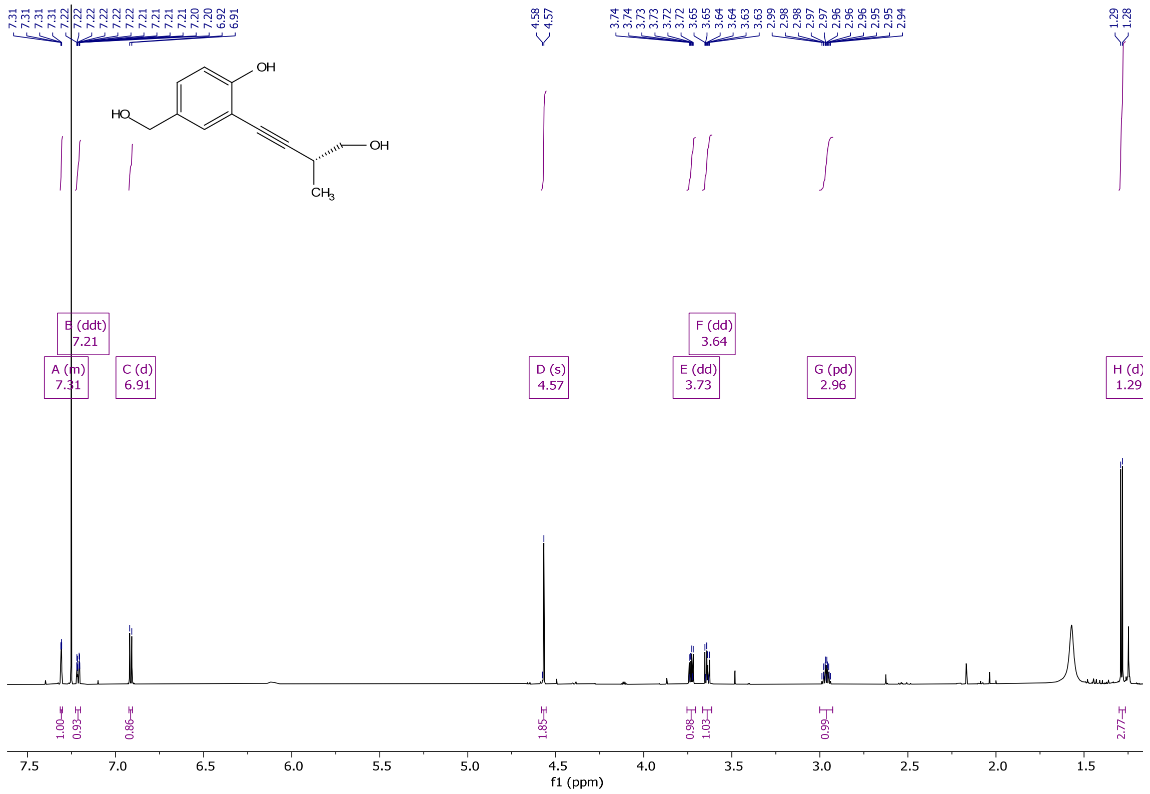

S17.- ^{1}H of **7** in $CDCl_3$.

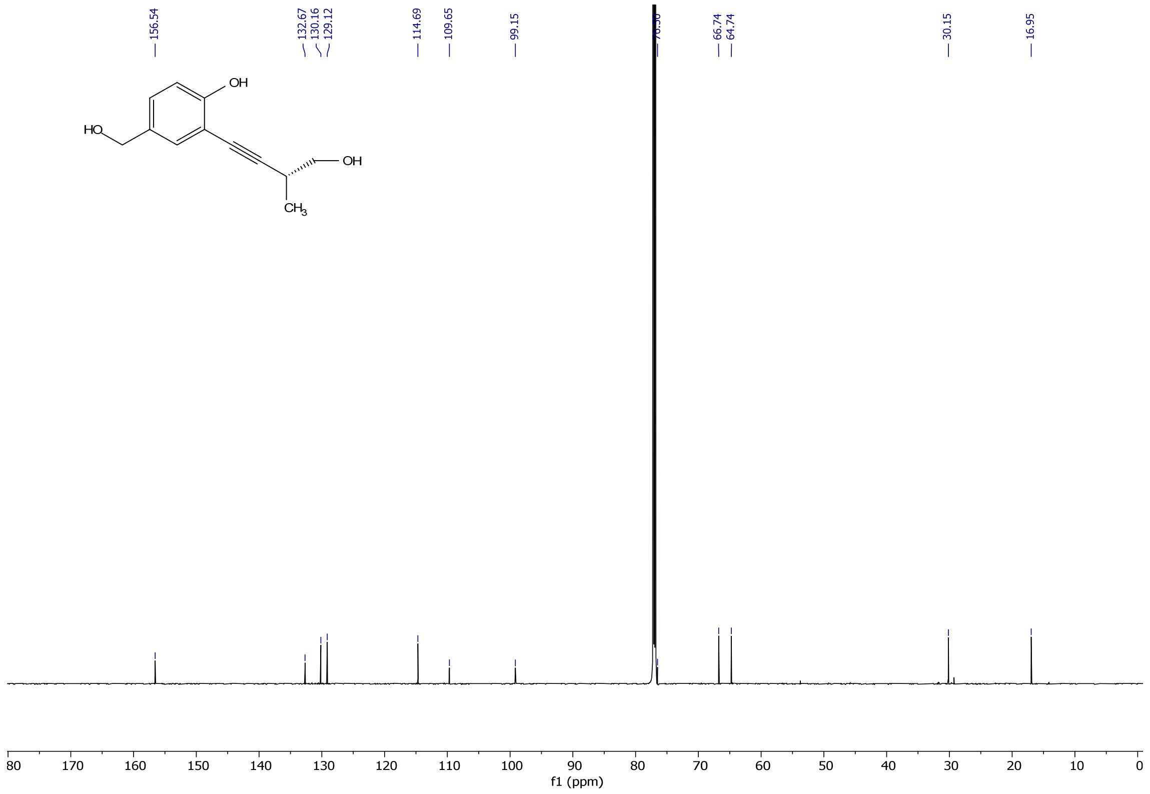

S18.- ^{13}C of **7** in $CDCl_3$.

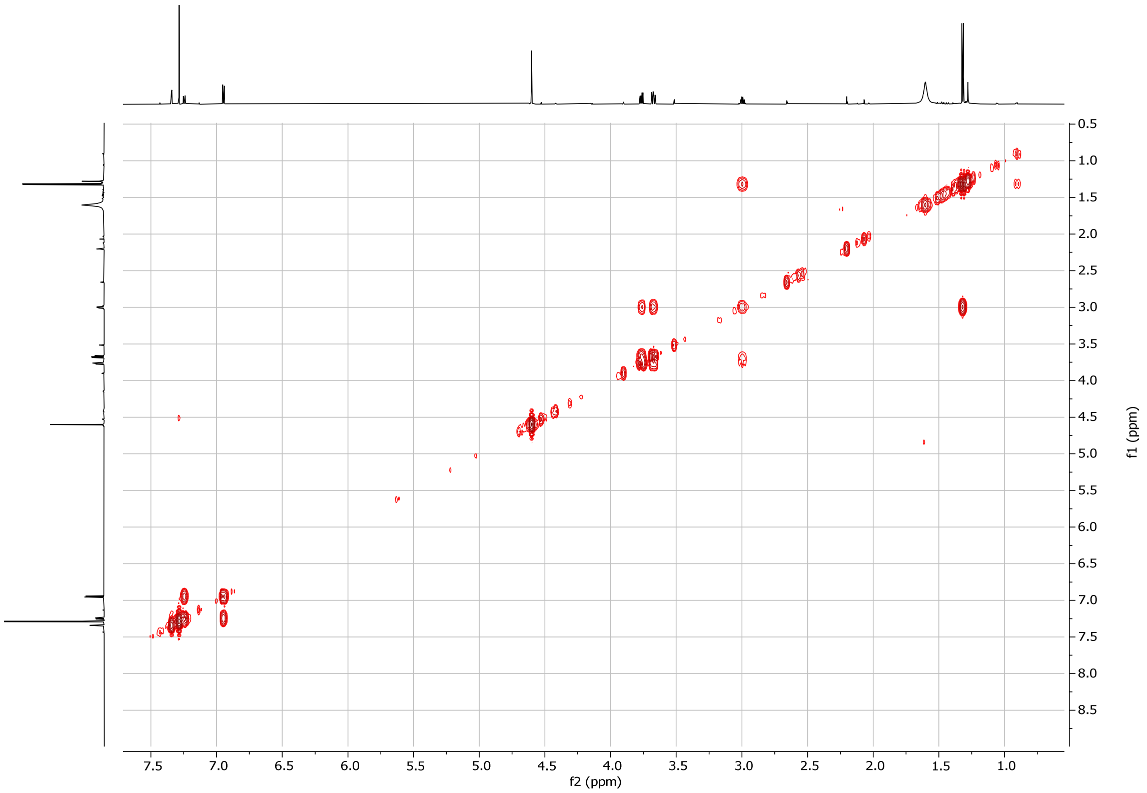

S19.- gCOSY of **7** in $CDCl_3$.

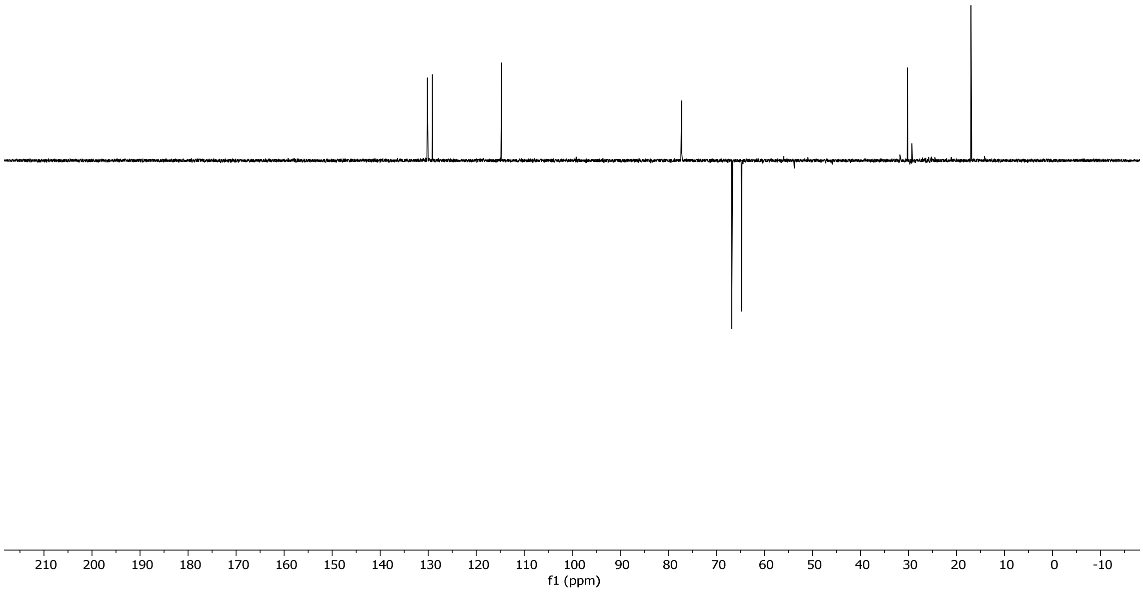

S20.- DEPT of **7** in $CDCl_3$.

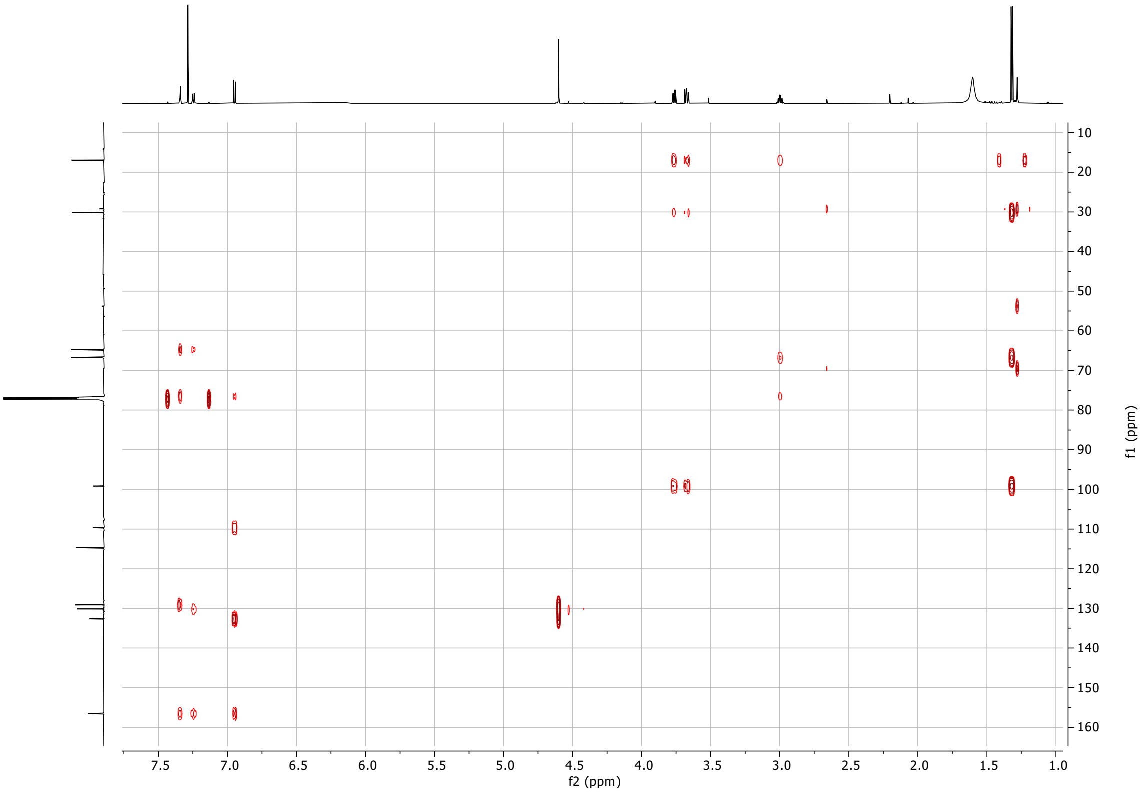

S21.- gHMBC of **7** in $CDCl_3$.

a)

Elemental Composition Report **Page 1**

Single Mass Analysis
Tolerance = 5.0 mDa / DBE: min = -1.5, max = 50.0
Element prediction: Off
Number of isotope peaks used for i-FIT = 3

Monoisotopic Mass, Even Electron Ions
57 formula(e) evaluated with 2 results within limits (up to 50 closest results for each mass)
Elements Used:
C: 0-500 H: 0-1000 O: 0-200 Na: 0-1
EL-6 25 (0.457)
1: TOF MS ES+

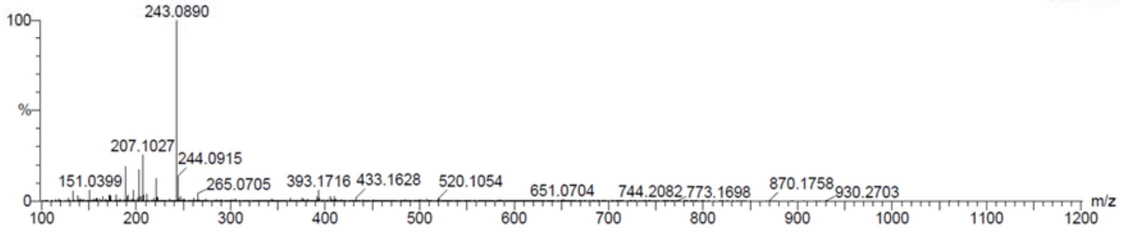

Mass	Calc. Mass	mDa	PPM	DBE	i-FIT	Norm	Conf(%)	Formula
Minimum:				-1.5				
Maximum:		5.0	10.0	50.0				
207.1027	207.1021	0.6	2.9	5.5	1560.1	0.010	99.02	C12 H15 O3
	207.0997	3.0	14.5	2.5	1564.7	4.624	0.98	C10 H16 O3 Na

b)

Elemental Composition Report **Page 1**

Single Mass Analysis
Tolerance = 5.0 mDa / DBE: min = -1.5, max = 50.0
Element prediction: Off
Number of isotope peaks used for i-FIT = 3

Monoisotopic Mass, Even Electron Ions
45 formula(e) evaluated with 2 results within limits (up to 50 closest results for each mass)
Elements Used:
C: 0-500 H: 0-1000 O: 0-200 Na: 0-1
EL-6 19 (0.354)
1: TOF MS ES+

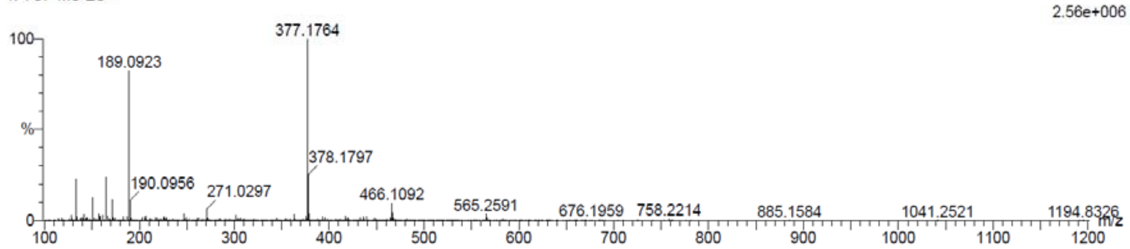

Minimum:				-1.5				
Maximum:		5.0	10.0	50.0				
Mass	Calc. Mass	mDa	PPM	DBE	i-FIT	Norm	Conf(%)	Formula
189.0923	189.0916	0.7	3.7	6.5	1766.2	0.005	99.50	C12 H13 O2
	189.0891	3.2	16.9	3.5	1771.5	5.306	0.50	C10 H14 O2 Na

S22.- MS of 7.

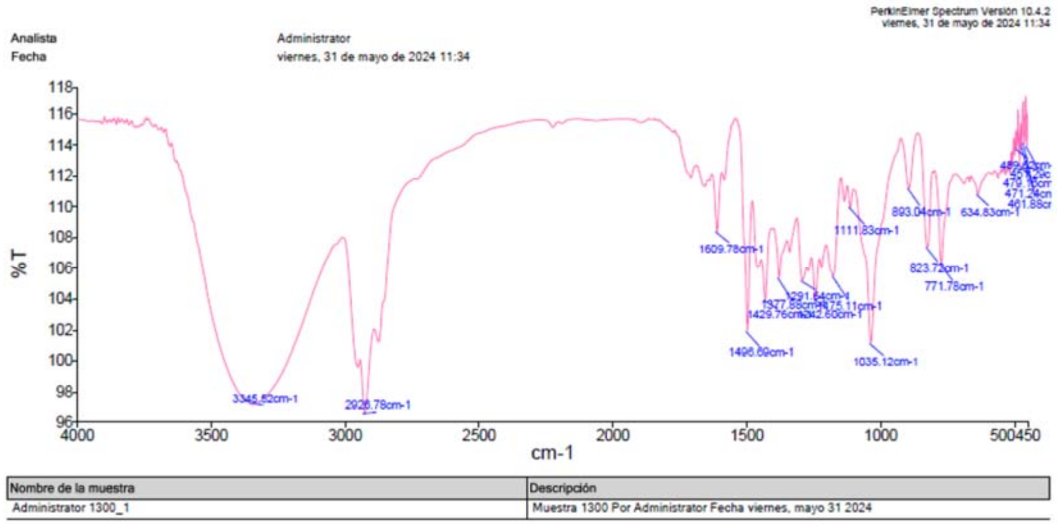

S23.- IR spectrum of 7.

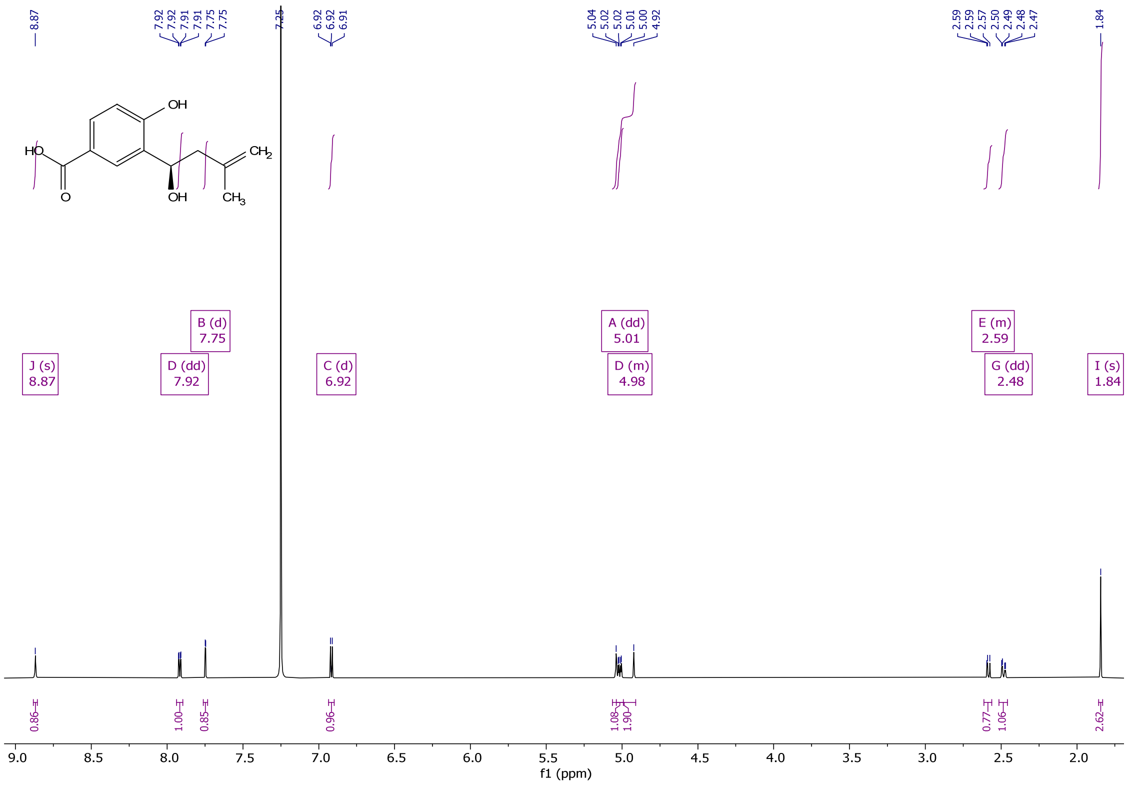

S24.- [1]H of **8** in $CDCl_3$.

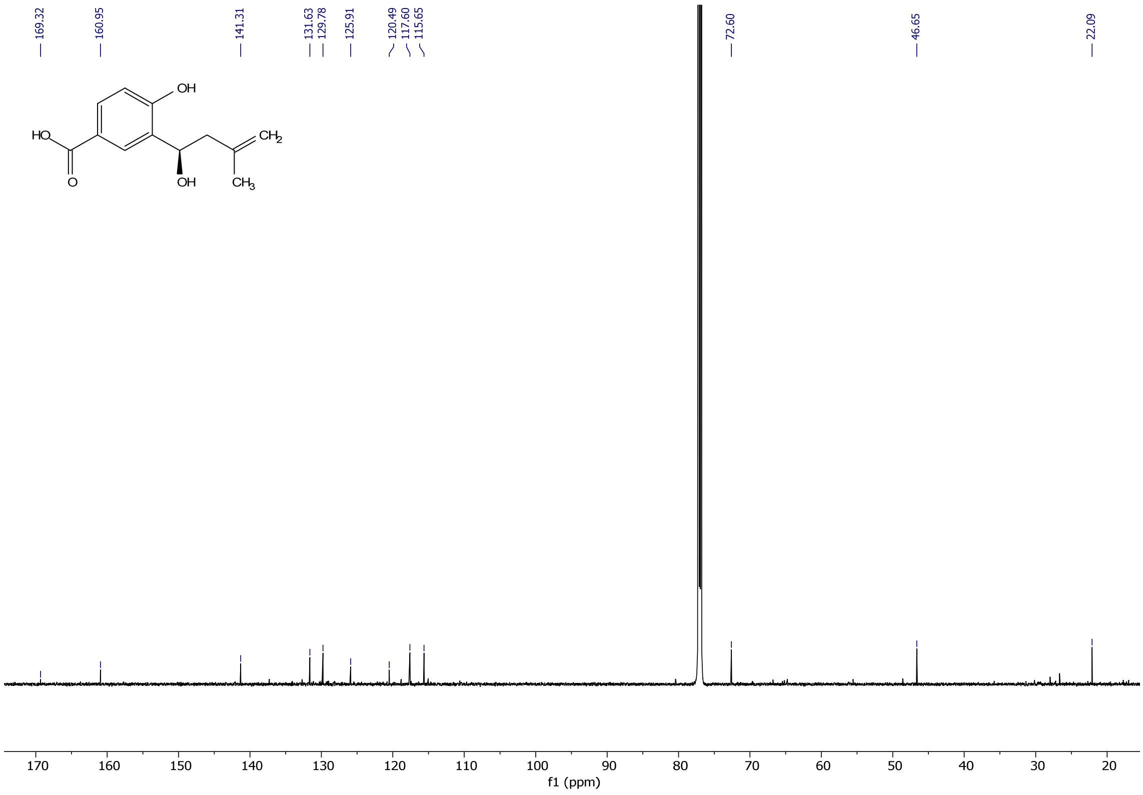

S25.- [13]C of **8** in $CDCl_3$.

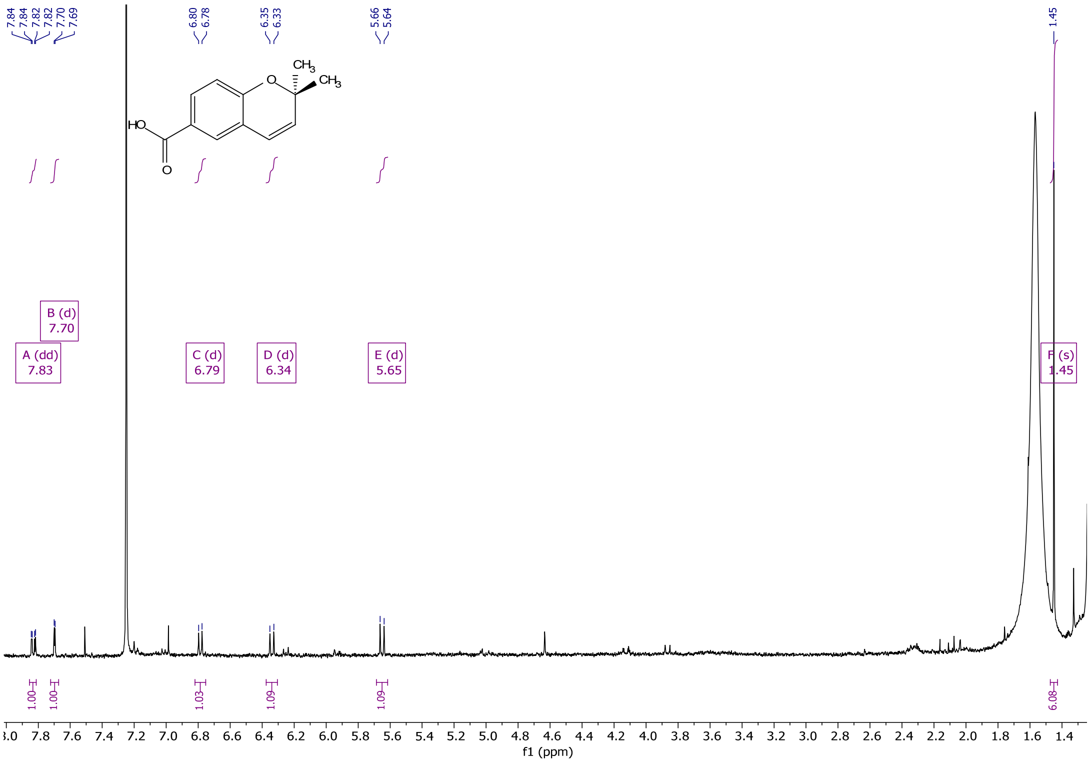

S26.- ^{1}H of **9** in $CDCl_3$.

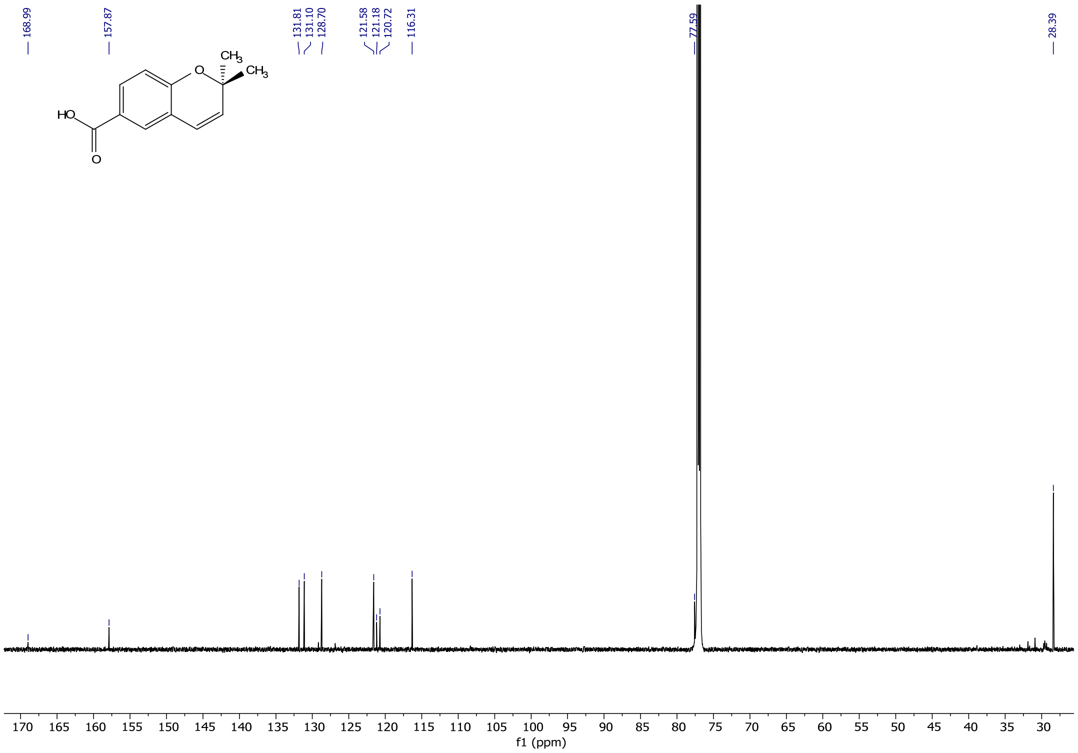

S27.- ^{13}C of **9** in $CDCl_3$.

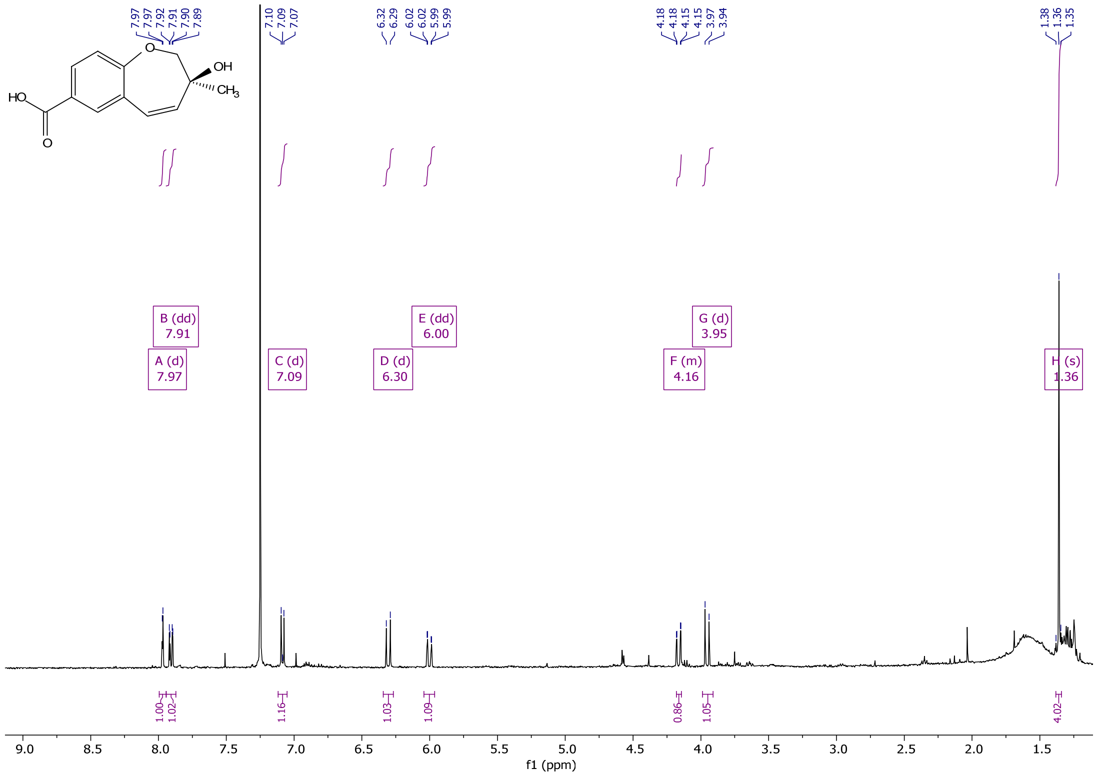

S28.- ^{1}H of **10** in $CDCl_3$.

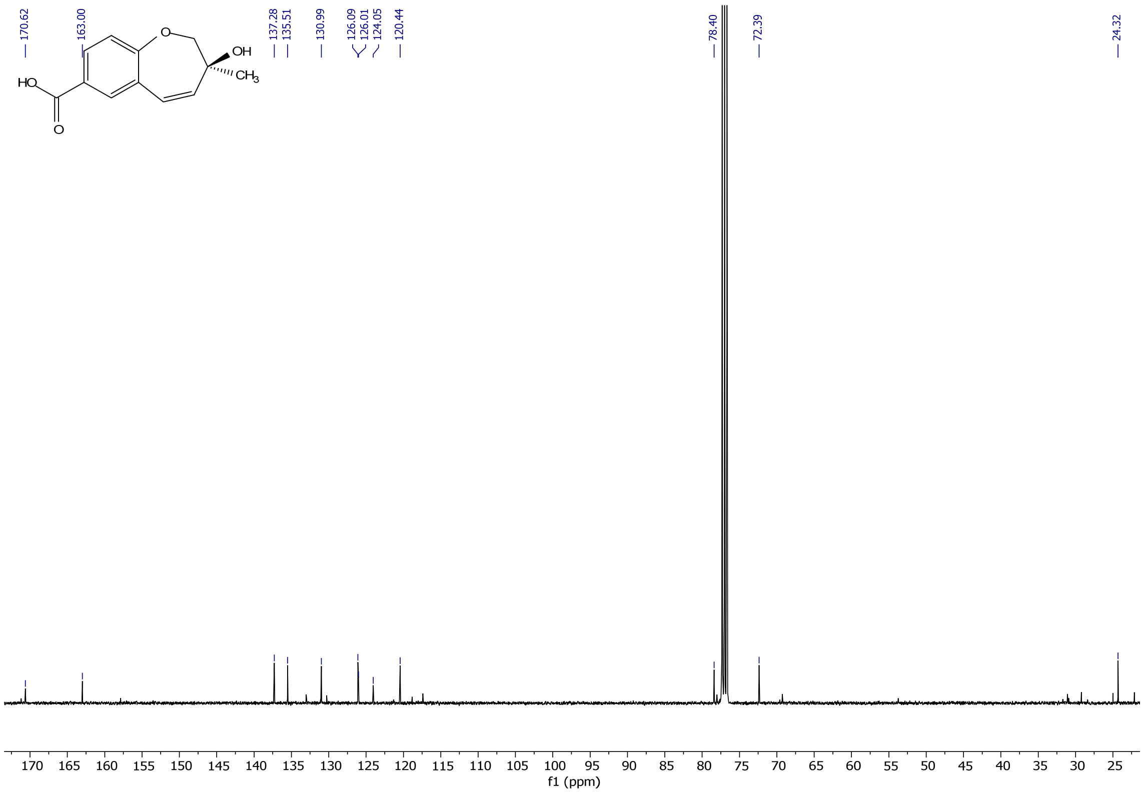

S29.- ^{13}C of **10** in $CDCl_3$.

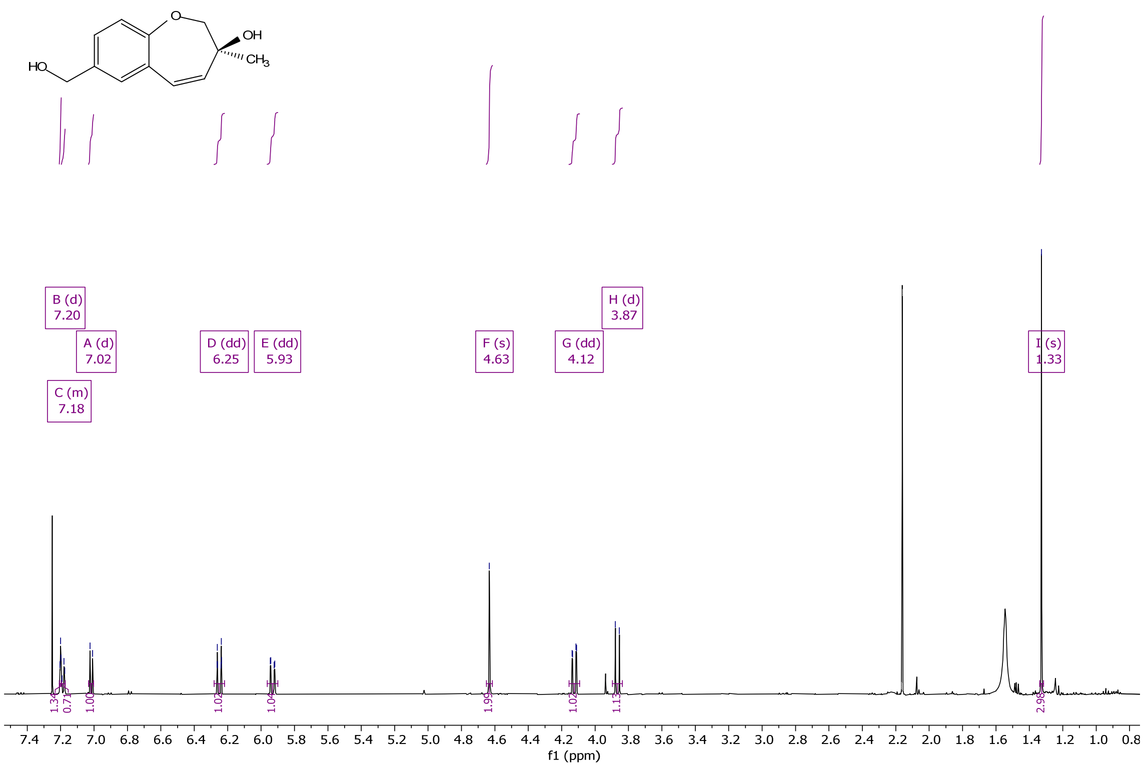

S30.- [1]H of **11** in $CDCl_3$.

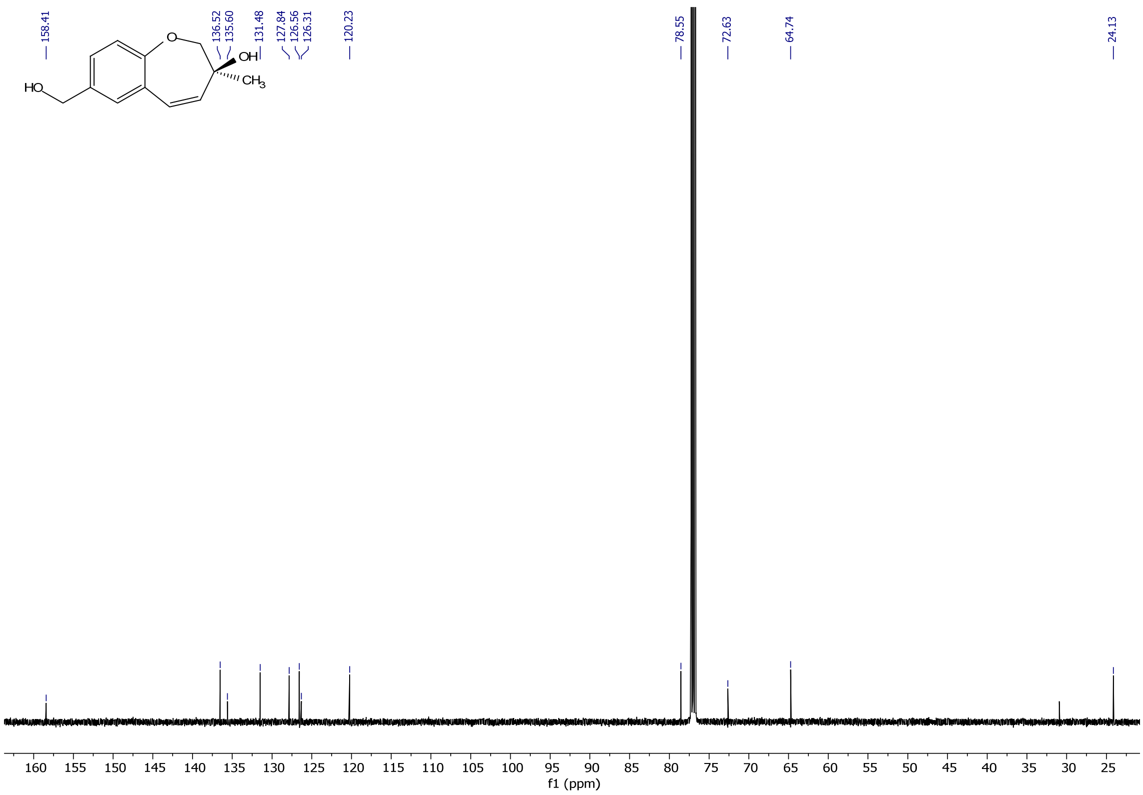

S31.- [13]C of **11** in $CDCl_3$.

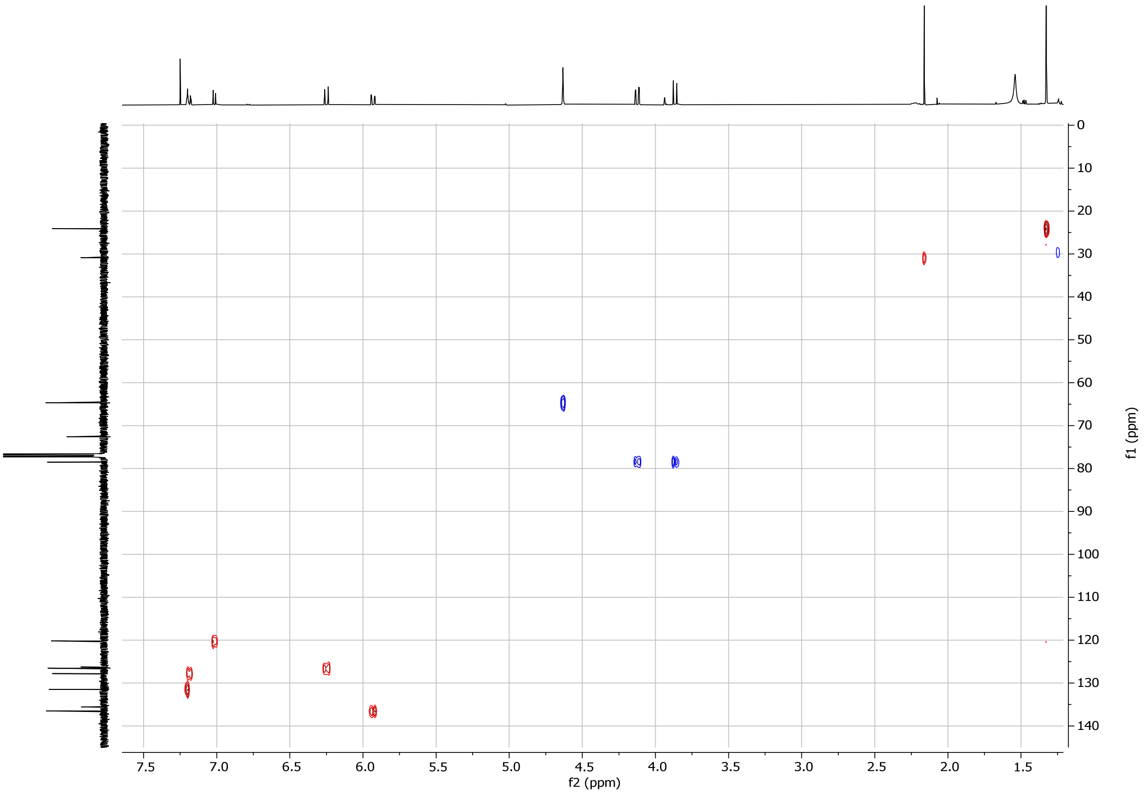

S32.- gHSQC of **11** in $CDCl_3$.

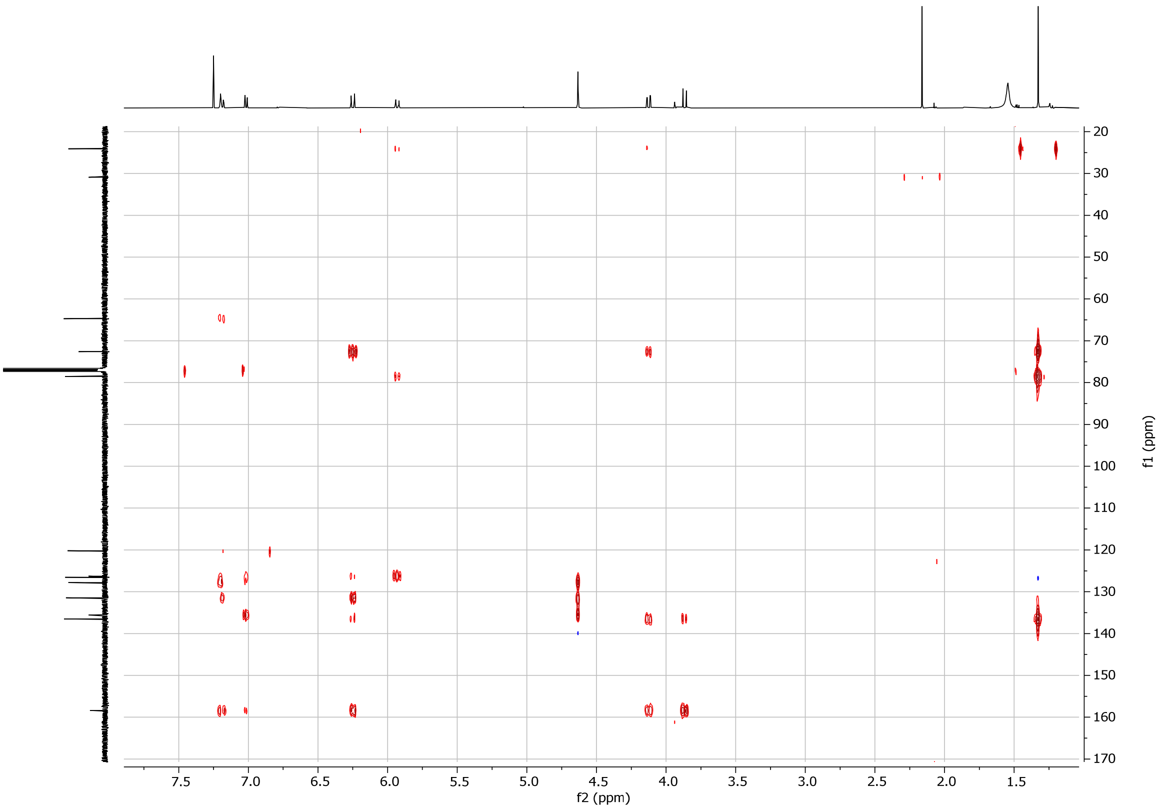

S33.- gHMBC of **11** in $CDCl_3$.

Elemental Composition Report **Page 1**

Single Mass Analysis
Tolerance = 5.0 mDa / DBE: min = -1.5, max = 50.0
Element prediction: Off
Number of isotope peaks used for i-FIT = 3

Monoisotopic Mass, Even Electron Ions
57 formula(e) evaluated with 2 results within limits (up to 50 best isotopic matches for each mass)
Elements Used:
C: 0-500 H: 0-1000 O: 0-200 Na: 0-1
EL_311_ARROZ_80_6-DIL 365 (3.285)
1: TOF MS ES+

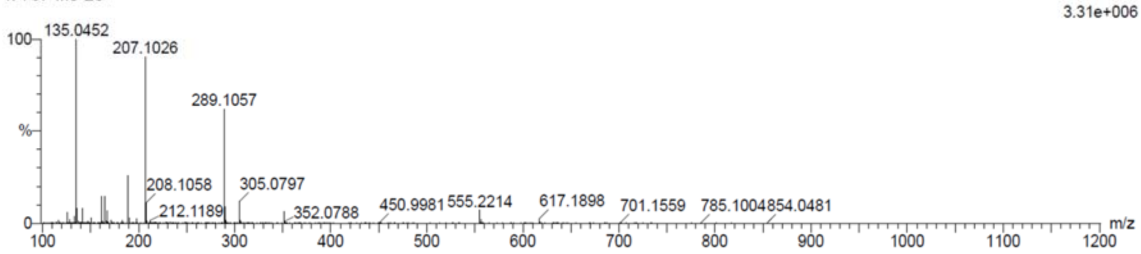

Minimum:				-1.5				
Maximum:		5.0	10.0	50.0				
Mass	Calc. Mass	mDa	PPM	DBE	i-FIT	Norm	Conf(%)	Formula
207.1026	207.1021	0.5	2.4	5.5	1531.9	0.080	92.35	C12 H15 O3
	207.0997	2.9	14.0	2.5	1534.4	2.570	7.65	C10 H16 O3 Na

S34.- MS of **11** in $CDCl_3$.

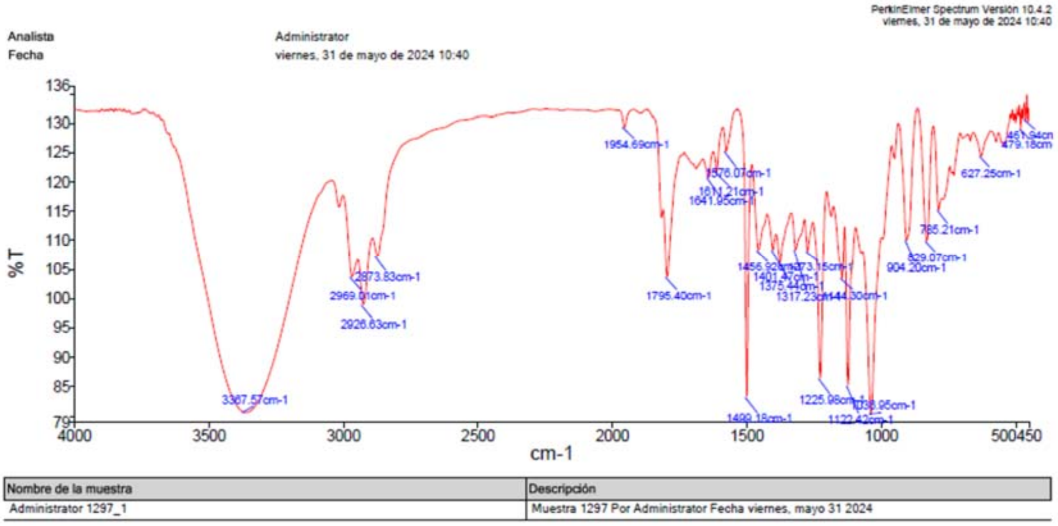

S35.- IR spectra of **11**.

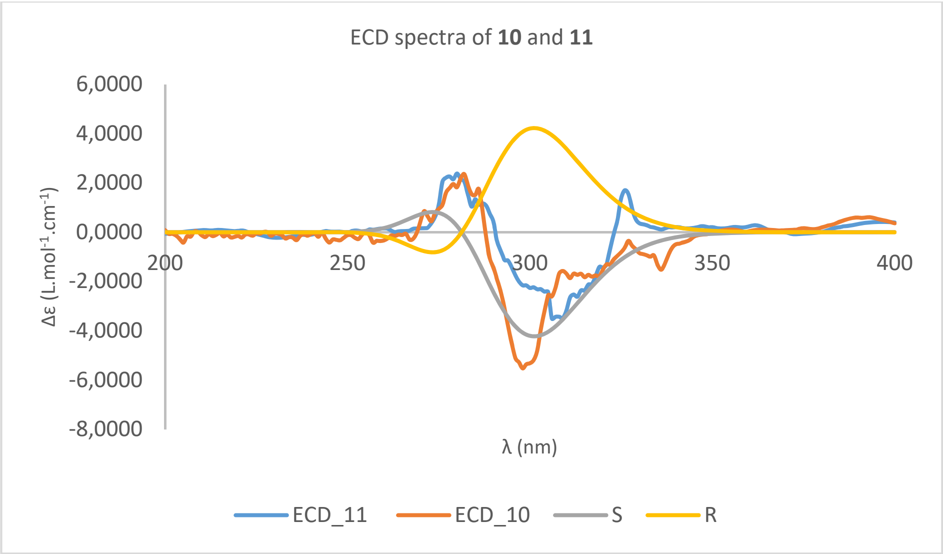

S36.- Comparison of ECD spectra of **10** and **11**.

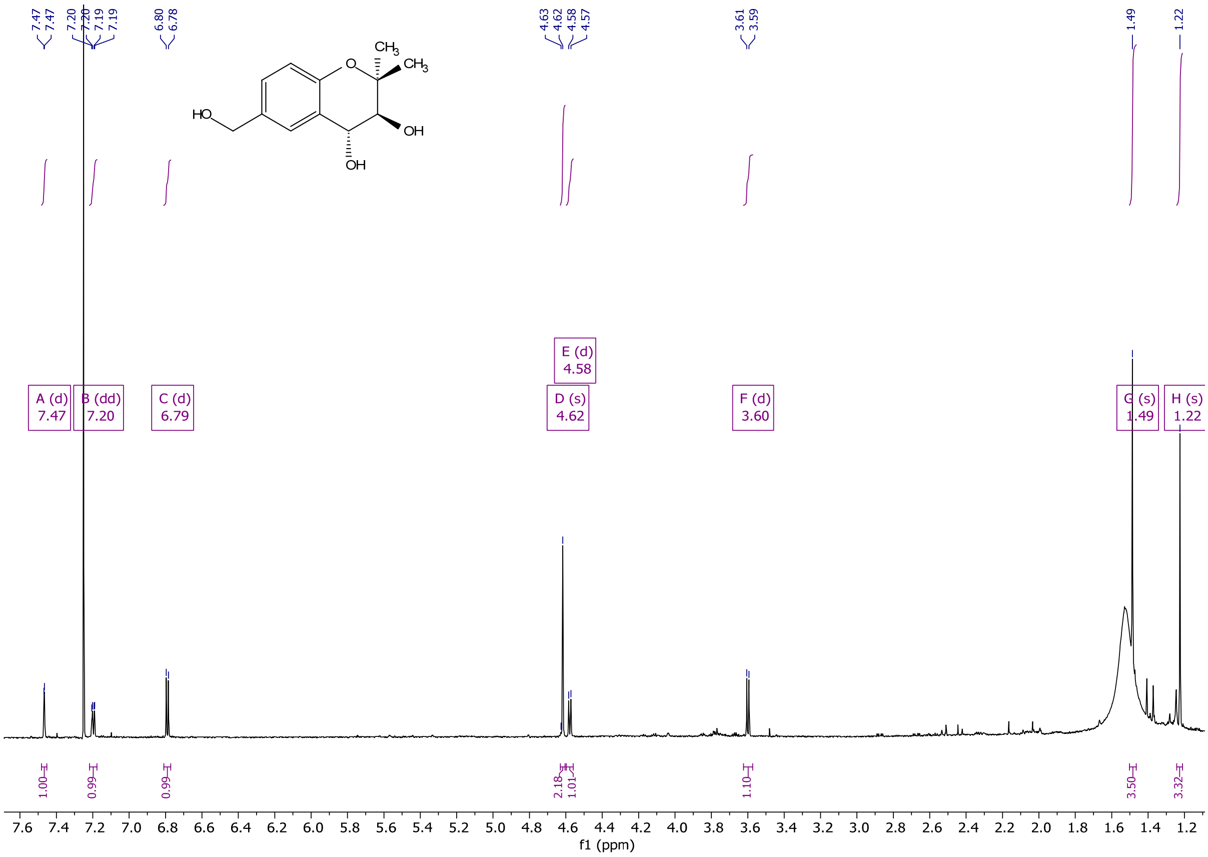

S37.- ^{1}H of **12** in $CDCl_3$.

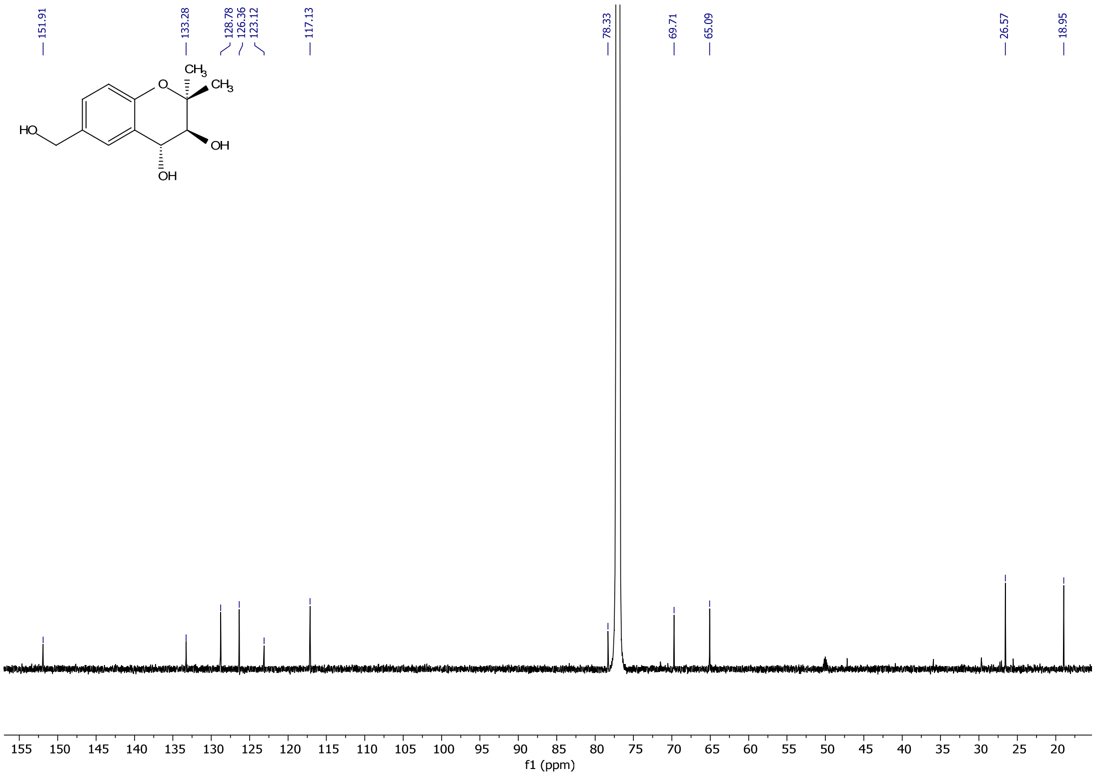

S38.- ^{13}C of **12** in $CDCl_3$.

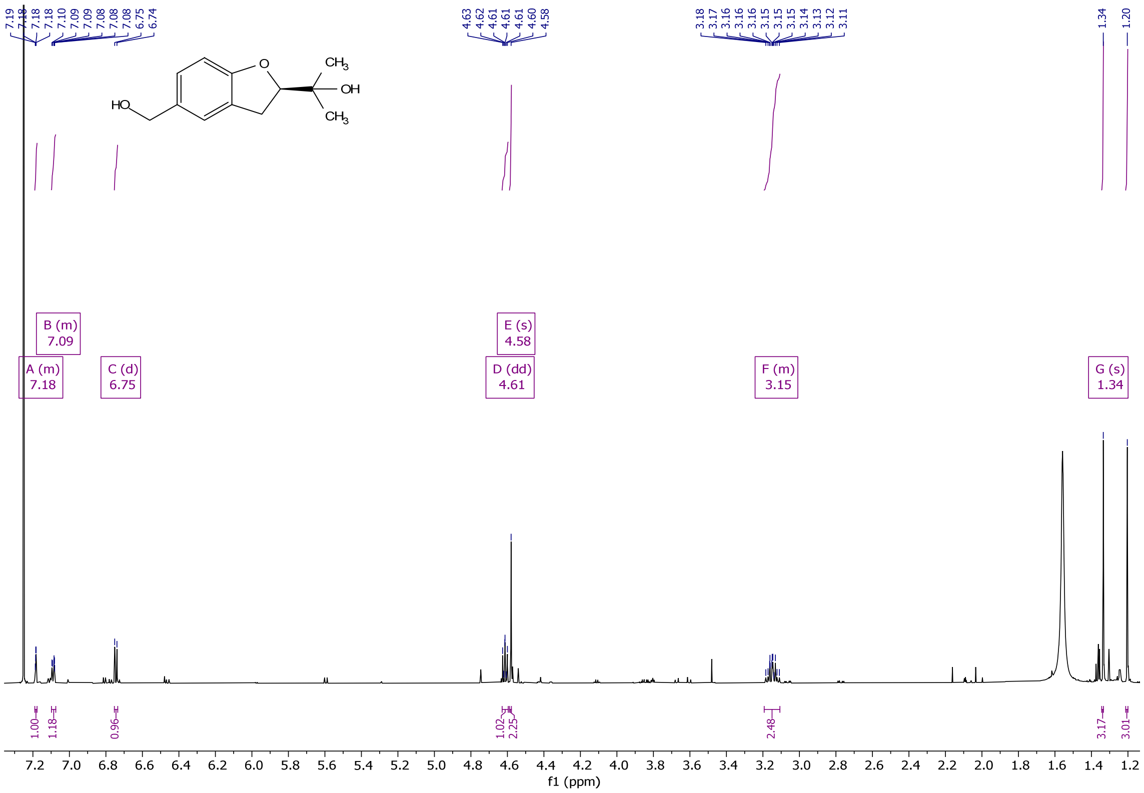

S39.- ^{1}H of **13** in $CDCl_3$.

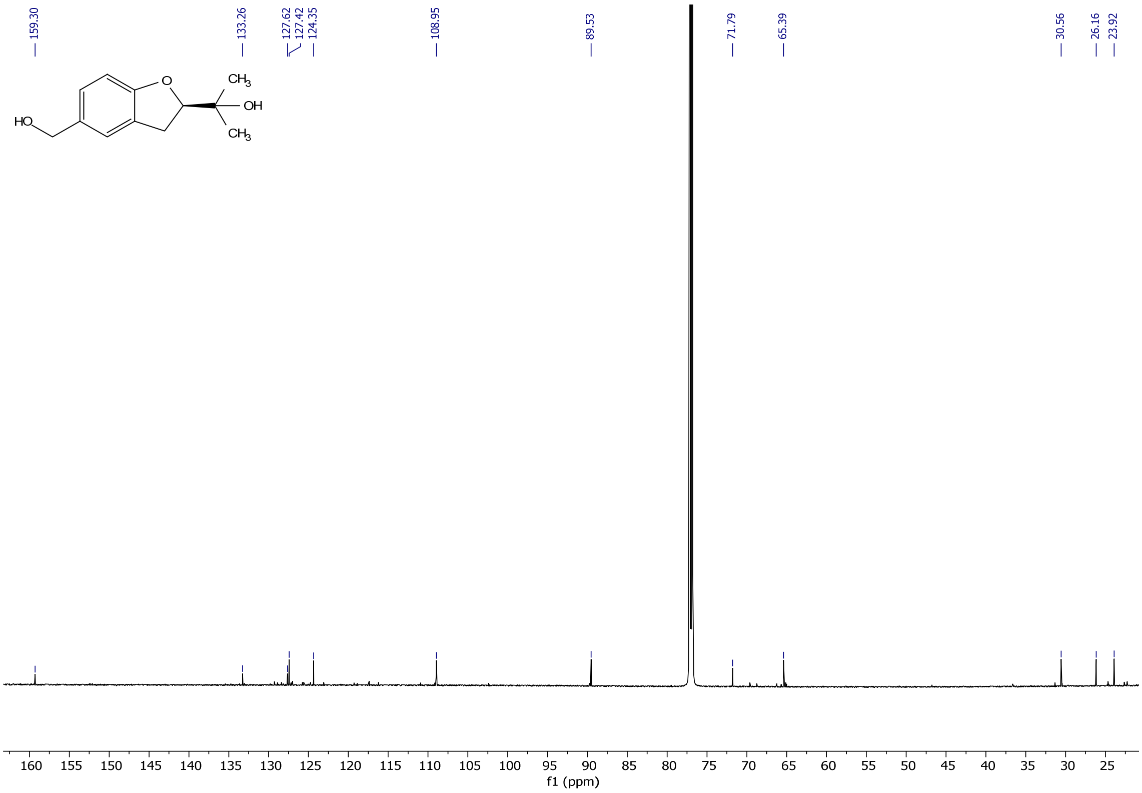

S40.- ^{13}C of **13** in $CDCl_3$.

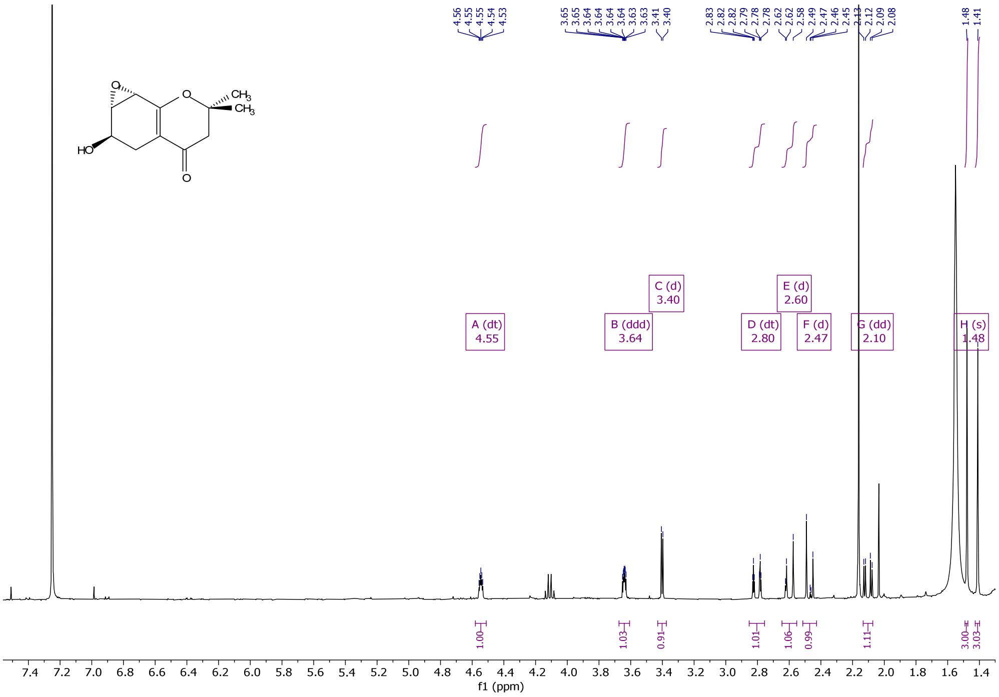

S41.- ^{1}H of **14** in $CDCl_3$.

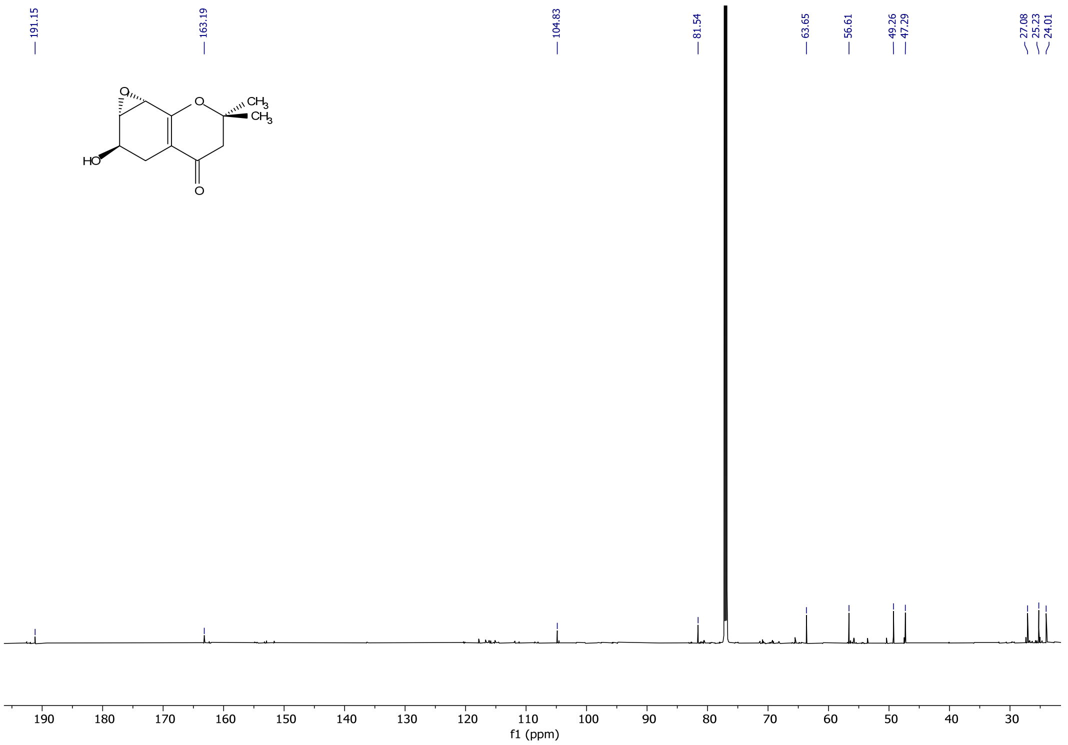

S42.- ^{13}C of **14** in $CDCl_3$.

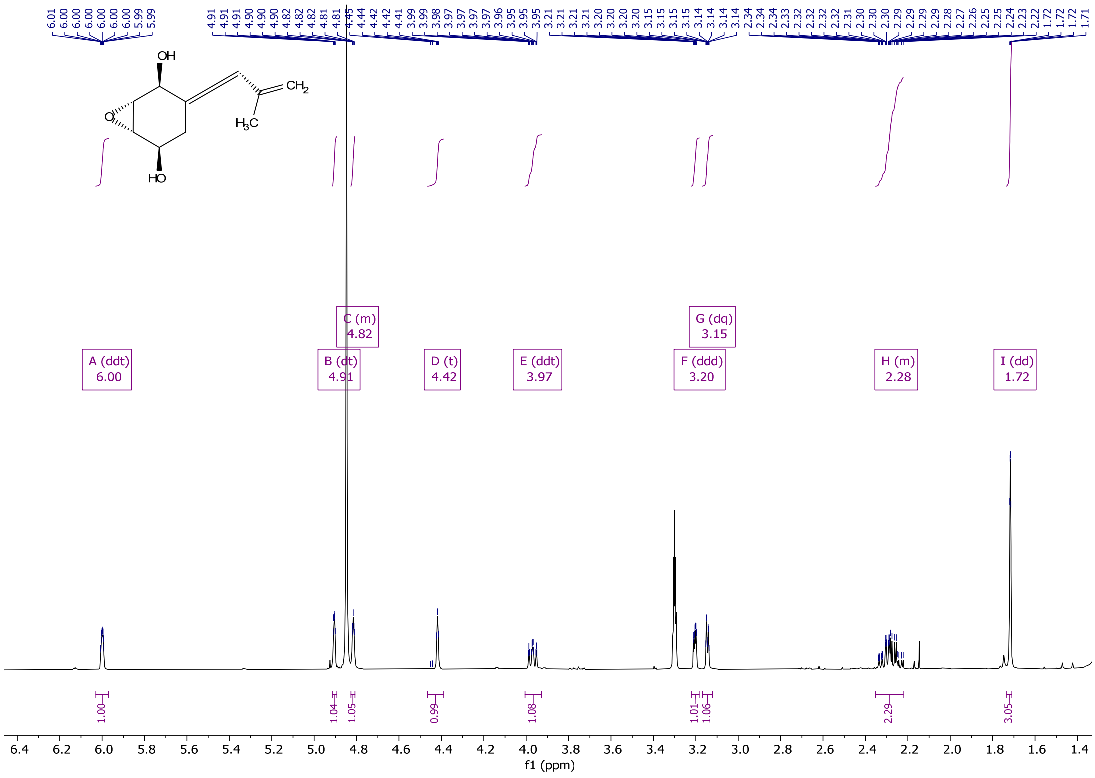

S43.- ^{1}H of **15** in CD_3OD.

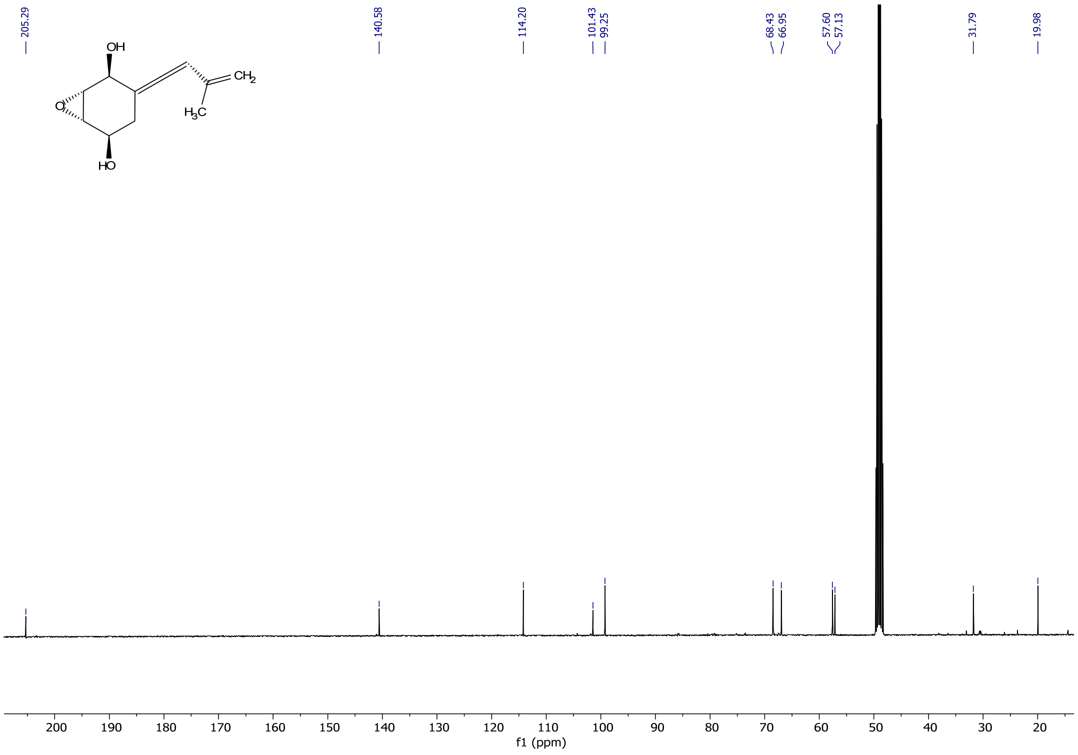

S44.- ^{13}C of **15** in CD_3OD.

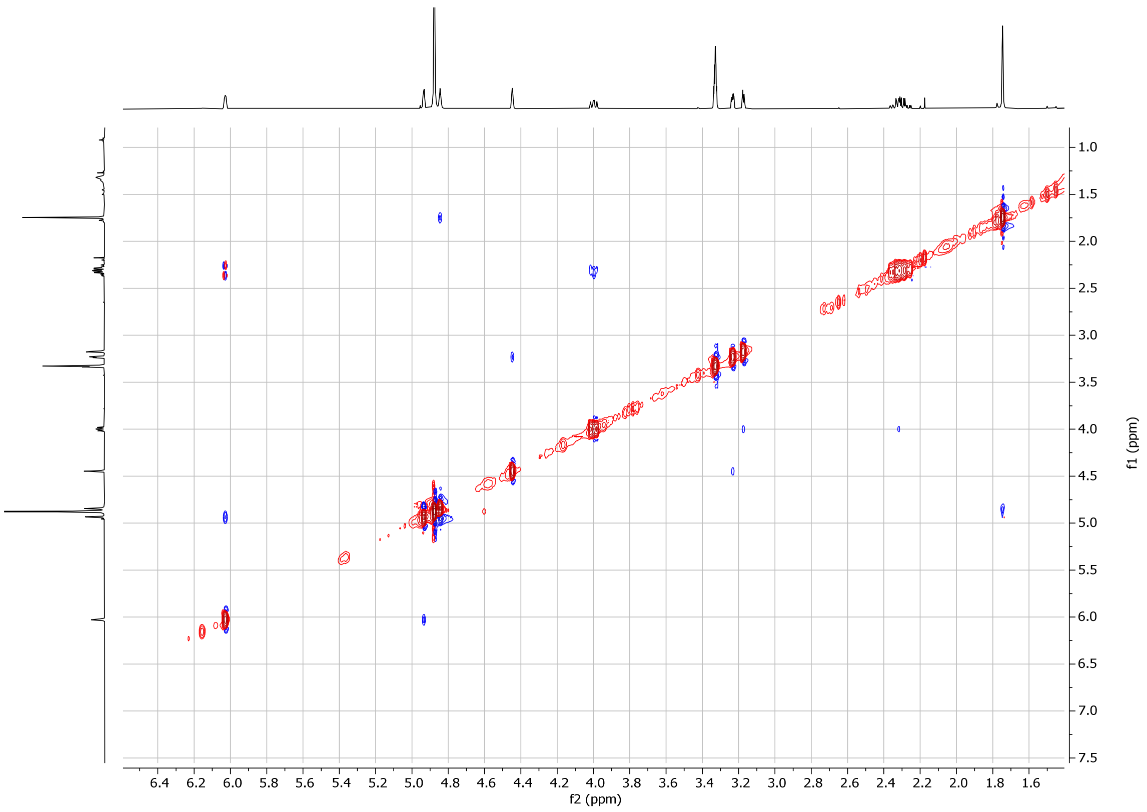

S45.- NOESY of **15** in CD_3OD.

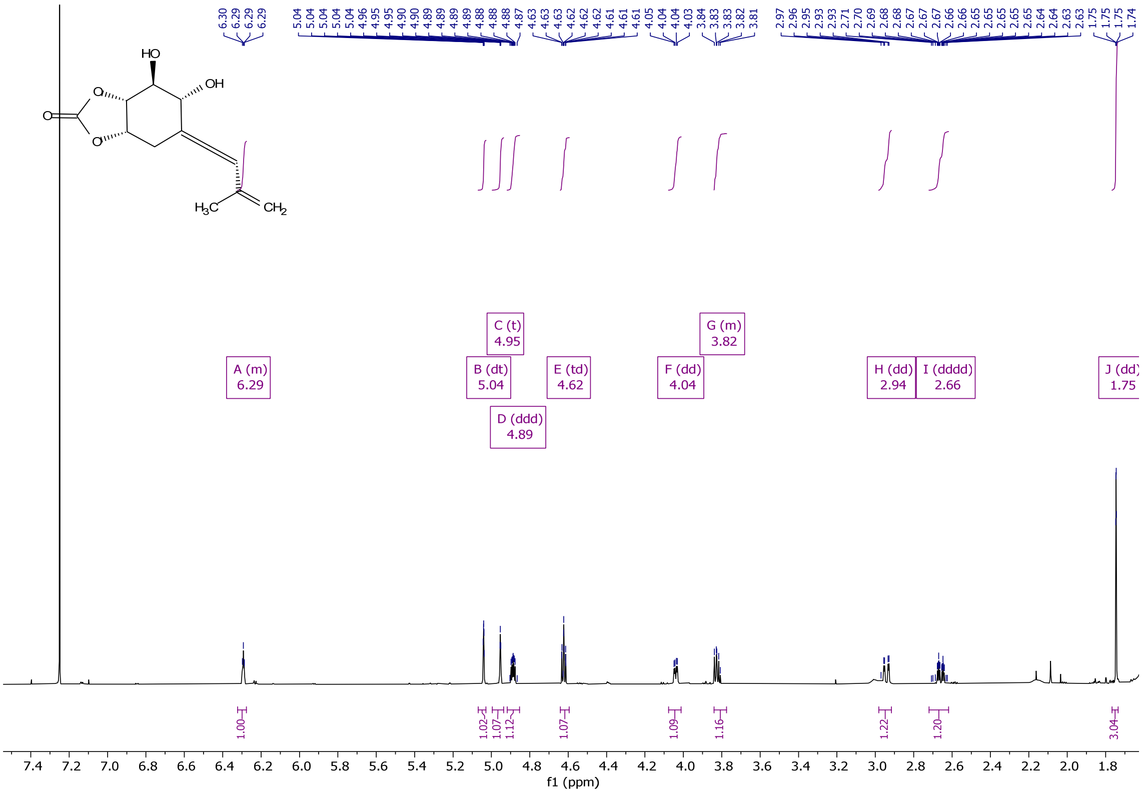

S46.- [1]H of **16** in $CDCl_3$.

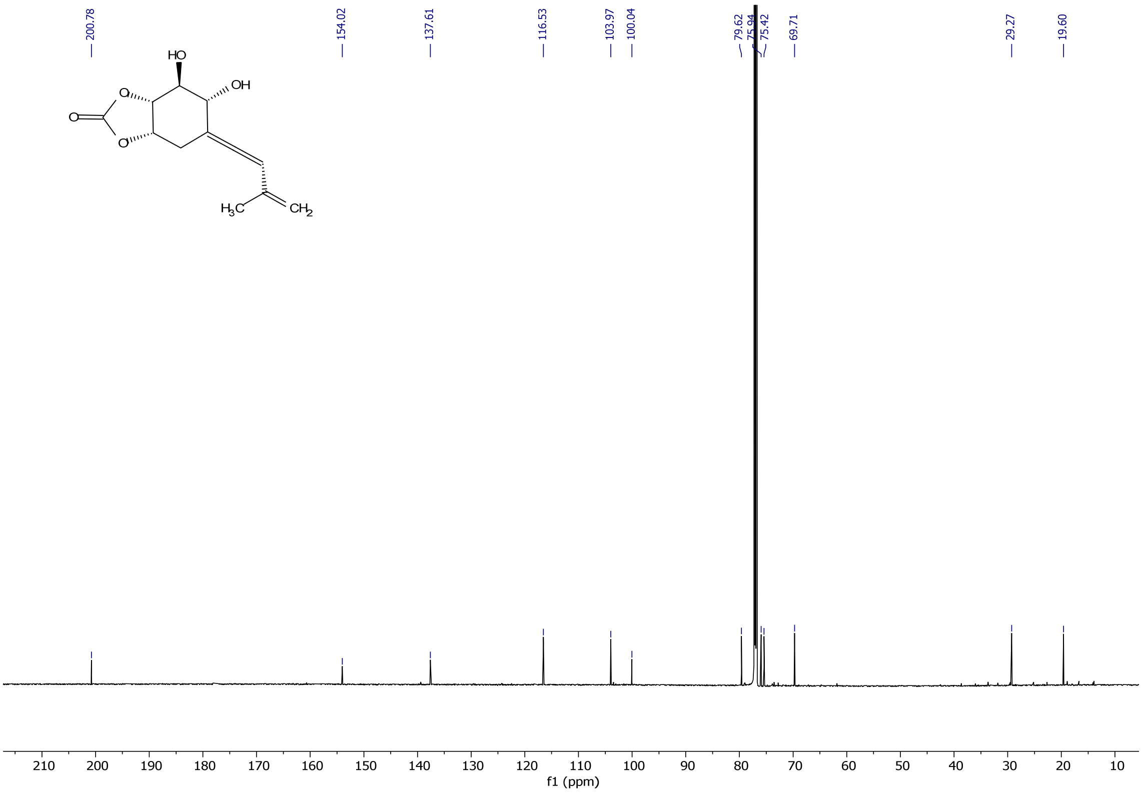

S47.- [13]C of **16** in $CDCl_3$.

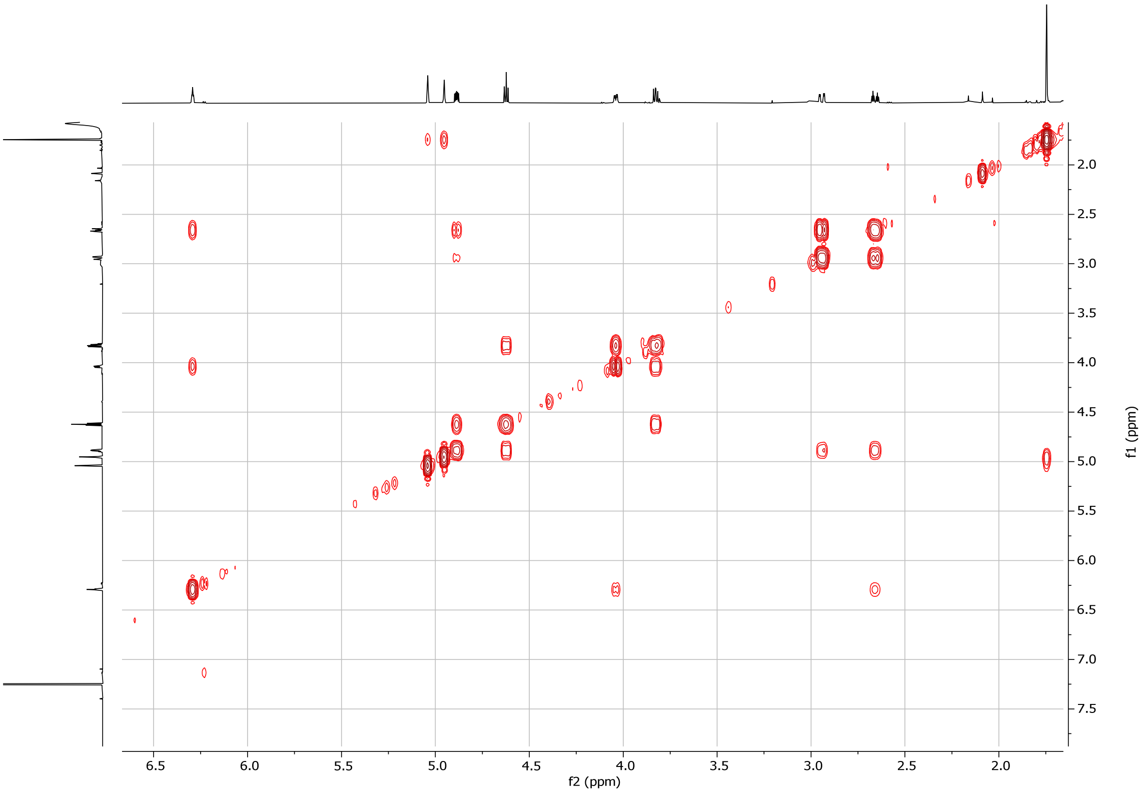

S48.- gCOSY of **16** in $CDCl_3$.

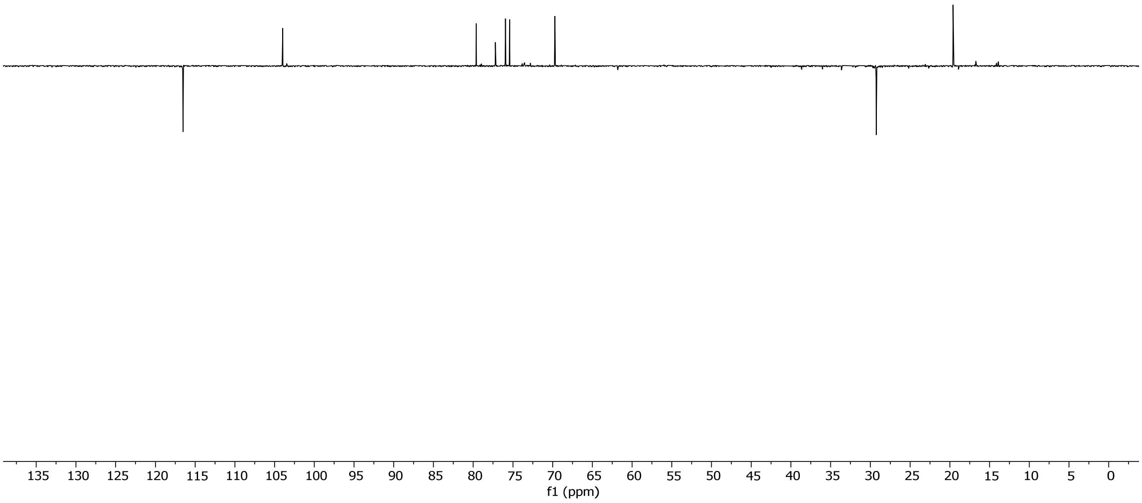

S49.- DEPT of **16** in $CDCl_3$.

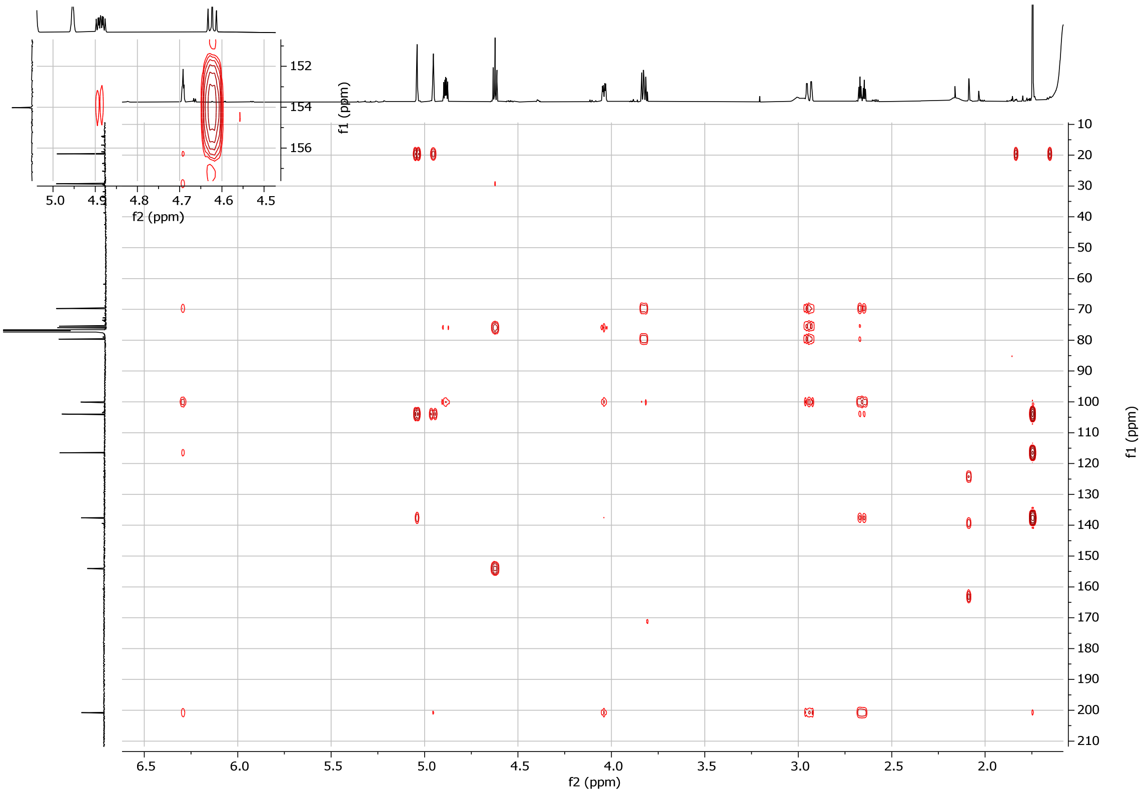

S50.- gHMBC of **16** in $CDCl_3$.

Elemental Composition Report **Page 1**

Single Mass Analysis
Tolerance = 5.0 mDa / DBE: min = -1.5, max = 50.0
Element prediction: Off
Number of isotope peaks used for i-FIT = 3

Monoisotopic Mass, Even Electron Ions
36 formula(e) evaluated with 1 results within limits (up to 50 closest results for each mass)
Elements Used:
C: 0-500 H: 0-1000 O: 0-200
EL-4-rep 41 (0.751)
1: TOF MS ES+

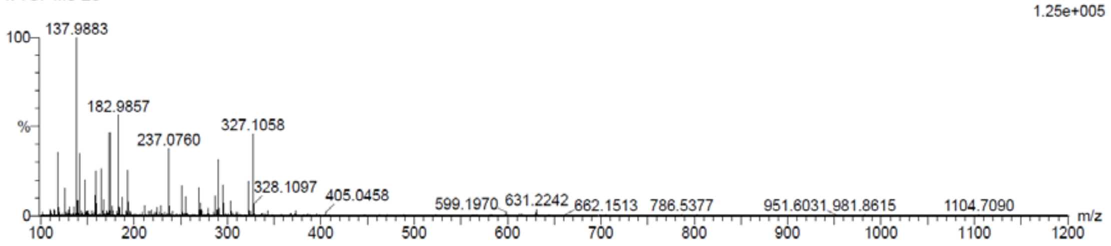

Minimum:				-1.5				
Maximum:		5.0	10.0	50.0				
Mass	Calc. Mass	mDa	PPM	DBE	i-FIT	Norm	Conf(%)	Formula
237.0760	237.0763	-0.3	-1.3	6.5	646.6	n/a	n/a	C12 H13 O5

S51.- MS of **16** in $CDCl_3$.

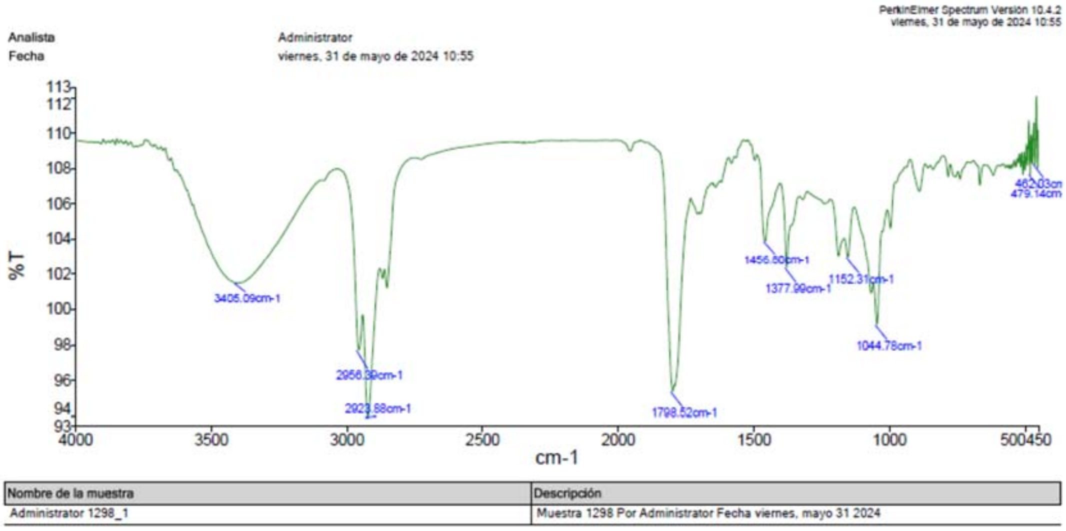

S52.- IR spectrum of 16.

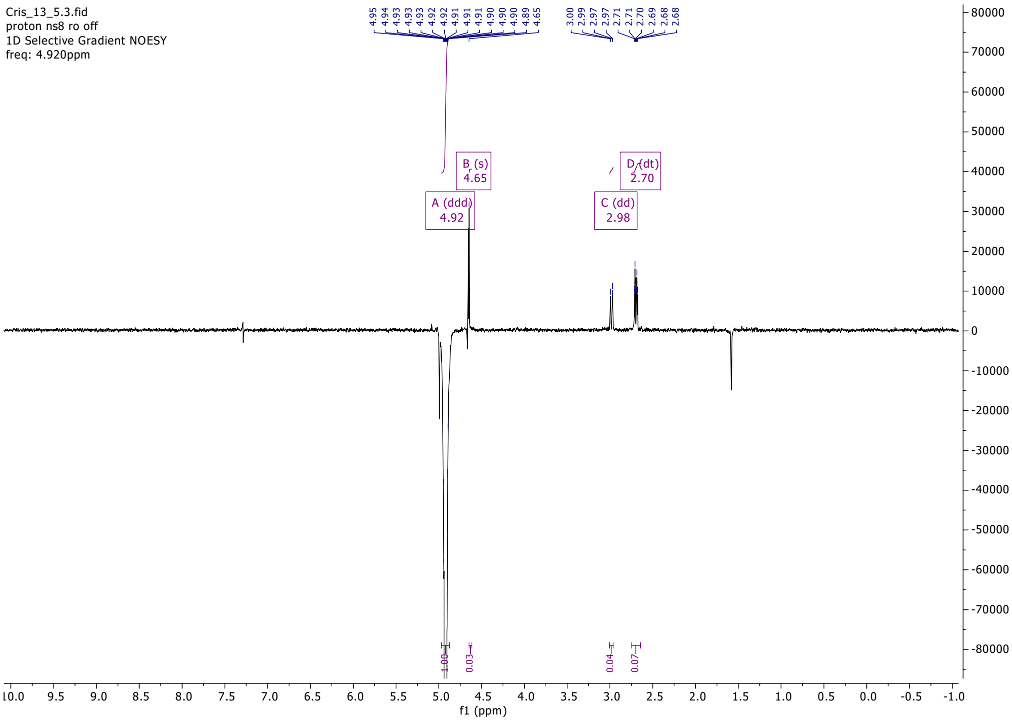

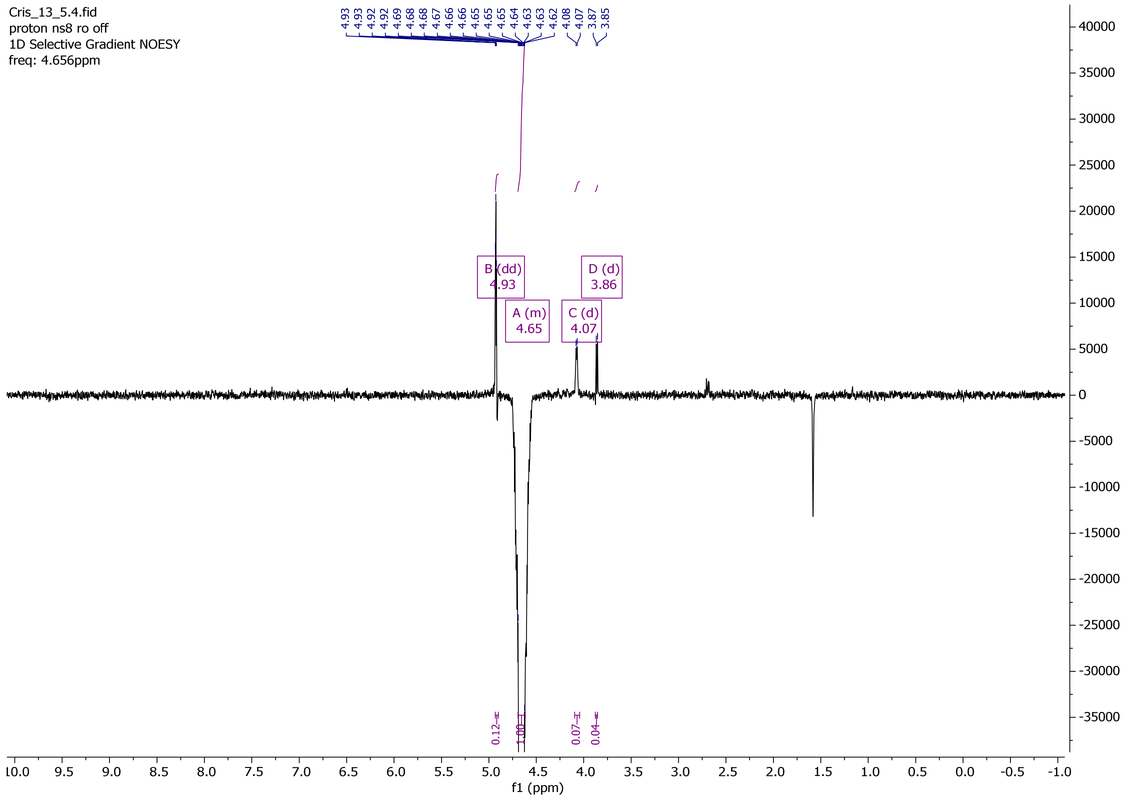

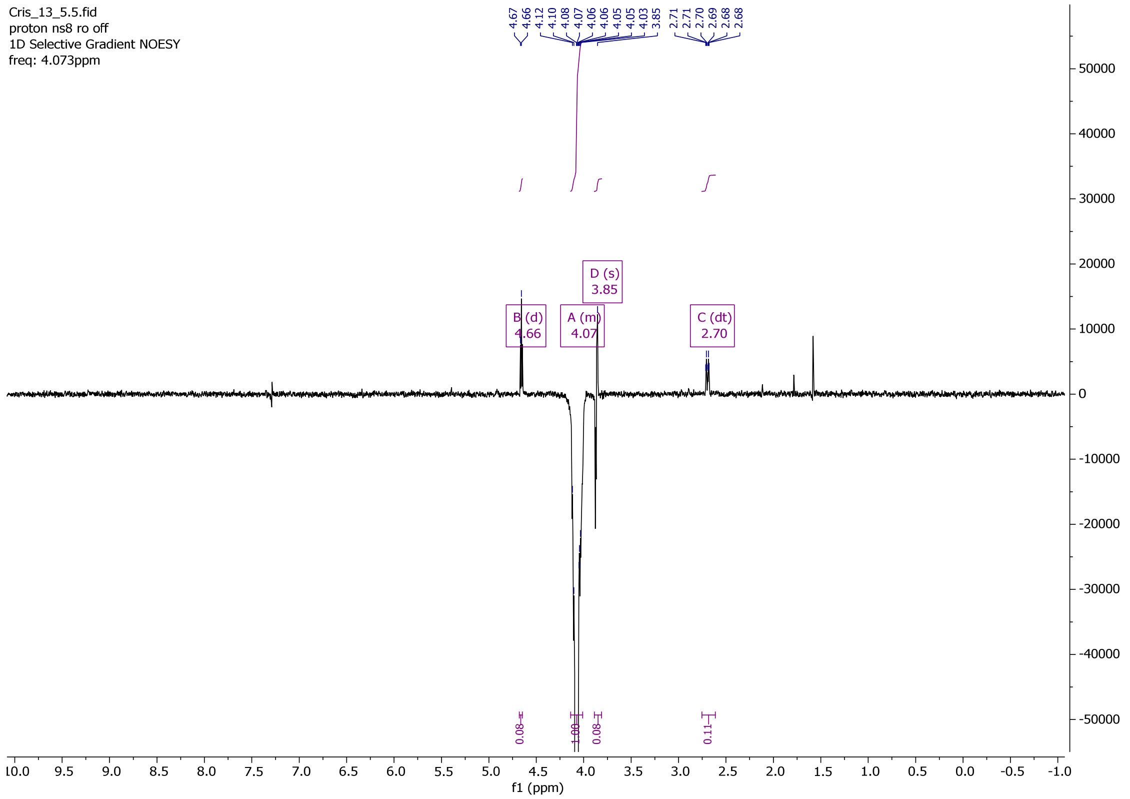

S53.- Selected NOEs spectra of **16**.

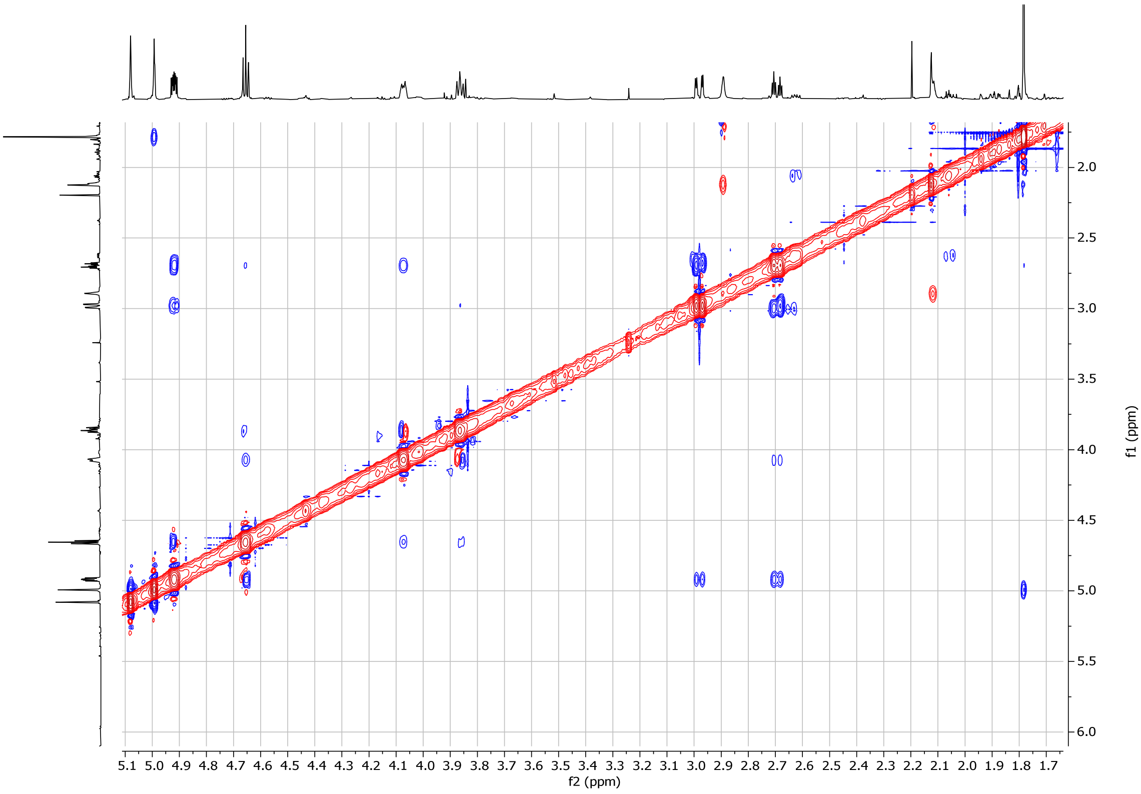
f1 (ppm)
f2 (ppm)

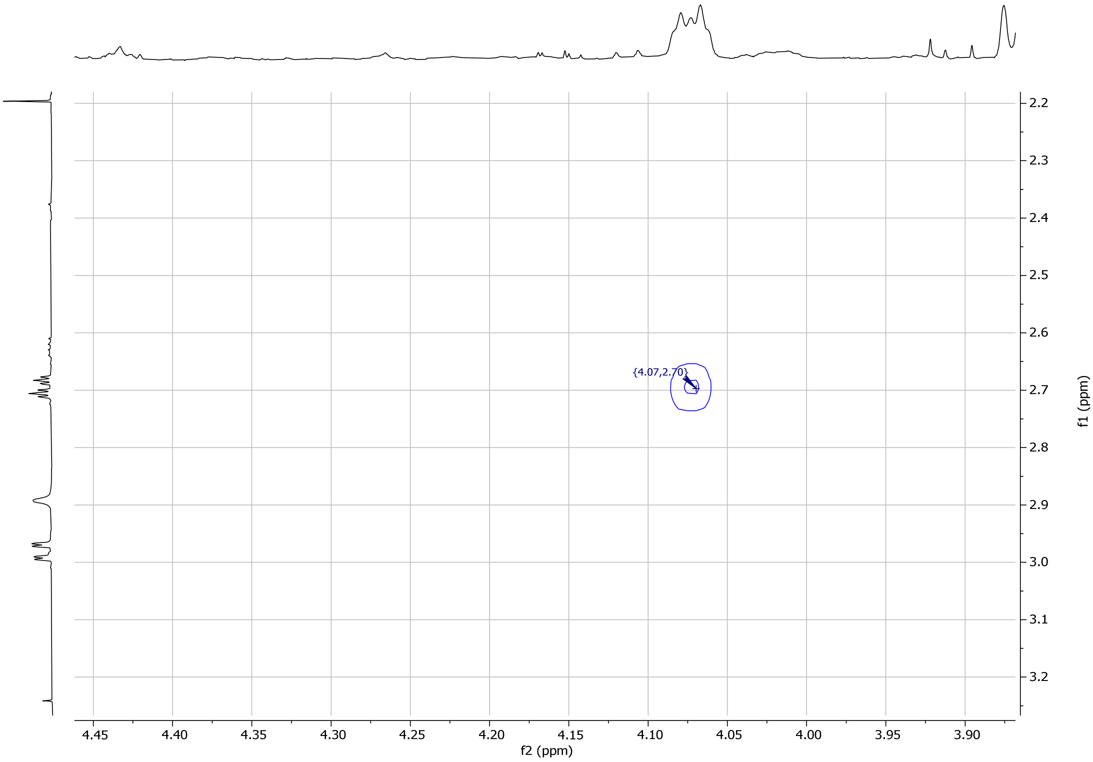
{4.07,2.70}
f1 (ppm)
f2 (ppm)

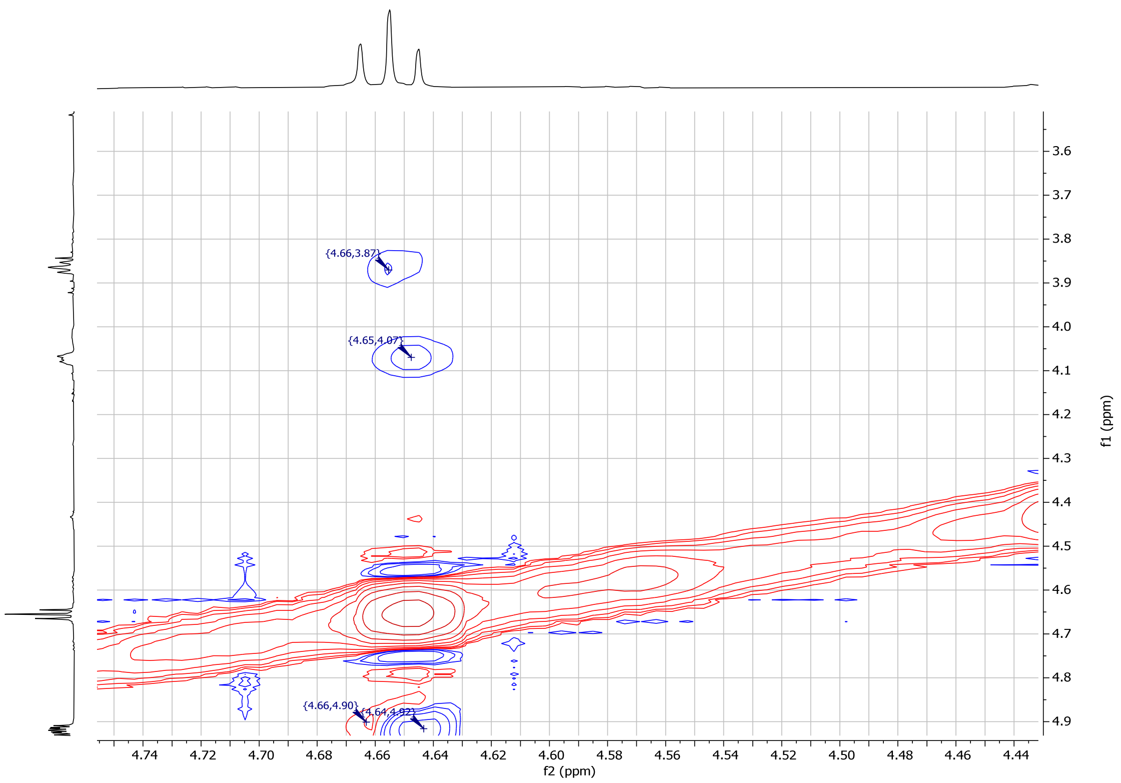

S54.- NOESY of **16**.
